# Modelling longitudinal and time-to-event data: a phase IV simulation study comparing R package implementations of joint models with time-varying Cox proportional-hazards regression, and the two-stage approach

**DOI:** 10.1186/s12874-026-02875-4

**Published:** 2026-05-14

**Authors:** Jil Heege, Sonja Greven, Elke Schaeffner, Ulrike Grittner, Annette Aigner

**Affiliations:** 1 Institute of Biometry and Clinical Epidemiology, Charité – Universitätsmedizin Berlin, corporate member of Freie Universität Berlin and Humboldt-Universität zu Berlin, Charitéplatz 1, Berlin, 10117 Germany; 2 Berlin Institute of Health at Charité - Universitätsmedizin Berlin, Berlin, Germany; 3 School of Business and Economics, Chair of Statistics, Humboldt-Universität zu Berlin, Unter den Linden 6, Berlin, 10099 Germany; 4 Institute of Public Health, Charité – Universitätsmedizin Berlin, corporate member of Freie Universität Berlin and Humboldt-Universität zu Berlin, Charitéplatz 1, Berlin, 10117 Germany; 5 Center for Stroke Research Berlin, Corporate Member of Freie Universität Berlin, Humboldt-Universität zu Berlin, Charité—Universitätsmedizin Berlin, Berlin, Germany

**Keywords:** Joint models, Two-stage approach, Longitudinal data, Time-varying covariates, Time-to-event analysis, Simulation study

## Abstract

Joint models offer an unbiased statistical approach for analyzing the effects of longitudinal biomarkers on time-to-event outcomes, providing an alternative to time-varying Cox proportional-hazards regression and the two-stage approach. However, whether available implementations of these methods perform reliably across different practically relevant scenarios remains insufficiently studied. We conducted a simulation study based on the Berlin Initiative Study examining kidney function and survival in older adults. In a manner comparable to phase IV studies in clinical research, our evaluation aims to provide insights into the practical performance of commonly used R package implementations of these methods, mostly under their default settings. By varying data generating scenarios, we assessed how different numbers of events and longitudinal measurements affect performance of Bayesian (JMbayes2) and frequentist joint model implementations (JM and joineRML), time-varying Cox PH regression (survival), and the two-stage approach (nlme and survival), focusing on bias in parameter estimates. Results revealed substantial variability across implementations. The JM package exhibited considerable bias and frequent convergence issues. In contrast, joineRML performed robustly with approximately unbiased estimates for association parameters and high convergence frequencies comparable to the implementations of the simpler methods across diverse scenarios. However, both frequentist packages systematically underestimated the effects of baseline covariates in the survival model. The Bayesian JMbayes2 was largely unbiased, but performance deteriorated under two conditions: with few events (< 70), convergence was low and bias persisted even in converged models; and with observation-to-event ratios below 2, convergence declined, although estimates from converged models remained approximately unbiased. Time-varying Cox PH regression and the two-stage approach showed more bias than JMbayes2 in certain settings but tended to achieve more robust performance and convergence across most scenarios.

**Supplementary Information:**

The online version contains supplementary material available at 10.1186/s12874-026-02875-4.

## Introduction

In medical research, understanding the longitudinal trajectories of biomarkers and their association with events (e.g. hospitalization, cardiovascular events, or death) is crucial. Several biostatistical tools have been suggested to appropriately model this association, where joint models for longitudinal and time-to-event data have emerged as a powerful tool for this purpose [1]. To ensure that available software implementations of these methods perform reliably across different settings, further investigations, such as simulation studies, are essential. Such studies provide valuable insights not only into the comparative strengths and limitations of different modelling approaches, but also into how their specific software implementations behave in practice, analogous to phase IV studies in clinical research [2]. Although joint models are often considered the “state of the art” for analyzing this type of data [3], the practical performance of their available R package implementations requires careful empirical evaluation. Accordingly, this study aims to compare commonly used R package implementations of joint models with implementations of two simpler approaches: time-varying Cox proportional-hazards (PH) regression models, and a two-stage approach.

Joint models offer two key advantages: (i) they quantify the impact of longitudinal biomarker changes on the risk of an event, such as death, and (ii) they address informative dropouts, where missing data is not random but associated with the unobserved biomarker values and event occurrence [3]. Joint models usually use a linear mixed-effects model for biomarker trajectories and a time-varying Cox PH model for the time-to-event data, linking them through shared random effects. This approach acknowledges potential measurement error and the discrete nature of biomarker measurements and handles missing not at random (MNAR) mechanisms more effectively than traditional methods [4]. This advantage stems from the incorporation of shared random effects, capturing the dependence between the missingness process and the longitudinal outcome variable, effectively mitigating bias that would otherwise arise due to MNAR. Bayesian approaches are gaining popularity [5] due to their flexibility in modelling the random effects structure [6, 7]. The joint model framework offers multiple ways to parameterize the association between longitudinal trajectory and survival process, e.g. using the current value, the current slope, or the area under the curve [4].

Cox PH regression models [8] with time-varying covariates [9] are particularly suited for investigating the influence of dynamic markers on event occurrence. Their key advantage over standard Cox PH models lies in the ability to incorporate covariates that change over time. Their key limitation though is their reliance on observed values, i.e. ignoring potential measurement error. Additionally, as values are only observed intermittently, these models usually assume a step function for time-varying covariates, implying that the levels of the covariates remain constant between measurement time points, which is often not realistic and can introduce substantial bias [10].

Unlike joint models, the two-stage approach models the longitudinal and survival processes separately [11]. Usually, a linear mixed-effects model is first fitted to the trajectory of the biomarker, and individual predictions at each observed event time are then used in a time-varying Cox PH regression. The main advantage of this approach is its simplicity, allowing for easier implementation of new methods and faster computation than with joint models [12]. However, a key limitation of separate modelling is its failure to fully use the available data, particularly regarding informative dropout. This omission can lead to biased estimates, especially in the initial model estimating the biomarker trajectory [12, 13]. Treating missingness as independent ignores informative missingness mechanisms (e.g., MNAR). However, in the last years several adjusted two-stage approaches were proposed that correct for this bias [14–18]. Another limitation is that plugging in predicted values from the first stage without accounting for their estimation uncertainty leads to underestimated standard errors and under-coverage of confidence intervals in the survival model [19, 20].

Comprehensive reviews of joint modelling frameworks, their methodological foundations, and available software implementations have been provided by Hickey et al. [21] and Papageorgiou et al. [22]. While these reviews describe the range of modelling choices, they do not systematically evaluate how specific R implementations perform under data-sparse conditions commonly encountered in practice.

Several studies have compared the three methods presented above. For example, simulation studies by Alvares and Leiva-Yamaguchi [16], Barrett et al. [23], Desmée et al. [12], Leiva-Yamaguchi and Alvares [15], Sweeting and Thomson [20], and Wu et al. [13] have shown that the uncorrected two-stage approach and time-varying Cox PH regression generally lead to biased estimates, while joint models remain unbiased. In general, simpler non-joint model methods tend to underestimate the effect of the longitudinal biomarker on the event [10, 24]. Moreover, the two-stage approach often leads to biased estimates in the longitudinal model due to unaccounted informative dropout, especially when the biomarker strongly affects event probability [12, 13]. Stegherr et al. [19] compared Bayesian joint models (R package JMbayes), Cox PH regression (survival), and the uncorrected two-stage approach (nlme and survival) for left-truncated data and found both joint models and the two-stage approach to be unbiased, with the latter showing better coverage probabilities. Cox PH regression, however, was biased, particularly in estimating the association between biomarker and event. Gonçalves et al. [25] compared the two-stage and the joint modeling approach using Monolix on a large database, investigating the longitudinal effect of tumor size on overall survival. Both methods provided similar overall results and adequately captured the observed survival distribution, though the two-stage approach yielded smaller estimates of the tumor growth effect on overall survival compared to the joint model.

Although joint models are well studied theoretically, empirical evaluations of how their implementations perform across a wide range of practically relevant scenarios remain scarce. Arisido et al. [26] found that joint models (R package JM) generally lead to unbiased estimates compared to time-varying Cox PH regression (survival). However, joint models can be substantially biased if either the longitudinal submodel or the baseline hazard in the survival submodel are misspecified, such as when non-linear biomarker trajectories are not appropriately modelled [6, 7]. In contrast, time-varying Cox PH regression is unaffected by these issues, as it does not require such specifications. Furthermore, Arisido et al. [26] observed that joint models exhibit greater bias when the biomarker remains nearly constant over time, i.e. when measurement error is small. Savel et al. [27] analyzed tumor growth trajectories in murine preclinical trials with different dropout mechanisms (MAR and MNAR) and found that joint models (JM) were biased under MAR dropout, whereas a mixed-effects model (lme4) remained robust in both scenarios.

In this study, we aim to enhance the understanding of joint model implementations by comparing the practical performance of commonly used R packages: JMbayes2 for Bayesian and JM and joineRML for frequentist joint models, alongside the survival and nlme packages for time-varying Cox PH regression, and the two-stage approach. Our primary focus is on the bias in effect estimates to quantify accuracy in parameter estimates. We focus on data-scarce situations commonly encountered in medical practice – settings where researchers may face practical difficulties employing joint models stably despite their theoretical superiority. By identifying when specific implementations perform well and highlighting their potential limitations in challenging scenarios, we aim to close an important gap in the literature and provide guidance for applied researchers.

The simulation study is based on real-world data from the Berlin Initiative Study (BIS) [28], enabling exploration of realistic scenarios while maintaining control over key parameters. By systematically varying aspects of the data-generating process, such as the density of longitudinal biomarker measurements and the strength of association between longitudinal and survival processes, we assess performance not only in settings similar to our empirical application, but also in more challenging scenarios. These include scenarios commonly encountered in practical applications, such as a limited number of events, which is a scenario particularly relevant for rare outcomes, or sparse longitudinal data, e.g. due to early events or dropouts. In our experience with longitudinal studies, such data limitations often constrain statistical analysis. Although our simulation is based on one specific study design, the data characteristics are broadly representative and provide relevant insights for researchers facing similar constraints in real-world applications.

## Methods

We consider the following scenario: In a study with $\:n$ subjects, we observe for each subject $i,\:i\:=\:1,\:\dots\,\:n$, a follow-up time ${T}_{i}=\mathrm{min}\left({T}_{i}^{*},{C}_{i}\right)$ where ${T}_{i}$ is either the event time $\:{T}_{i}^{*}$ or the censoring time ${C}_{i}$; and an event indicator ${{\updelta}}_{i}=I\left({T}_{i}^*<{C}_{i}\right)$. Let $\:{y}_{i}\left(t\right)$ denote the observed longitudinal biomarker for subject $i$ at time point $t$. The biomarker values are only observed intermittently such that for each subject $i$ we have a set of biomarker observations $\:{\boldsymbol{y}}_{ij}={\{y}_{i}\left({t}_{ij}\right),j=1,\dots,{n}_{i}\}$. These values potentially include some measurement error $\epsilon_{{t}_{ij}}\sim\mathcal{N}\left(0,{\sigma}^{2}\right)$ in addition to the true biomarker value ${m}_{i}\left({t}_{ij}\right),{y}_{i}\left({t}_{ij}\right)={m}_{i}\left({t}_{ij}\right)+\epsilon_{{t}_{ij}}.$ Our aim is to estimate the association between the biomarker and the hazard of the event (e.g. death). In the following we introduce three methods of increasing complexity which can be used to model this association, Table 1 summarizes the comparison of these methods.                        

### Time-varying Cox PH regression

One of the simplest methods to model the effect of a longitudinal biomarker on an event probability is the time-varying Cox PH regression [9]. It extends the Cox PH regression model [8] to include time-varying covariates, yielding the following hazard function for subject $\:i$ at time point $\:t$:

$$\:{h}_{i}\left(t\right)={h}_{0}\left(t\right)\mathrm{exp}\{{\boldsymbol{w}}_{i}^{\top}\boldsymbol{\upgamma\:}+{\upalpha\:}{y}_{i}\left(t\right)\},t>0$$

    

with $\:{h}_{0}$ denoting the baseline hazard, $\:{\boldsymbol{w}}_{i}$ representing a set of baseline survival covariates with linear effects $\:\boldsymbol{\upgamma\:}$, and $\:{\upalpha\:}$ reflecting the association between observed biomarker and event. Estimation of the parameters requires a biomarker value at each observed event time point $\:{T}_{i}^{*}$. However, since biomarker values are usually only available at certain time points, the time-varying Cox PH regression is typically used with the assumption that the biomarker remains constant between measurements, effectively following a step function. Additionally, the observed values $\:{y}_{i}\left({t}_{ij}\right)$ are used, such that potential measurement error in the observations is not accounted for.

### Two-stage approach

The two-stage approach [11] first models the longitudinal process in the biomarker, avoiding the strong assumption of the step function and accounting for potential measurement error. The simplest version involves two steps:

1. Fitting a model for the biomarker’s longitudinal trajectory (e.g., a linear mixed-effects model).
2. Using the predicted biomarker values from the longitudinal model at each event time in a time-varying Cox PH regression model.

Modelling the biomarker in the first step allows for extrapolation of biomarker values for individuals still at risk at each event time for the second step. However, failing to account for potential informative dropouts (MNAR) in the first step can lead to biased estimates. Several adjusted two-stage approaches were proposed that correct for this bias [14–18]. Mauff et al. [14] for example propose to first fit a (multivariate) mixed-effects model for the longitudinal outcome(s), and then in the second stage, to update both the survival parameters and the random effects while applying importance sampling weights to correct for bias (implemented in the R package JMbayes [5]). In the following we will always refer to the uncorrected two-stage approach if not stated differently.

Note also that plugging the estimates from the first step into the survival model does not account for the uncertainty in their estimation, resulting in underestimated standard errors and under-coverage of confidence intervals in the survival model [20], whereby the true parameter value falls outside the stated confidence interval more often than indicated by the nominal confidence level. Methods such as bootstrapping (frequentist) or using the available Markov-Chain-Monte-Carlo (MCMC) samples from the first stage estimation in the second stage (Bayesian) [14] can though address the underestimation of the standard errors in the survival submodel.

### Joint model

A joint model [4] estimates all model parameters simultaneously by deriving a joint likelihood for both the longitudinal and the survival process. This is based on the assumption of conditional independence between the two submodels and the repeated observations, given the random effects, that is.

$$\begin{array}{l}\:p\left({T}_{i},{{\updelta}}_{i},{\boldsymbol{y}}_{i}\mid{\boldsymbol{b}}_{i};\boldsymbol{\upvartheta}\right)=p\left({T}_{i},{{\updelta}}_{i}\mid{\boldsymbol{b}}_{i};\boldsymbol{\upvartheta}\right)\cdot{p}\left({\boldsymbol{y}}_{i}\mid{\boldsymbol{b}}_{i};\boldsymbol{\upvartheta}\right),\;\mathrm{and}\hspace{1em}\\\:p\left({\boldsymbol{y}}_{i}\mid{\boldsymbol{b}}_{i};\boldsymbol{\upvartheta}\right)={\prod}_{j}p\{{\boldsymbol{y}}_{i}\left({t}_{ij}\right)\mid{\boldsymbol{b}}_{i};\boldsymbol{\upvartheta}\}\end{array}$$

where $\boldsymbol{\upvartheta}=\left[{\boldsymbol{\upvartheta}}_{t}^{\top},{\boldsymbol{\upvartheta}}_{y}^{\top},{\boldsymbol{\upvartheta}}_{b}^{\top}\right]$ represents the full parameter vector of the model including the parameters of the survival submodel $\:{\boldsymbol{\upvartheta}}_{t}^{\top}$, the longitudinal submodel $\:{\boldsymbol{\upvartheta}}_{y}^{\top}$, and the parameters $\:{\boldsymbol{\upvartheta}}_{b}^{\top}$ for the random effects’ covariance matrix. Given this assumption of independence, the joint likelihood follows as

$$\:L\left(\boldsymbol{\upvartheta}\mid\boldsymbol{T},\boldsymbol{\updelta},\boldsymbol{y}\right)={\prod}_{i=1}^{n}p\left({T}_{i},{{\updelta}}_{i},{\boldsymbol{y}}_{i}\mid\boldsymbol{\upvartheta}\right)$$

$$\:={\prod}_{i=1}^{n}\int\:p\left({T}_{i},{{\updelta}}_{i},{\boldsymbol{y}}_{i}\mid{\boldsymbol{b}}_{i};\boldsymbol{\upvartheta}\right)\cdot{p}\left({\boldsymbol{b}}_{i};{\boldsymbol{\upvartheta}}_{b}\right)d{\boldsymbol{b}}_{i}$$

$$\:={\prod}_{i=1}^{n}\int\:p\left({T}_{i},{{\updelta}}_{i}\mid{\boldsymbol{b}}_{i};{\boldsymbol{\upvartheta}}_{t}\right)\hspace{0.17em}\left[{\prod}_{j=1}^{{n}_{i}}p\left({\boldsymbol{y}}_{i}\left({t}_{ij}\right)\mid{\boldsymbol{b}}_{i};{\boldsymbol{\upvartheta}}_{y}\right)\right]\cdot{p}\left({\boldsymbol{b}}_{i};{\boldsymbol{\upvartheta}}_{b}\right)d{\boldsymbol{b}}_{i}$$

To obtain parameter estimates, an Expectation Maximization (EM) algorithm is often used to optimize the likelihood [4].

The frequentist joint models presented so far require integration over the random effects’ distribution, which can be numerically challenging, especially with complex random effects structures [6]. This can lead to convergence issues or unrealistic estimates. Alternatively, a Bayesian approach treats random effects as additional parameters with priors, eliminating the need for integration in the likelihood formulation. The associated posterior distribution [5] then is

$$\:p\left(\boldsymbol{\upvartheta},\boldsymbol{b}\mid\boldsymbol{y},\boldsymbol{T},\boldsymbol{\updelta}\right)\propto\:\prod\limits_{i=1}^{n}p\left({T}_{i},{{\updelta}}_{i}\mid{\boldsymbol{b}}_{i},{\boldsymbol{\upvartheta}}_{t}\right)[\prod\limits_{j=1}^{{n}_{i}}p\left({y}_{ij}\mid{\boldsymbol{b}}_{i};{\boldsymbol{\upvartheta}}_{y}\right)]\cdot{p}\left({\boldsymbol{b}}_{i};{\boldsymbol{\upvartheta}}_{b}\right)p\left(\boldsymbol{\upvartheta}\right)$$

where $\:{\boldsymbol{\upvartheta}}_{t}$, $\:{\boldsymbol{\upvartheta\:}}_{y}$, and $\:{\boldsymbol{\upvartheta\:}}_{b}$ are random variables with their associated joint prior distribution $p\left(\boldsymbol{\upvartheta}\right).$  

### Comparison of the three methods

Table 1 gives an overview of the advantages and limitations of the different methods. As outlined above, only joint models appropriately handle measurement error in the biomarker, informative dropout, and acknowledge the uncertainty of the biomarker measurement accordingly in the time to event model. The observation-to-event ratio (last column Table 1), defined as the total number of longitudinal observations divided by the total number of events in a simulated dataset, serves as a summary measure of data density. It is not an experimental factor but a derived quantity, reflecting the interplay of multiple data-generating parameters: observation density, event timing (influenced by the association parameter $\alpha\:$ and baseline hazard $\phi$), and censoring. In our simulation design, we systematically varied the underlying data-generating parameters, the observation-to-event ratio serves as a convenient descriptor of the resulting data structure for comparisons across scenarios.    

**Table 1:** Overview of advantages and limitations of time-varying Cox proportional-hazards regression, uncorrected two-stage approach, and joint model

Challenges	Time-varying Cox proportional-hazards regression	Uncorrected two-stage approach	Joint Model
Measurement error in biomarker	✖	✔	✔
Informative dropout (MNAR) in longitudinal model	✖	✖	✔
Explicit modeling of biomarker trajectory	✖	✔	✔
Low computation time	✔	✔	✔/✖
Acknowledgement of uncertainty in biomarker for standard errors in time to event model	✖	✖	✔

✖ not handled appropriately / not accounted for

✔ handled appropiately

### Simulation study

To compare the practical performance of R package implementations of the methods presented above, we conducted a simulation study based on data from the Berlin Initiative Study (BIS), a cohort study of older adults in Berlin [28] including $\:n\:=\:2069$ individuals with on average 3.4 longitudinal biomarker observations. The BIS focuses on kidney function in individuals over 70, with one research question of interest being the association between kidney function and survival. Kidney function is measured longitudinally with follow-up visits every two years using the estimated glomerular filtration rate (eGFR). The study here serves as a typical medical study facing limited number of longitudinal observations and follow-up due to dropouts and deaths. Our interest lies in systematically evaluating the effects of these data limitations on the relative performance of the discussed approaches.

### Data generation

We first fit a frequentist joint model to the original data using the R package JM [29]. To address convergence issues of the JM package, time is expressed in weeks instead of years or months.

We consider two different specifications for the longitudinal biomarker trajectory to evaluate model performance under varying degrees of complexity:

Linear trajectory (Settings 1a-5a): A linear trajectory with random intercepts and slopes is specified for the longitudinal submodel, reflecting the original BIS data quite well. The submodel for the biomarker depending on time $\:t$ is defined as follows:

$$\:{log(eGFR}_{i})\left(t\right)=\:{\beta}_{0}+{\beta}_{1}\cdot{t}+\:{\beta}_{2}\cdot{age}_{i}+{b}_{0i}+{b}_{1i}\cdot{t}+\epsilon_{it}$$

$$\:={m}_{i}\left(t\right)+{\epsilon}_{it}$$

where $\:\boldsymbol{\beta}=\left[{\beta}_{0},{\beta}_{1},{\beta}_{2}\right]$ represents the fixed effects, $\:\boldsymbol{b}\sim\mathcal{N}\left(0,\boldsymbol{D}\right)$ captures subject-specific random intercepts and slopes with a 2 × 2 covariance matrix $\boldsymbol{D}$, $\:\epsilon_{it}\sim\mathcal{N}\left(0,{\sigma}^{2}\right)$ is the measurement error with standard deviation $\:\sigma$, and $\:{m}_{i}\left(t\right)$ is the true unobserved biomarker value at time point $\:t$ for subject $\:i$.

Nonlinear trajectory (Settings 1b – 3b): To assess model behaviour in a nonlinear setting, we additionally simulated data using cubic B-splines with three basis functions, where the coefficients $\:{\boldsymbol{\beta}}_{1}=[{\beta}_{11},{\beta}_{12},{\beta\:}_{13}]$ for the basis functions were chosen to create a nonlinear trajectory.

The survival submodel for the time-to-event outcome combines the model for the biomarker with other independent variables to model the hazard h(t) of the event using the following model:

$${h}_{i}\left(t\right)={h}_{0}\left(t\right)\cdot\mathrm{exp}\left({\gamma}_{1}\cdot{age}_{i}+{\gamma}_{2}\cdot{sex}_{i}+\alpha\cdot{m}_{i}\left(t\right)\right),$$

where for simplicity a Weibull baseline hazard ${h}_{0}\left(t\right)=\phi\:{t}^{\phi\:-1}$ is assumed which approximately reflects the baseline hazard observed in the original data, and $\:\alpha\:$ describes the relationship between biomarker and event. Throughout this work, we use the current value parametrization for the relationship between biomarker and log-hazard, one of the most commonly used parametrizations.

Based on estimates from the model fitted to the original BIS data, we generate 200 datasets for each simulation scenario. Each dataset consists of $n\:=\:200$ subjects with observation time points at $\boldsymbol{t}\:=\{0,\:100,\:200,\:300,\:400\}$ weeks, approximating measurements every two years similar to the original study, unless stated otherwise. The number of subjects was chosen to be smaller than in the original data as this decreased the running times for the simulation. Increasing the number of subjects though did not substantially change the simulation results. For each subject $\:i$, we sample from $age_{i}\sim\:\mathcal{N}\left(80,\:6.7\right)$ and $\:se{x}_{i}\sim\:Bernoulli\left(0.52\right)$, reflecting the distribution of the BIS data. Measurement errors are drawn from ${\epsilon}_{it}\sim\:\mathcal{N}\left(0,\:{\widehat{\sigma}}^{2}\right)$, random intercept and slope from $\:{\boldsymbol{b}}_{i}\sim\:\mathcal{N}\left(0,\widehat{\boldsymbol{D}}\right)$, using the estimates from the JM fit to the data ($$\widehat{\sigma\:}=0.092;\:\widehat{\boldsymbol{D}}=\:\left(\begin{array}{cc}0.05&\:2e-5\\\:2e-5&\:1.6e-7\end{array}\right)$$), which are then used to generate the longitudinal biomarker observations $\:{y}_{i}\left(t\right)$. The remaining parameter estimates from the frequentist JM fit used to simulate the data are shown in Table 3. Event times $\:{T}_{i}^{*}$ are simulated using the inversion method of Crowther and Lambert [30], with censoring being introduced through $\:{C}_{i}={min}({\stackrel{\sim}{C}}_{i},400)$ with $\:{\stackrel{\sim}{C}}_{i}\mathcal{\:}\sim\mathcal{\:}\mathcal{U}\left(0,\:800\right)$, such that the observed event time is $\:{T}_{i}=min\left({C}_{i},{T}_{i}^{*}\right)$.

We use the following R packages for comparison: JM [29] (version 1.5-2) and joineRML [31] (version 0.4.6) for frequentist joint models; JMbayes2 (version 0.8–85) for Bayesian joint model [32]; nlme (version 3.1–162) [33] and survival (version 3.5-7) [34] for the two-stage approach implementation; and survival (version 3.5-7) [34] for the time-varying Cox PH regression. All functions are applied with their default settings if not stated differently. In the lme function (R package nlme), we set the optimizer to optim instead of the default nlminb, to reduce convergence issues in the estimation of the JM package and the two-stage approach. For all approaches, the true longitudinal and hazard models are assumed to ensure comparability. joineRML showed numerical difficulties when the censoring time point equals the last observation time point. In those cases, we have added 0.001 (~ 10 min) to the observed event time. Given the time scale of our study, this adjustment is negligible and purely technical. Additionally, for Setting 2 as introduced below, we include the corrected two-stage approach proposed by Mauff et al. [14] implemented in JMbayes [5] (version 0.8–85). Deviating from the default settings we used the set.seed function before the function call to JAGS to circumvent an error in the package (as the function jags does no longer take a seed argument). The R code used for the simulations is available at https://github.com/jilhee/jm_comparison.

It is important to note that while the data-generating mechanism uses a Weibull baseline hazard, the model fitting procedures differ across methods: the JM package assumes a Weibull baseline hazard in the model specification, ensuring correct specification; joineRML, time-varying Cox PH regression, and two-stage approach leave the baseline hazard unspecified. JMbayes2, by contrast, uses its default baseline hazard specification with penalized B-splines, as a Weibull parameterization is not implemented, introducing a mild misspecification by fitting a flexible spline approximation to data generated from a parametric Weibull distribution.

Furthermore, the implementations differ in how they parametrize the association between the longitudinal and the survival process. JM, JMbayes2, and the two-stage approach use a current value parametrization, which directly corresponds to our data-generating mechanism. joineRML, by contrast, parametrizes the association through the shared random effects rather than through the current value of the longitudinal trajectory. While both parametrizations can capture the dependence between the longitudinal and the survival process, they correspond to different model specifications, and the joineRML parametrization does not directly match the data-generating mechanism used in our simulations.

For JMbayes2 we deviated from the default settings and used 40,000 MCMC iterations plus a burn-in period of 5,000 and thinning of 20, resulting in 2,000 posterior samples per chain across three parallel chains. Convergence was assessed via effective sample sizes (ESS) and Rhat, requiring ESS > 1,000 and Rhat < 1.05 for the association parameter. In the frequentist joint model packages, convergence was evaluated using the positive-definiteness of the Hessian matrix and software-provided convergence indicator.

Our comparison reflects the performance of specific software implementations rather than inherent differences between frequentist and Bayesian statistical frameworks. Different implementations of the same methodological approach may yield different results due to variations in numerical algorithms, integral approximation techniques, and default optimization settings [35].

We designed five different simulation scenarios that vary a single parameter each (Settings 1–5), where settings a always simulate a linear biomarker trajectory and settings b a nonlinear one. Note that changing one parameter in isolation is not possible without also affecting other parameters. For instance, altering the association parameter $\:\alpha\:$ modifies timing and frequency of events $\:{\boldsymbol{T}}^{*}$, thereby influencing the number of longitudinal observations. Table 2 illustrates the five simulation scenarios and their effects on event and observation times.

**Table 2:** Five simulation settings: Association between variation in one single parameter and the effect on the data generating process

				Effect of change on			
**Varied parameter**	**Value(s) in original joint model fit**	**Simulated range**	**Change**	**Number of events**	**Time point of events**	**Number of longitudinal observations**	**Observation-to-event ratio**
Density of biomarker observations	[0, 100, 200, 300, 400]	Every 20th to 300th week	n_i_ ↑ for y_i_ ={y_i_(t_ij_), j = 1, …, n_i_}	no change	no change	↑	↑
Association parameter α	-1.07	-1.5 to 0	α → 0	*↑*	earlier	↓	↓
Baseline hazard parameter φ	1.97	1.6 to 2.8	φ ↑	↑	earlier	↓	↓
Time effect β_1_	-0.0007	-0.05 to 0	β_1_ → 0	↓	later	↑	↑
SD measurement error$\:\sigma\:$	0.092	0.05–0.5	$\:\sigma\:$↑	no change	no change	no change	no change

Comparing the different software implementations, our primary focus is on the performance of the estimation of the association parameter $\:\alpha\:$. To assess the relevance and magnitude of the bias in this parameter, we present the median parameter estimate in comparison to the true simulated effect (indicated by a red horizontal line) on the y axis. The x axis displays the varied simulation parameter. Variability in the estimated effects for each implementation is visualized using boxplots, ideally based on 200 models per method and simulation setting. Primary analyses report bias conditional on model convergence, as non-converged models do not produce reliable parameter estimates. To avoid obscuring the full extent of methodological challenges, we also report convergence frequencies. Additionally, results for a given approach are displayed only for settings with at least 50 out of 200 converged models. Unconditional results (including all models regardless of convergence) as well as results for settings where all models converged are provided in the Supplement.

## Results

For clarity, the following section focuses solely on $\alpha$, representing the effect of the longitudinal biomarker on the event. Estimates for the other parameters are provided in the appendix (Supplementary Figs. 1–20). Regarding the nonlinear simulation settings, we present only the results from Setting 1b. The results for the remaining nonlinear settings (Settings 2b and 3b) are provided in the Supplement (Supplementary Figs. 21 and 22), as they show similar patterns to their linear counterparts and do not reveal substantially different findings. Detailed mean computation times for the simulation settings are provided in the Supplement (Supplementary Table 1). Across all scenarios, the time-varying Cox PH model exhibits the shortest mean computation times (~ 0.1s), followed by the two-stage approach (0.1–0.5 s), the JM package (2.3–18.1 s), and joineRML (9.4–29.5 s), while the Bayesian implementation JMbayes2 has the longest computation times with 380.0 to 729.3 s. However, reported computation times, particularly for the Bayesian joint model, should be interpreted in the context of the specific MCMC settings; optimization of these settings, including e.g. prior specifications, was not an objective of this study.

### Setting 1a: density of biomarker observations (linear trajectory)

**Fig. 1:**
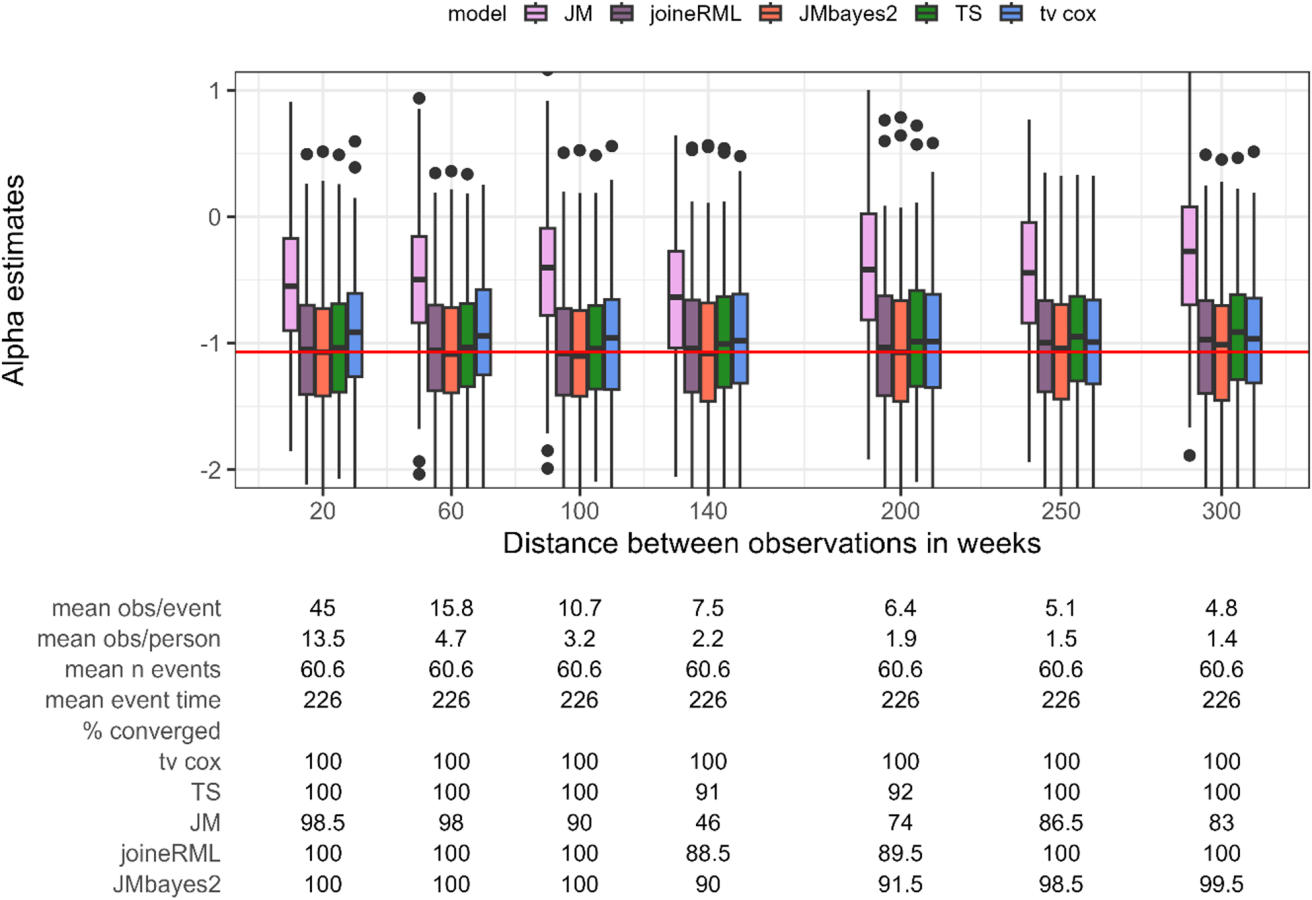
Estimates for the association parameter α (y axis) while varying the distance between longitudinal observations of the biomarker and thus their density (x axis). Red horizontal line indicates true parameter value. tv cox, time-varying Cox proportional-hazards regression; TS, two-stage approach; JM and joineRML, frequentist joint models; JMbayes2, Bayesian joint model. % converged, percentage of converged models out of 200 simulations; obs, number of observations; n event, number of events. Based on 200 simulations

To assess the impact of longitudinal data density, we varied the density of biomarker measurements, ranging from observations every 20 weeks (left in Fig. 1) to a maximum of only two measurements at baseline and week 300 (right in Fig. 1). The number and timing of events are unchanged across simulations. Across different follow-up densities, the bias in the association parameter $\alpha\:$ is relatively stable for all methods. The JM package (frequentist) exhibits the largest bias and frequent convergence issues, with only 46.0% to 98.5% of models converging depending on the scenario. In contrast, the Bayesian joint model (JMbayes2) and joineRML package (frequentist) show approximately unbiased estimates on average. The two-stage approach generally outperforms the time-varying Cox PH regression and is only somewhat worse than the Bayesian joint model. Further reducing the ratio of observations to events is not feasible (Fig. 1), since only longitudinal measurement time points are varied, while keeping the event times constant. JMbayes2, joineRML, two-stage approach, and time-varying Cox PH regression all demonstrate robustness even in more extreme scenarios where we simulate only approximately 1.5 biomarker observations per person on average, but only the time-varying Cox PH regression always converges.

### Setting 1b: density of biomarker observations (nonlinear trajectory)

In this setting, we assess the method performance with nonlinear biomarker trajectories while varying the density of longitudinal observations similar to the previous Setting 1a. The biomarker trajectory was modelled using cubic B-splines with three basis functions.

**Fig. 2:**
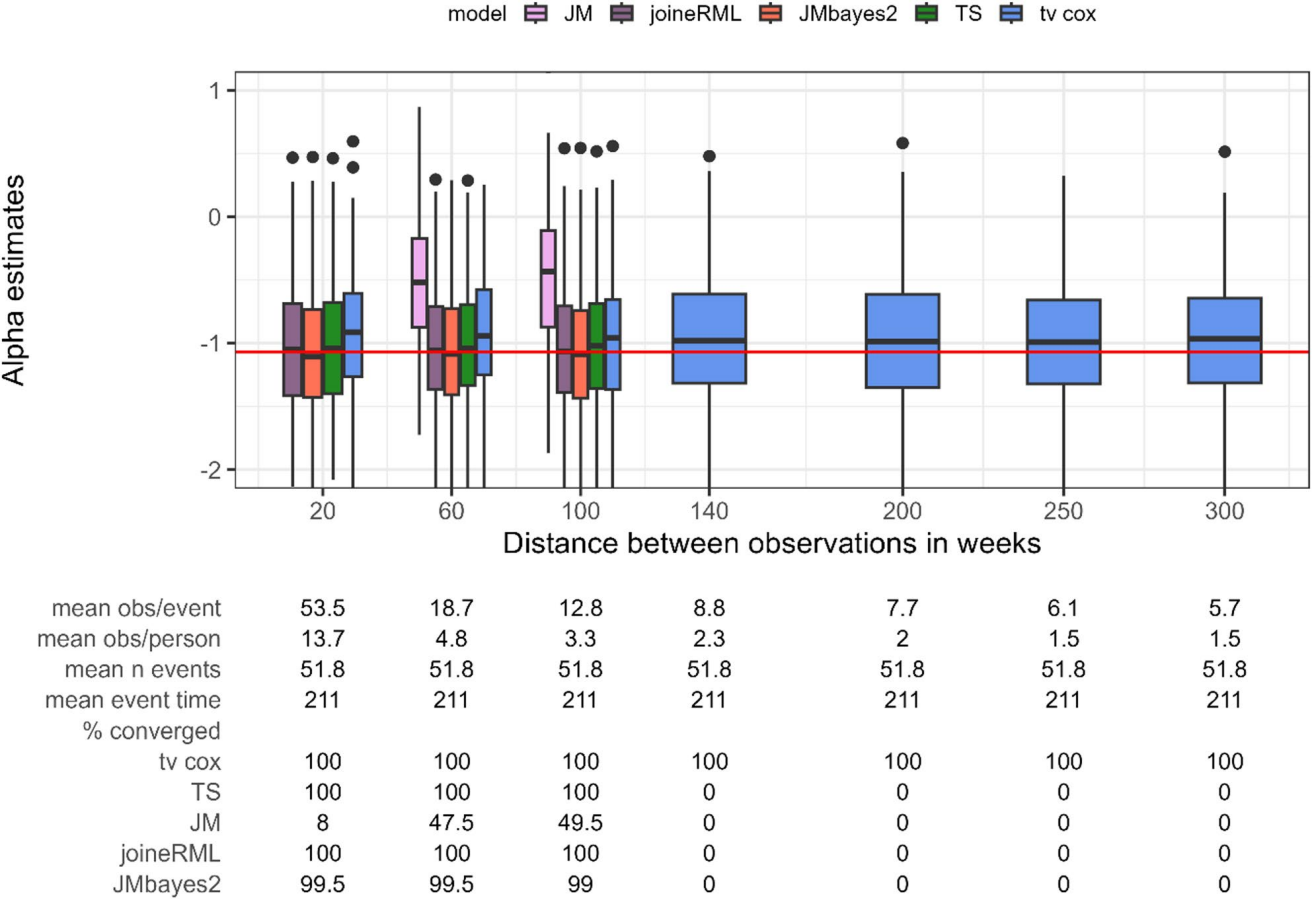
Estimates for the association parameter α (y axis) while varying the distance between longitudinal observations of the biomarker and thus their density (x axis) modelling nonlinear biomarker trajectories. Red horizontal line indicates true parameter value. tv cox, time-varying Cox proportional-hazards regression; TS, two-stage approach; JM and joineRML, frequentist joint models; JMbayes2, Bayesian joint model. % converged, percentage of converged models out of 200 simulations; obs, number of observations; n event, number of events. Based on 200 simulations

As expected, nonlinear trajectories require more frequent longitudinal observations to achieve convergence of the models (Fig. 2). The JM package (frequentist) still underestimates the association parameter with substantial bias and convergence issues throughout. JMbayes2, joineRML, two-stage approach, and time-varying Cox PH regression perform similarly to the linear setting when sufficient measurements are available (on average > 3 observations per person). Similar results are found for Settings 2b and 3b in Supplementary Figs. 21 and 22, respectively.

### Setting 2a: association parameter α 

**Fig. 3:**
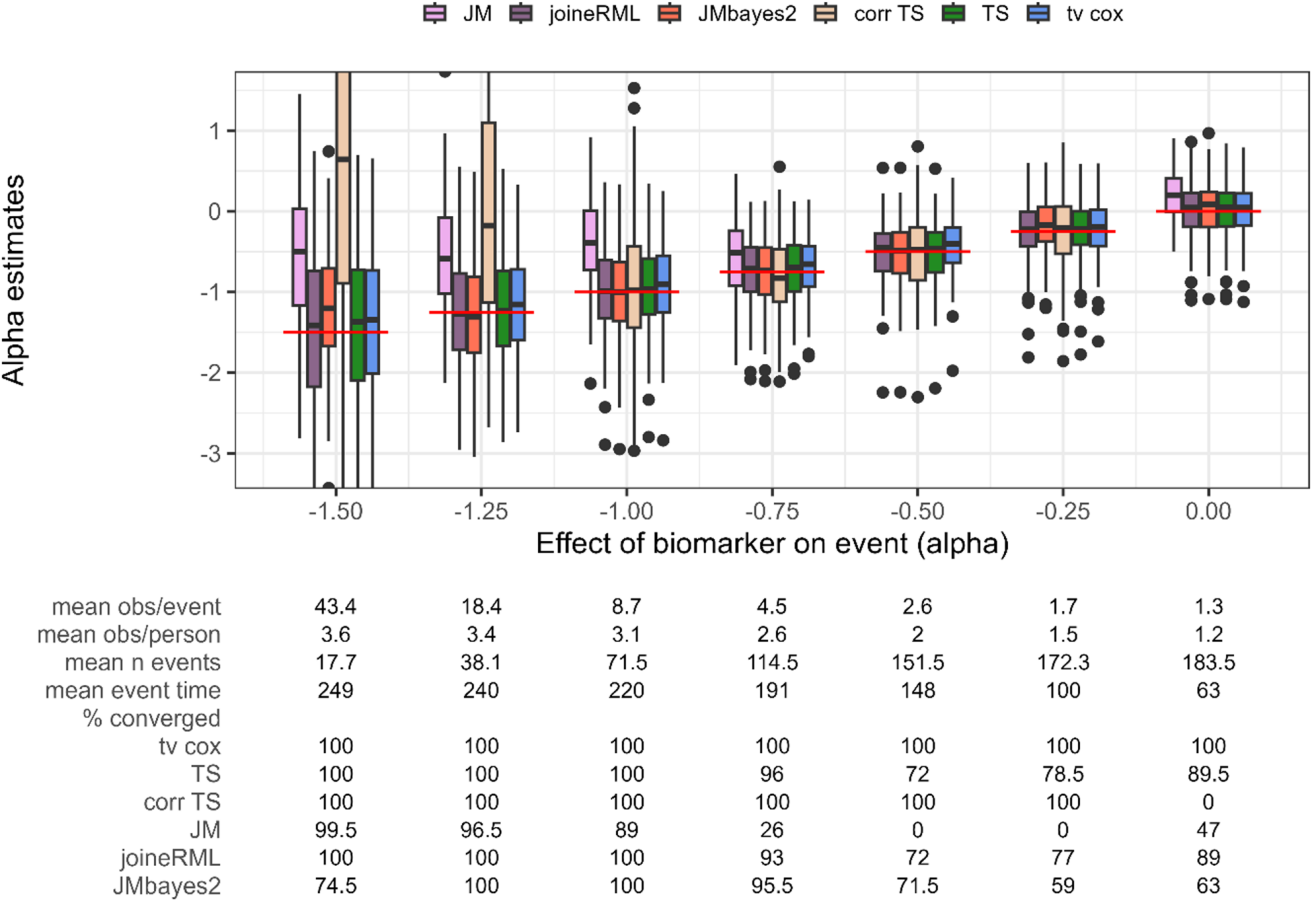
Estimates for the association parameter α (y axis) while varying the association parameter α (x axis). Red horizontal line indicates true parameter value. tv cox, time-varying Cox proportional-hazards regression; TS, two-stage approach; corr TS, corrected two-stage approach; JM and joineRML, frequentist joint models; JMbayes2, Bayesian joint model. % converged, percentage of converged models out of 200 simulations; obs, number of observations; n event, number of events. Based on 200 simulations

In Setting 2, we varied the true association parameter $\:\alpha\:$, evaluating different biomarker effect sizes on the risk of an early event. Since the biomarker has a protective effect here (i.e. negative values of $\:\alpha\:$), smaller absolute values of $\:\alpha\:$ (right in Fig. 3) result in earlier occurrence of events, a reduction in the number of longitudinal observations per person, and consequently, a lower observation-to-event ratio.

The JM package (frequentist) clearly underestimates the effect estimate across all scenarios and has convergence issues for smaller effect sizes (< 25.0% models converged, Fig. 3). JMbayes2 demonstrates varying performance across the range of $\:\alpha\:$ values. For larger absolute values of $\:\alpha\:$, resulting in very few events due to the protective biomarker effect, JMbayes2 shows increased bias and convergence issues (28.5% to 72.0% convergence). However, for moderate effect sizes ($\:\alpha\:$ between − 1.0 and − 0.5) with an increasing number of events (> 70 events), the Bayesian joint model exhibits minimal bias, smaller variability, and improved convergence frequencies up to 99.0%. As $\:\alpha\:$ approaches zero and the observation-to-event ratio further decreases, convergence declines again to 59.0-71.5%. Notably, the models that converge show minimal to no bias in parameter estimates, even under these challenging conditions.

joineRML, two-stage approach and time-varying Cox PH regression demonstrate robust performance across all scenarios, with minimal bias, with joineRML and two-stage approach showing slightly smaller bias. For scenarios with larger absolute $\:\alpha\:$ values, all three methods exhibit increased variability in parameter estimates, which diminishes as the number of events increases. Time-varying Cox PH regression consistently achieves 100.0% convergence, whereas joineRML and two-stage approach show very similar convergence frequencies between 72.0% and 100.0%, with lower frequencies observed at smaller observation-to-event ratios. We additionally compare results to the corrected two-stage approach proposed by Mauff et al. [14]. Contrary to expectations, the corrected method does not improve performance over the uncorrected approach. Instead, it exhibits increased bias, particularly for larger effect sizes, and shows convergence issues in extreme settings (0.0% convergence at $\:\alpha\:=0$).

Unlike Setting 1, where observation density varied while keeping the number of events constant, the increased frequency and earlier occurrence of events in this setting results in observation-to-event ratios dropping below two in several scenarios. Under these conditions, the Bayesian joint model becomes more prone to convergence issues, while joineRML, two-stage approach, and time-varying Cox PH regression remain robust. Across settings, the average number of longitudinal observations decreases to as low as 1.5 per person, highlighting the challenge of sparse data for joint model approaches.

### Setting 3a: baseline hazard parameter$\:\phi\:$

In Setting 3, the baseline hazard parameter $\:\phi\:$ is varied, where a larger $\:\phi\:$ results in earlier and therefore more events, thereby reducing the observation-to-event ratio.

**Fig. 4:**
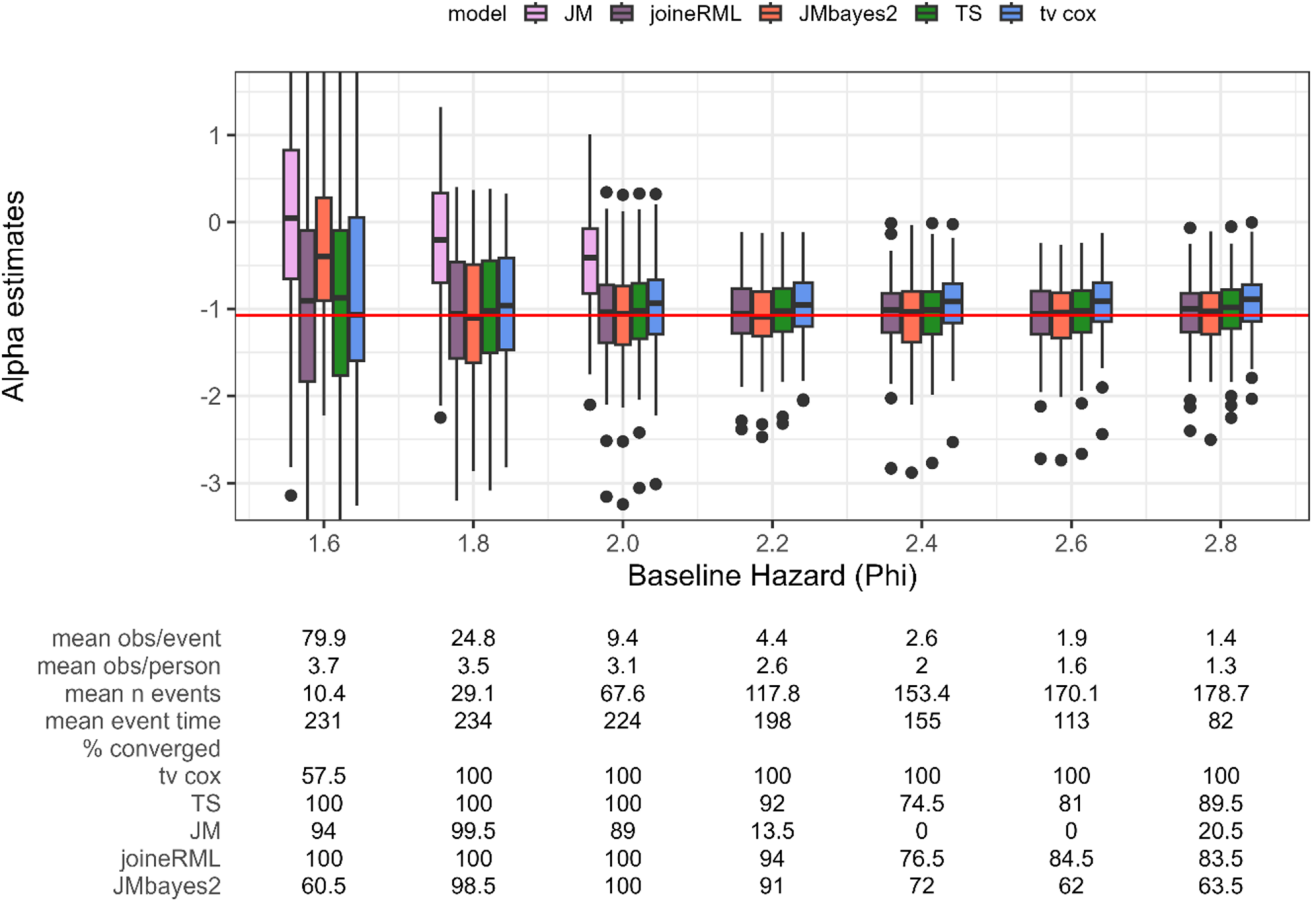
Estimates for the association parameter α (y axis) while varying the baseline hazard (x axis). Red horizontal line indicates true parameter value. tv cox, time-varying Cox proportional-hazards regression; TS, two-stage approach; JM and joineRML, frequentist joint models; JMbayes2, Bayesian joint model. % converged, percentage of converged models out of 200 simulations; obs, number of observations; n event, number of events. Based on 200 simulations

Similar to the previous settings, JM (frequentist) shows the largest bias in the association parameter $\:\alpha\:$ and convergence problems for smaller values of $\:\phi\:$ (Fig. 4). joineRML (frequentist) produces approximately unbiased parameter estimates across all scenarios. The Bayesian joint model (JMbayes2) shows performance patterns similar to Setting 2. For smaller $\:\phi\:$ values, it exhibits severe convergence issues with only 34.0–60.0% convergence and large bias, substantially underestimating the true parameter. As $\:\phi\:$ and consequently the number of events increases, bias decreases to nearly zero. However, when the observation-to-event ratio drops further at higher $\:\phi\:$ values, convergence problems re-emerge (62.5–71.5% convergence), though the models that converge show no increased bias. Both the two-stage approach and time-varying Cox PH regression remain fairly stable throughout all scenarios, with the two-stage approach generally slightly outperforming the time-varying Cox PH regression in terms of bias. Only the time-varying Cox PH regression always converges, whereas for joineRML and the two-stage approach the percentage of converged models gets as low as 74.5%. As in Setting 2, joineRML and two-stage approach show similar results in terms of bias and convergence frequencies.

Varying $\:\phi\:$ also implicitly alters the number of events. As the number of events increases, bias and variability decrease across all methods. However, for larger $\:\phi\:$ values, this positive effect is counterbalanced by earlier event occurrence, which reduces the number of longitudinal biomarker observations. This reduction in longitudinal data particularly affects the Bayesian joint model, leading to increased convergence issues. In contrast, the two-stage approach and time-varying Cox PH regression demonstrate lower sensitivity to this reduction in longitudinal observations, maintaining stable performance even when observation-to-event ratios become unfavourable.

### Setting 4a: time effect $\:{\beta}_{1}$ in biomarker

**Fig. 5:**
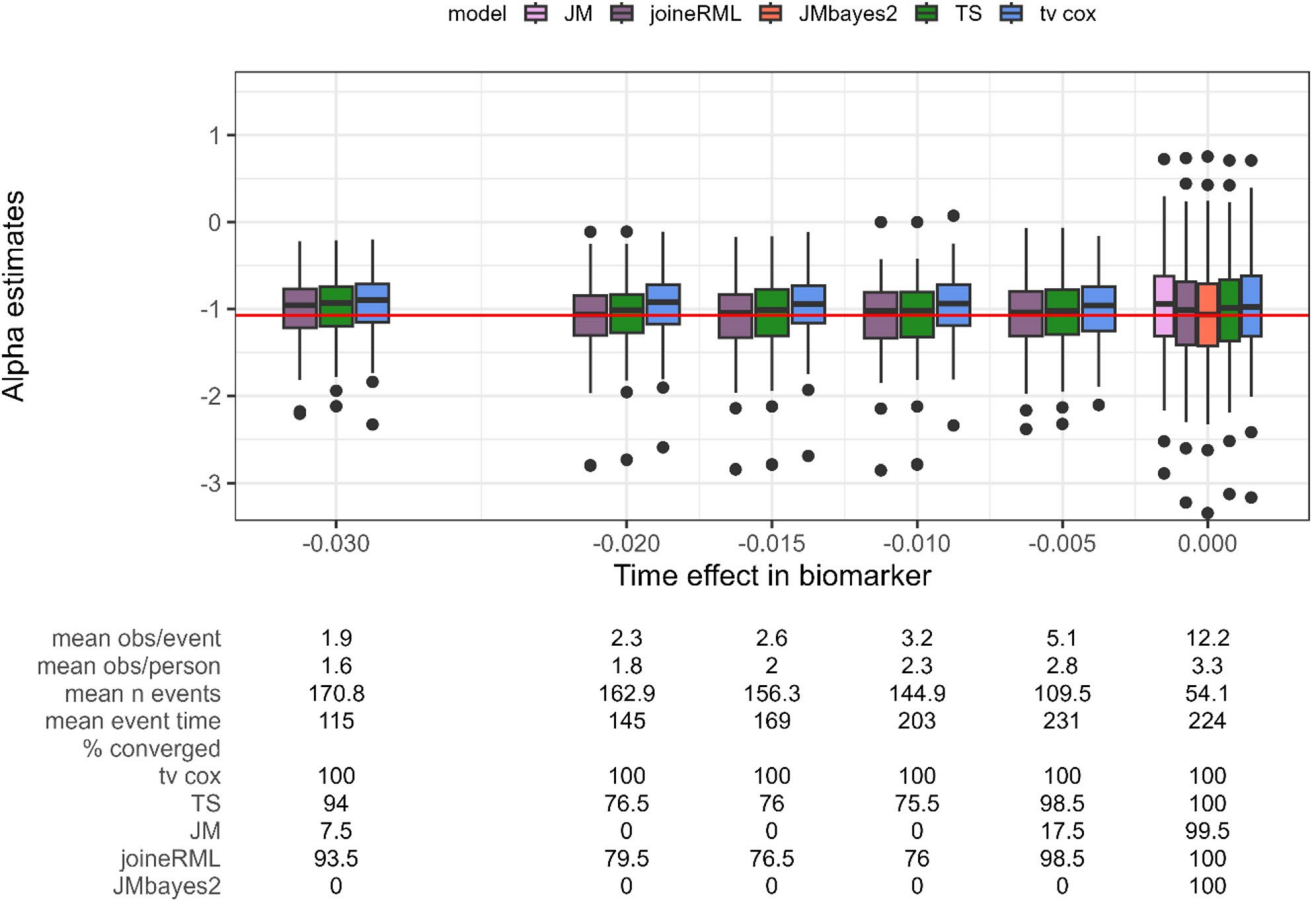
Estimates for the association parameter α (y axis) while varying the time effect in the longitudinal submodel (x axis). Red horizontal line indicates true parameter value. tv cox, time-varying Cox proportional-hazards regression; TS, two-stage approach; JM and joineRML, frequentist joint models; JMbayes2, Bayesian joint model. % converged, percentage of converged models out of 200 simulations; obs, number of observations; n event, number of events. Based on 200 simulations

In Setting 4, the time effect $\:{\beta}_{1}$ in the longitudinal model is varied - stronger time effects (left in Fig. 5) lead to earlier and more events, therefore reducing the observation-to-event ratio.

Both JMbayes2 and the JM (frequentist) show substantial convergence problems in this simulation scenario, converging only in the setting without time effect. In this single converged setting, JMbayes2 produces unbiased estimates, while JM underestimates the association parameter.

joineRML, two-stage approach and time-varying Cox PH regression perform similarly again, with joineRML performing the best and the two-stage approach being slightly less biased than time-varying Cox PH across all scenarios. Again, only the time-varying Cox PH regression always converges, with the percentage of converged models for joineRML and two-stage approach dropping as low as 76.0%. Convergence issues for the two-stage approach and joineRML typically arise already at the stage of fitting the longitudinal submodel (e.g., using the lme function), preventing subsequent estimation of the survival model. In JM (frequentist), warnings and errors are often related to the scaling of the time variable, to which this package appears particularly sensitive (as also observed in the model fit to the original data). Examination of unconditional results (Supplementary Fig. 29), which include all models that produced parameter estimates regardless of convergence status, shows that JMbayes2 produces approximately unbiased results even when models fail to meet convergence criteria (ESS ≥ 1,000, Rhat < 1.05). This suggests that in this particular simulation setting, the non-converged JMbayes2 runs, while showing inadequate MCMC mixing, on average still yield reasonably accurate point estimates.

### Setting 5a: increasing measurement error

**Fig. 6:**
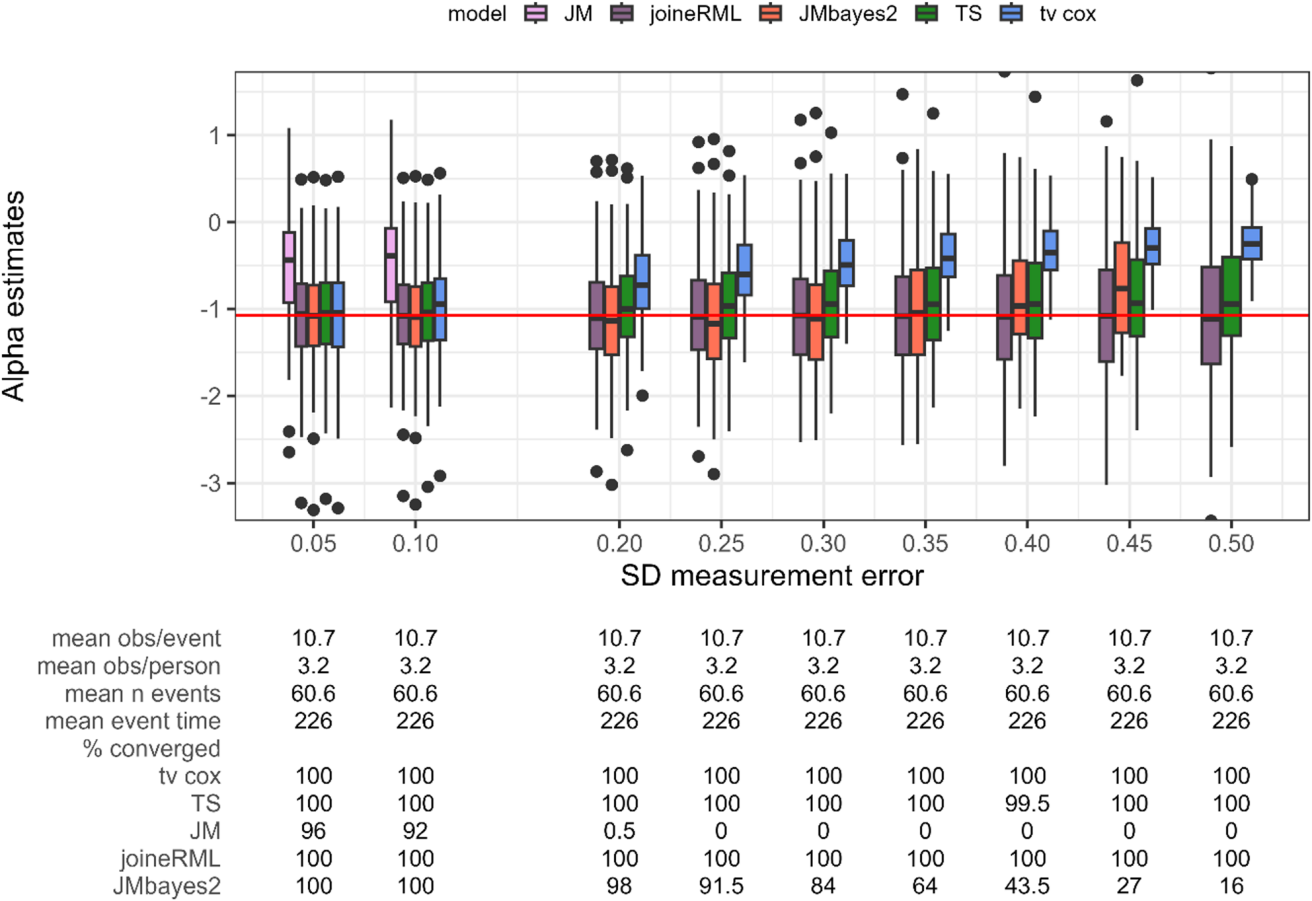
Estimates for the association parameter α (y axis) while varying the standard deviation of the measurement error (x axis). Red horizontal line indicates true parameter value. tv cox, time-varying Cox proportional-hazards regression; TS, two-stage approach; JM and joineRML, frequentist joint models; JMbayes2, Bayesian joint model. % converged, percentage of converged models out of 200 simulations; obs, number of observations; n event, number of events. Based on 200 simulations

In Setting 5, we vary the standard deviation of the measurement error in the longitudinal biomarker (Fig. 6). This isolates the effect of measurement error while keeping the number and timing of longitudinal observations and events constant across all scenarios (mean of 60.6 events).

JM (frequentist) strongly underestimates the association parameter and exhibits convergence difficulties as measurement error increases. JMbayes2 shows nearly unbiased estimates when measurement error is small, with 100.0% convergence. However, as measurement error increases, both bias and convergence issues increase, with convergence dropping to only 3.5% at a standard deviation of 0.5. This finding is unexpected, given that joint models are theoretically designed to account for measurement error. In contrast, joineRML (frequentist) performs the best across all scenarios, exhibiting no bias and consistent 100.0% convergence. As expected, time-varying Cox PH regression shows substantial increase in bias with increasing measurement error, reflecting its reliance on observed biomarker values. The two-stage approach shows only a modest increase in bias and maintains close to 100.0% convergence across all settings. This robustness stems from the smoothing effect of the linear mixed-effects model predictions in the first stage, which mitigates the impact of measurement error.

### Coverage probabilities

The results for the coverage probabilities for the association parameter $\:\alpha\:$ across the different simulation settings are provided in the Supplementary Material in Table 2. Overall, JMbayes2 (Bayesian) and joineRML (frequentist) consistently achieve coverage rates close to the nominal level of 95% across most settings. In contrast, JM (frequentist) shows substantially lower coverage. The two-stage approach generally performs well, with coverage probabilities fluctuating around the nominal level but occasionally falling below it in more extreme settings. The time-varying Cox PH regression yields coverage between 88.5% and 95.0% across settings 1–4, only in setting 5 the coverage goes down to 6.5% in scenarios with a larger measurement error.

### Bias in other parameters

Similar results are observed for the other parameters (longitudinal submodel: time and age effect estimates; survival submodel: age and sex effect estimates) across Settings 1a-5a (Supplementary Figs. 1–20). The JM package (frequentist) results remain highly unstable and exhibit substantial bias in most cases, even when adjusting the function’s default settings, such as increasing the number of iterations, changing the optimizer or simplifying the random effects structure. joineRML (frequentist) shows performance consistent with that observed for the association parameter: minimal to no bias in most scenarios, performing comparable to the two-stage approach. However, both frequentist packages consistently exhibit bias in the estimates for the age effect in the survival submodel across all scenarios, even when joineRML estimates all other parameters without bias. JMbayes2, the two-stage approach, and the time-varying Cox PH regression perform very similarly to the performance seen for the association parameter. Notably, all package implementations consistently estimate the sex effect in the survival model with minimal bias, even when other model parameter estimates are substantially biased. This observation is particularly relevant, as in other settings this parameter could represent a treatment effect and therefore be of key importance to a study.

### Effective sample size for JMbayes2

The ESS of the MCMC estimations in JMbayes2 of the association parameter $\:\alpha\:$ are presented in Supplementary Table 3. While most settings achieve sufficiently high ESS on average (mean ESS > 1,000), Setting 4a shows lower ESS. Despite using a high number of MCMC iterations (40,000 iterations plus warm-up of 5,000 and thinning of 20 in three parallel chains), scenarios with longitudinal time effects between − 0.03 and − 0.005 had very low ESS (mean between 75 and 423), indicating poor convergence and mixing of the MCMC chains. Only the scenario without a longitudinal time effect (${\beta}_{1}=0$) achieves a mean ESS of 4,229.        

To address this, we conduct a supplementary analysis with 100,000 MCMC iterations and more informative prior distributions for the association parameter (prior mean = -0.5, precision $\:\tau\:$ = 1; compared default: mean = 0 and $\:\tau\:$ = 0.25). While ESS increase across all scenarios (Supplementary Fig. 23; e.g., from mean ESS of 75 to 248 for $\:{\beta}_{1}=-0.03$), this improvement is modest given the substantially increased number of posterior samples (from 2,000 to 5,000 samples per chain). More importantly, bias in the association parameter estimates remains largely unchanged despite the more informative priors, suggesting that the bias in Setting 4a reflects identifiability challenges in this sparse data scenario, rather than issues resolvable through standard tuning.

### Sensitivity analysis JM package

Given substantial convergence problems observed with the JM package, we conduct sensitivity analyses to test whether algorithmic tuning or model simplification could improve performance. Specifically, we examine: (i) increased iterations (EM iterations: iter.EM = 200, quasi-Newton iterations: iter.qN = 500 compared to defaults of 50 and 150), (ii) alternative optimizer (nlminb instead of optim), and (iii) simplified random effects structure (random intercept only instead of random intercept and slope). These analyses are performed for Settings 1a, 2a, and 3a (Supplementary Figs. 24–26). None of these modifications substantially improve model fit or convergence, suggesting that the convergence issues reflect substantial implementation challenges under sparse data conditions rather than suboptimal default settings.

### Unconditional results

Our primary performance analyses condition on model convergence, as non-converged models may yield unreliable parameter estimates. To provide a complete picture, we conduct supplementary analyses including all simulation runs that result in valid point estimates. For models that complete but fail diagnostic criteria, we extract parameter estimates; models that crash or produce non-finite values are coded as missing.

**Fig. 7:**
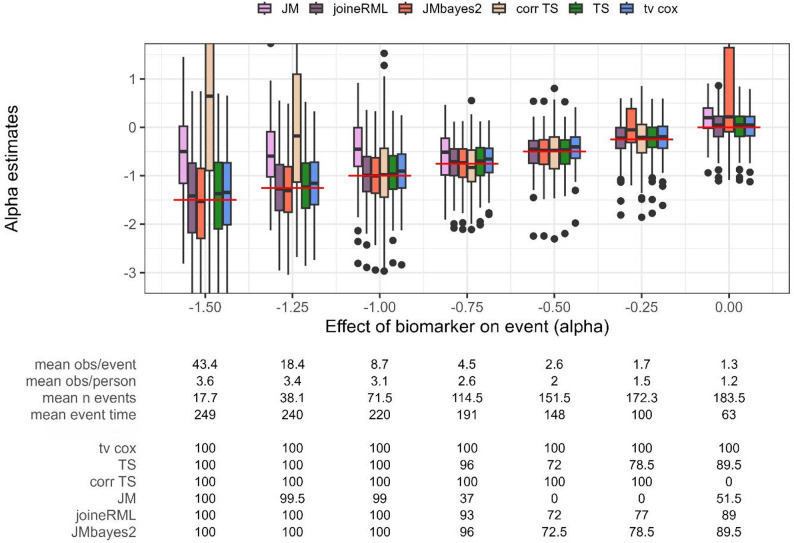
Converged and unconverged estimates for the association parameter α (y axis) while varying the association parameter itself (x axis). Red horizontal line indicates true parameter value. tv cox, time-varying Cox proportional-hazards regression; TS, two-stage approach; JM and joineRML, frequentist joint models; JMbayes2, Bayesian joint model. Based on 200 simulations

Figure 7 shows unconditional results for Setting 2a (varying association parameter $\:\alpha\:$), results for the other simulation scenarios are in Supplementary Figs. 27–30. For most implementations (JM, joineRML, time-varying Cox PH regression, two-stage approach, and corrected two-stage approach), unconditional results closely resemble the conditional results presented earlier, as these either converge reliably or fail completely without producing extractable estimates. JMbayes2, however, exhibits notable differences. In low-event scenarios ($\:\alpha\:\le\:\:-1.0$), including non-converged runs reduces apparent bias, suggesting some non-converged runs stop closer to the true value than converged runs potentially trapped in local optima. Conversely, in low observation-to-event scenarios ($\:\alpha\:\ge\:-0.5$), including non-converged models increases both bias and variability substantially, with numerous extreme outliers reflecting unreliable estimates.

### Results for only converged settings

Supplementary Figs. 31–35 present results restricted to simulation runs in which all five main implementations (JM, joineRML, JMbayes2, two-stage approach, and time-varying Cox PH regression) successfully converged. This subset analysis reveals several important patterns. JM (frequentist) continues to perform worst even within this restricted set, exhibiting substantial bias across settings. By contrast, JMbayes2, joineRML (frequentist), the two-stage approach, and time-varying Cox PH regression perform at comparable levels: in settings where JMbayes2 shows bias, joineRML and the simpler methods exhibit similar bias. This pattern suggests that the superior performance of joineRML, the two-stage approach and time-varying Cox PH regression observed in the main analysis, conditioning on method-specific convergence, stems primarily from their ability to produce usable estimates in settings where JMbayes2 fails to converge. In other words, the practical advantage of joineRML and the simpler implementations in challenging data scenarios reflects their reliability (consistent convergence) rather than fundamentally superior bias properties in datasets where all packages can be successfully applied.

### Model fit to original BIS data

To contextualize our simulation results, we apply the five R packages to the original BIS data, comprising 2069 individuals with an average of 3.4 longitudinal observations and 1027 events, resulting in an observation-to-event ratio of 6.9. The model specifications follow those used in our simulation study: For the longitudinal submodel, log(eGFR) is modelled as a function of age at baseline and time since baseline (in weeks), with random intercepts and slopes. The survival submodel includes age and sex as covariates in addition to the current value of log(eGFR).

Table 3 presents the parameter estimates and 95% confidence (CI) or credibility (CrI) intervals, respectively, for all five approaches. The JM package estimates a stronger association between log(eGFR) and survival (α = -1.07, 95% CI: -1.28 to -0.86) compared to the uncorrected two-stage approach (α = -0.55, 95% CI: -0.77 to -0.33) and the time-varying Cox PH regression (α = -0.70, 95% CI: -0.93 to -0.46). JMbayes2 and joineRML produce estimates between these extremes (JMbayes2: α = -0.91, 95% CrI: -1.13 to -0.68; joineRML: α = -0.89, 95% CI: -1.08 to -0.71). All models estimate similar trajectories for the longitudinal process, with comparable estimates for the fixed effects of age and time. The estimate for the time effect is consistently around − 0.039, indicating a decrease in log(eGFR) over time (Table 3).

**Table 3:** Parameter estimates along with 95% confidence/credibility intervals for the Berlin Initiative Study (BIS) data. Time effect estimates were converted into years

Parameter	time-varying Cox PH regression	uncorrected two-stage approach	JM	joineRML	JMbayes2
*Longitudinal*					
Intercept		5.85 (5.72, 5.98)	5.86 (5.73, 5.99)	5.85 (5.72, 5.99)	5.85 (5.72, 5.98)
age		-0.023 (-0.024, -0.021)	-0.023 (-0.024, -0.021)	-0.023 (-0.024, -0.021)	-0.023 (-0.024, -0.021)
time [years]		-0.038 (-0.039, -0.037)	-0.039 (-0.04, -0.037)	-0.037 (-0.04, -0.037)	-0.039 (-0.04, -0.037)
*Survival*					
log_gfr	-0.70 (-0.93, -0.46)	-0.55 (-0.77, -0.33)	-1.07 (-1.28, -0.86)	-0.89 (-1.08, -0.71)	-0.91 (-1.13, -0.68)
age	0.09 (0.08, 0.11)	0.07 (0.06, 0.08)	0.09 (0.08, 0.10)	0.11 (0.11, 0.12)	0.09 (0.07, 0.11)
sex female	-0.31 (-0.44, -0.18)	-0.18 (-0.30, -0.07)	-0.39 (-0.51, -0.27)	-0.33 (-0.45, -0.21)	-0.33 (-0.46, -0.20)

In line with our simulation results, the differences in estimates between JMbayes2, joineRML, and the two-stage approach in the BIS data are consistent with the patterns observed across simulation scenarios: JMbayes2 tends to estimate stronger associations than the two-stage approach (see Supplementary Fig. 37) and time-varying Cox PH regression while joineRML’s estimates typically fall in between. However, without knowledge of the true underlying association in the BIS data, it remains unclear which method provides the most accurate estimates in this specific case.

## Discussion

This study fills a gap in the joint model literature by providing an empirical evaluation of how commonly used R package implementations of joint models and alternative approaches behave under sparse-data conditions, offering crucial insights for researchers facing data limitations in real-world applications. The objective of this study was to compare implementations for modelling the association between longitudinally measured biomarker and the hazard of an event. In particular, we focused on joint models, with the aim of gaining a deeper understanding of specific scenarios in which these implementations perform superior or inferior compared to the two-stage approach and time-varying Cox PH regression. To this end, we conducted an extensive simulation study based on real data on kidney function from a cohort study. Varying different parameters in the data generating process (observation density, association parameter $\:{\upalpha\:}$, baseline hazard, slope over time, and measurement error), we examined how the number of events and the number of longitudinal biomarker measurements impact bias in the parameter estimates.

### Summary of results and comparison to previous studies

Our simulation study compared three commonly used R package implementations of joint models (JM, joineRML, JMbayes2) with the two-stage approach (nlme, survival) and time-varying Cox PH regression (survival). The findings reveal substantial variability even among different joint model implementations within the same statistical framework, highlighting that software choice can be as influential as the choice of framework. The JM package consistently exhibits the largest bias and frequent convergence issues across settings, with convergence frequencies often < 50.0%. Sensitivity analyses – testing increased iterations, alternative optimizers, and simplified random effects structures, suggest fundamental algorithmic limitations for the data structures considered. In contrast, the joineRML package produced approximately unbiased parameter estimates for most parameters with reliable convergence frequencies across all scenarios, comparable to the two-stage approach, making it a robust frequentist joint model implementation suitable for diverse data settings. However, both frequentist implementations (JM and joineRML) consistently and substantially underestimated the age effect in the survival submodel, even when other parameters were estimated without bias. This systematic bias pattern suggests potential algorithmic or implementation-specific issues in handling baseline covariates in the survival model, warranting caution when interpreting these parameters. The Bayesian joint model implementation (JMbayes2) generally showed unbiased parameter estimates in data-rich settings but increased bias and convergence issues in settings with a small number of events (< 70) or with observation-to-event ratio smaller two. Notably, JMbayes2 did not exhibit the systematic underestimation of the age effect observed in the frequentist packages. The implementations of the two simpler methods, two-stage approach and time-varying Cox PH regression, demonstrated consistent performance across settings. The two-stage approach (nlme and survival) generally slightly outperformed time-varying Cox PH regression (survival) in bias, while time-varying Cox PH regression achieved 100.0% convergence across all settings.

Unlike previous studies, our simulation results suggest that the choice of R package implementation can matter as much as the choice of modelling framework, with some joint model implementations failing to outperform simpler approaches in data-scarce scenarios. Both JM and JMbayes2 deteriorate in more extreme settings with a small number of events or sparse longitudinal data, particularly when events occur early in the observation period, limiting the amount of longitudinal data. This is inherent to joint models’ reliance on reconstructing individual trajectories from limited measurements and estimating survival parameters simultaneously, whereas time-varying Cox PH regression directly uses observed values and the two-stage approach might benefit in those challenging scenarios from pre-smoothing the trajectories through mixed-effects model predictions in the first stage. In contrast, joineRML and the implementations of the simpler methods demonstrated greater robustness under these conditions, especially when the ratio of longitudinal observations to events dropped below two. However, notably all R packages consistently estimated the group effect in the survival model with minimal bias, even when other model parameter estimates were highly biased, consistent with prior studies by Alvares and Leiva-Yamaguchi [16] and Barrett et al. [23]. Moreover, our simulations indicate that the two-stage approach and the time-varying Cox PH regression tend to estimate smaller effects than JMbayes2, in line with the findings by Prentice [10] and Goncalves et al. [25].

Prior simulation studies, including those by Barrett et al. [23], Mauff et al. [14], Leiva-Yamaguchi and Alvares [15], Alvares and Leiva-Yamaguchi [16], Huong et al. [18], and Desmée et al. [12], included up to 10, 15, 15, 20, 25, and 36 longitudinal observations, respectively. Given the high number of longitudinal observations in these studies, our simulation results suggest that it is unsurprising that joint model implementations performed well and with minimal bias. Our findings highlight that JMbayes2, in particular, excels in such data-rich settings but may struggle in more constrained settings with very few longitudinal observations compared to the number of events, settings not uncommon in medical studies.

### Recommendations for applied researchers

Based on our findings, we offer the following practical guidance for choosing among R package implementations when analyzing longitudinal biomarker data with time-to-event outcomes:

For data-rich scenarios (observation-to-event ratios > 2–3 and adequate numbers of events > 70): JMbayes2 generally provides good performance and is recommended when unbiased estimates are required while accounting for measurement error and informative dropout. joineRML is a robust frequentist alternative across most data settings, offering reliable convergence and approximately unbiased estimates for association parameters. However, researchers should interpret the effects of baseline covariates (e.g. age effects) from the survival model cautiously, as consistent underestimation was observed with both frequentist joint model packages.

For data-sparse scenarios (observation-to-event ratios < 2 or < 70 events): Convergence diagnostics should be carefully evaluated across multiple implementations. Both joineRML and the two-stage approach consistently provided relatively unbiased results while avoiding convergence issues. However, the two-stage approach does not account for the uncertainty from the first-stage predictions, leading to underestimated standard errors in the survival model. If a joint model framework is preferred for theoretical reasons (e.g., to explicitly model informative dropout), joineRML is more robust than JMbayes2 under sparse data, with the caveat regarding baseline covariate effect estimation. For JMbayes2, expert prior specification and extended MCMC runs are advisable, with particular attention to posterior diagnostics indicating identifiability issues.

In all settings, we recommend comparing results across multiple software implementations as a sensitivity analysis. Substantial discrepancies may indicate identifiability issues or model misspecification. Additionally, researchers should report convergence diagnostics and clearly state the software and version used, as implementation choices can substantially affect results. Notably, when the primary goal is estimating treatment effects in the survival model (rather than the biomarker-event association), our finding suggest that these estimates remain robust across packages even when other parameters are biased.

Finally, researchers may also consider running simulations based on and modifying our code to evaluate specific R package implementations in settings mimicking their data.

### Strengths and limitations

A novel aspect of our simulation study is that by varying several parameters and as a consequence the numbers of events and longitudinal observations, we demonstrated that joint model implementations can be effective even with fewer longitudinal observations, but require a sufficient ratio of longitudinal observations to events to show advantages compared to simpler approaches. To our knowledge, this has not been extensively explored in the joint model literature. Another strength is the inclusion of three different R packages for joint modeling (JM, joineRML, and JMbayes2), which revealed substantial variability in performance even among implementations based on similar statistical principles. This highlights that general conclusions about “joint models” versus “two-stage approaches” can be misleading when they actually reflect differences between estimation frameworks, specific software implementations, and their default settings. Our findings thus provide implementation-specific guidance that is directly relevant for applied researchers. Rappl et al. [35] have shown that different implementations of the same method can lead to differences in results, for example, due to variations in techniques for integral approximation. Therefore, our findings regarding the relative performance of frequentist versus Bayesian joint models should be interpreted as a comparison of specific R package implementations rather than a comparison of frequentist versus Bayesian statistical philosophies more broadly. Observed performance differences may reflect implementation choices (e.g., numerical integration methods, optimization algorithms, default prior specifications in JMbayes2) rather than inherent differences between statistical frameworks.

An inherent limitation of our comparison is that the included R packages differ along several dimensions simultaneously: the parameterization of the longitudinal–survival association (e.g., current value in JM and JMbayes2 versus shared random effects in joineRML), the estimation framework (Bayesian versus likelihood-based), and the default implementation choices within each package (e.g., priors, MCMC settings, optimization algorithms, quadrature methods). Because these dimensions vary at the same time, observed differences in performance cannot be attributed to any single factor. Rather than attempting to isolate individual sources of variation, our study is explicitly designed as a practical evaluation of how commonly used R packages perform in sparse-data scenarios, mostly under their default settings. Therefore, differences in parameterization and estimation strategy are part of the comparison, and the contribution of this work lies in providing applied researchers with guidance on what to expect from each package as they would typically use it in practice.

Simulations were based on data from an existing cohort study, allowing for realistic assumptions regarding effect sizes. However, this limits generalizability of our findings. To address the limitation of simulating only linear trajectories, we conducted an additional simulation setting with nonlinear biomarker trajectories modelled using cubic B-splines with three basis functions. This extension revealed that nonlinear trajectories require more longitudinal observations for unbiased estimates compared to linear settings. All R packages performed similarly to the linear settings when sufficient measurements were available.

Our simulation used Weibull data generation with Weibull model specification for the JM package, penalized B-splines (default) for JMbayes2, and semi-parametric Cox baseline hazards (leaving the baseline hazard unspecified) for joineRML, time-varying Cox PH regression, and the survival model in the two-stage approach. The good performance of JMbayes2 despite this mild misspecification suggests robustness of the flexible spline approach, which may be advantageous in practice where true baseline hazard forms are unknown.

Alternative scenarios not explored in our design may reveal different performance patterns, including different association structures (random intercept only, random slope only, cumulative effects, time-lagged associations), non-proportional or time-varying baseline hazards, non-Weibull hazard shapes, more than one and potentially non-linear biomarker trajectories, more complex random effects structures, and irregular or informative visit schedules. Each of these dimensions represents an important direction for future research. We emphasize that our specific findings such as the observation-to-event ratio threshold of approximately 2 should be interpreted as applying to the particular combinations of features we examined rather than as universal rules.

## Conclusion

This simulation study provides practical guidance for R package selection when analyzing longitudinal biomarker data with survival outcomes. Comparing three joint model implementations (JM, joineRML, JMbayes2) mainly under their default settings with the two-stage approach and time-varying Cox PH regression, we demonstrate that the Bayesian package (JMbayes2) generally performs well in data-rich scenarios but shows increased bias and convergence problems when event numbers are low (< 70) or observation-to-event ratios below 2. Under these challenging but common conditions, the frequentist joint model package joineRML, the two-stage approach and time-varying Cox PH regression demonstrate greater robustness.

## Supplementary Information

Supplementary Material 1.

## Data Availability

The Berlin Initiative Study (BIS) data are available from the study PI upon reasonable request and subject to BIS data access policies. The R code used for the simulations is available at https://github.com/jilhee/jm_comparison.
