# Supplementary material for: Modelling longitudinal and time-to-event data: a phase IV simulation study comparing R package implementations of joint models with time-varying Cox proportional-hazards regression, and the two-stage approach
Source: BMC Med Res Methodol. 2026 May 14;26:116. doi: 10.1186/s12874-026-02875-4 (PMC13177884; doi:10.1186/s12874-026-02875-4)
Supplement: Supplementary file 1 — Supplementary Material 1. [file 12874_2026_2875_MOESM1_ESM.docx]

# Supplement

## Simulation results

**Setting 1a**


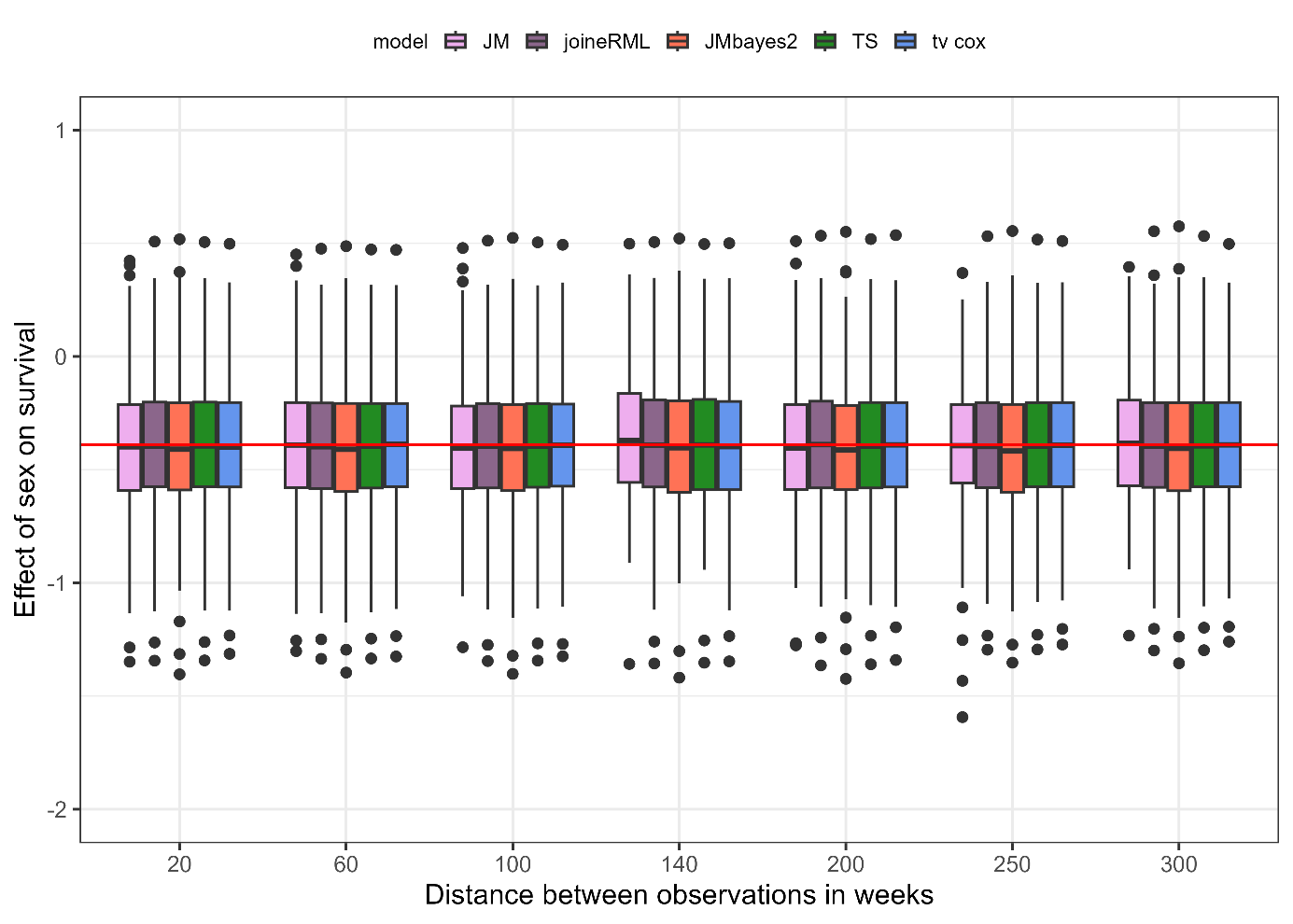


Figure 1 Estimates for the sex parameter in survival model (y axis) while varying the distance between longitudinal observations of biomarker and thus their density (x axis). tv cox, time-varying Cox proportional-hazards regression; TS, two-stage approach; JM and joineRML, frequentist joint models; JMbayes2, Bayesian joint model. Based on 200 simulations.


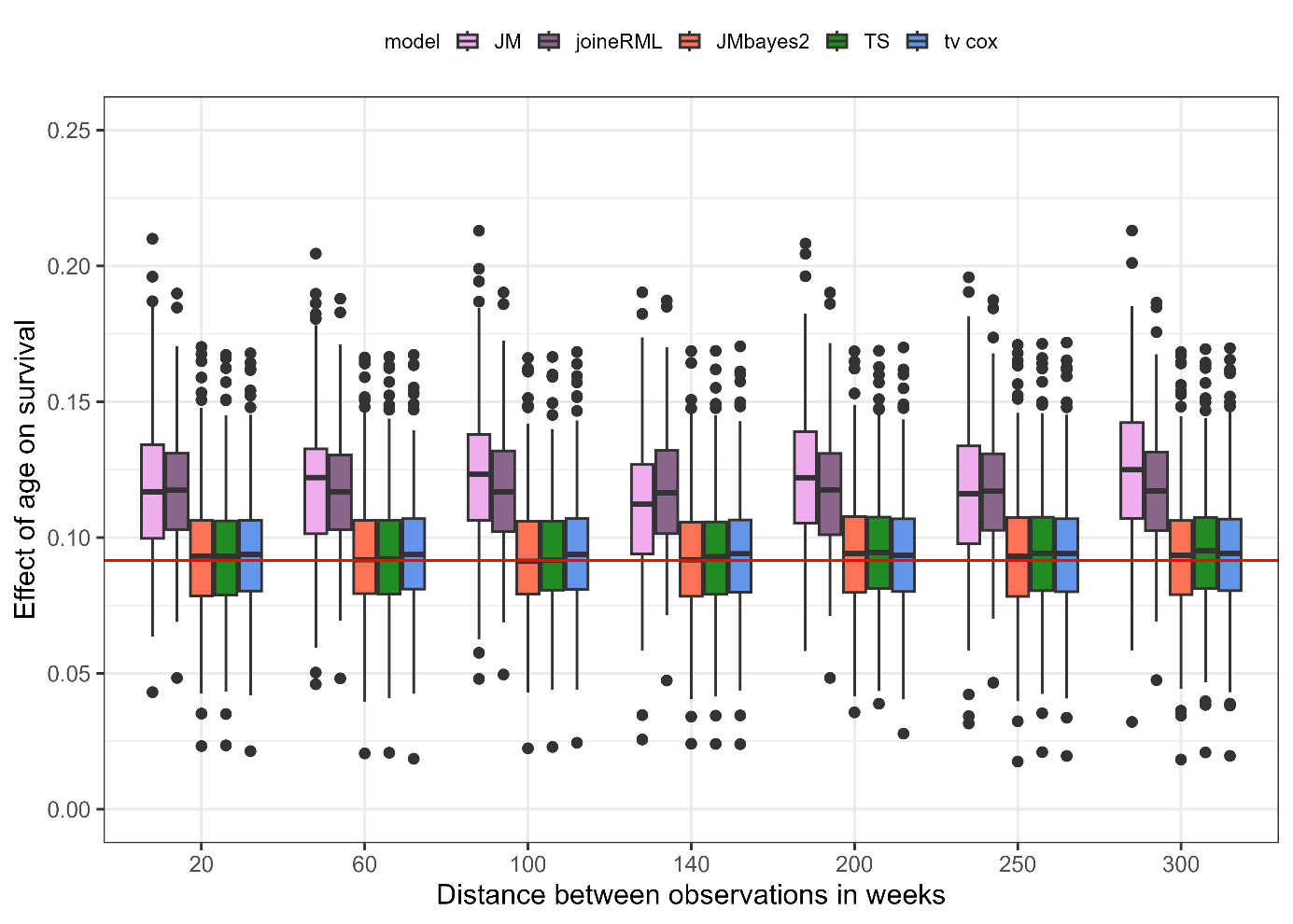


Figure 2 Estimates for the age parameter in survival model (y axis) while varying the distance between longitudinal observations of biomarker and thus their density (x axis). tv cox, time-varying Cox proportional-hazards regression; TS, two-stage approach; JM and joineRML, frequentist joint models; JMbayes2, Bayesian joint model. Based on 200 simulations.


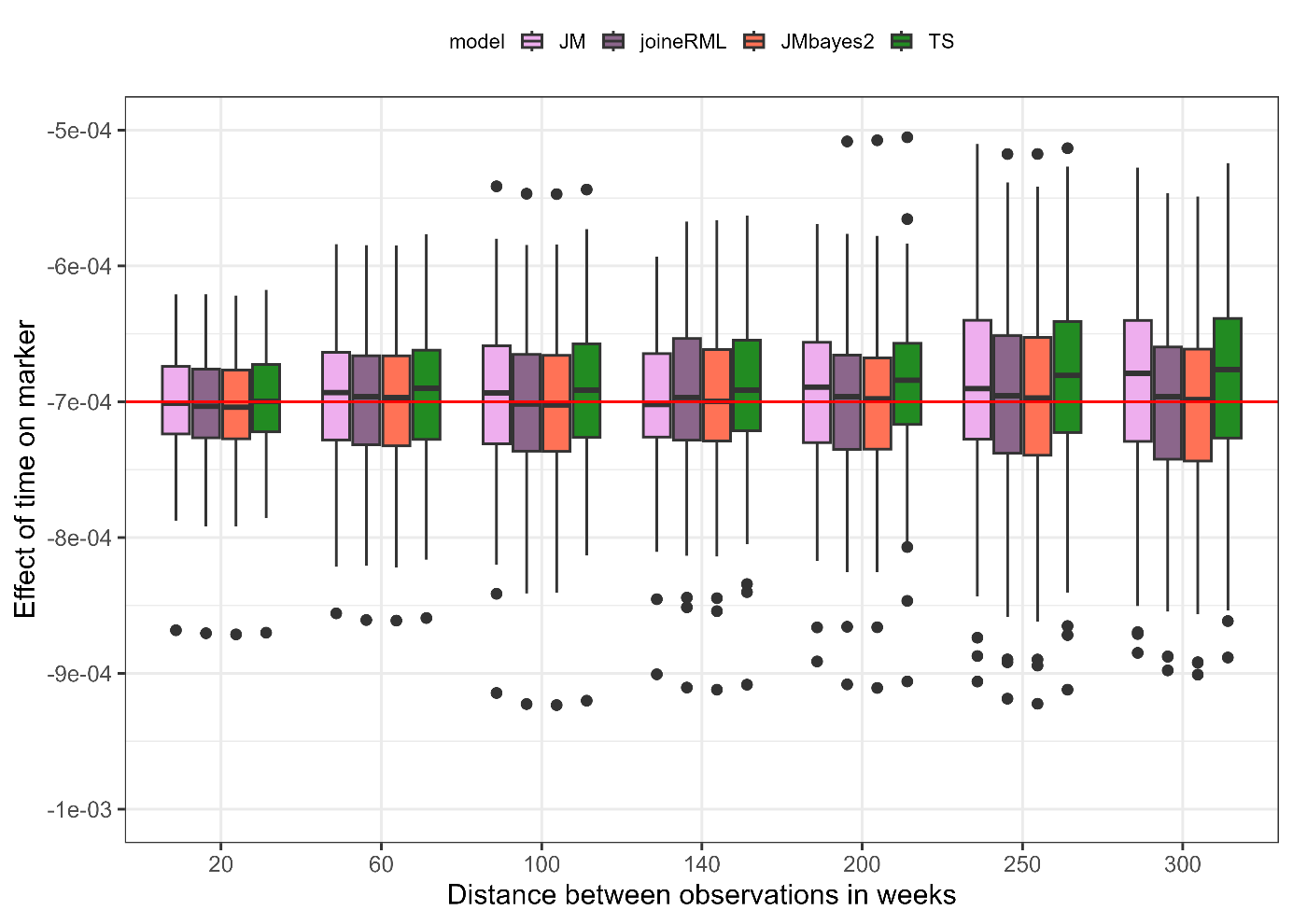


Figure 3 Estimates for the time parameter in longitudinal model (y axis) while varying the distance between longitudinal observations of biomarker and thus their density (x axis). tv cox, time-varying Cox proportional-hazards regression; TS, two-stage approach; JM and joineRML, frequentist joint models; JMbayes2, Bayesian joint model. Based on 200 simulations.


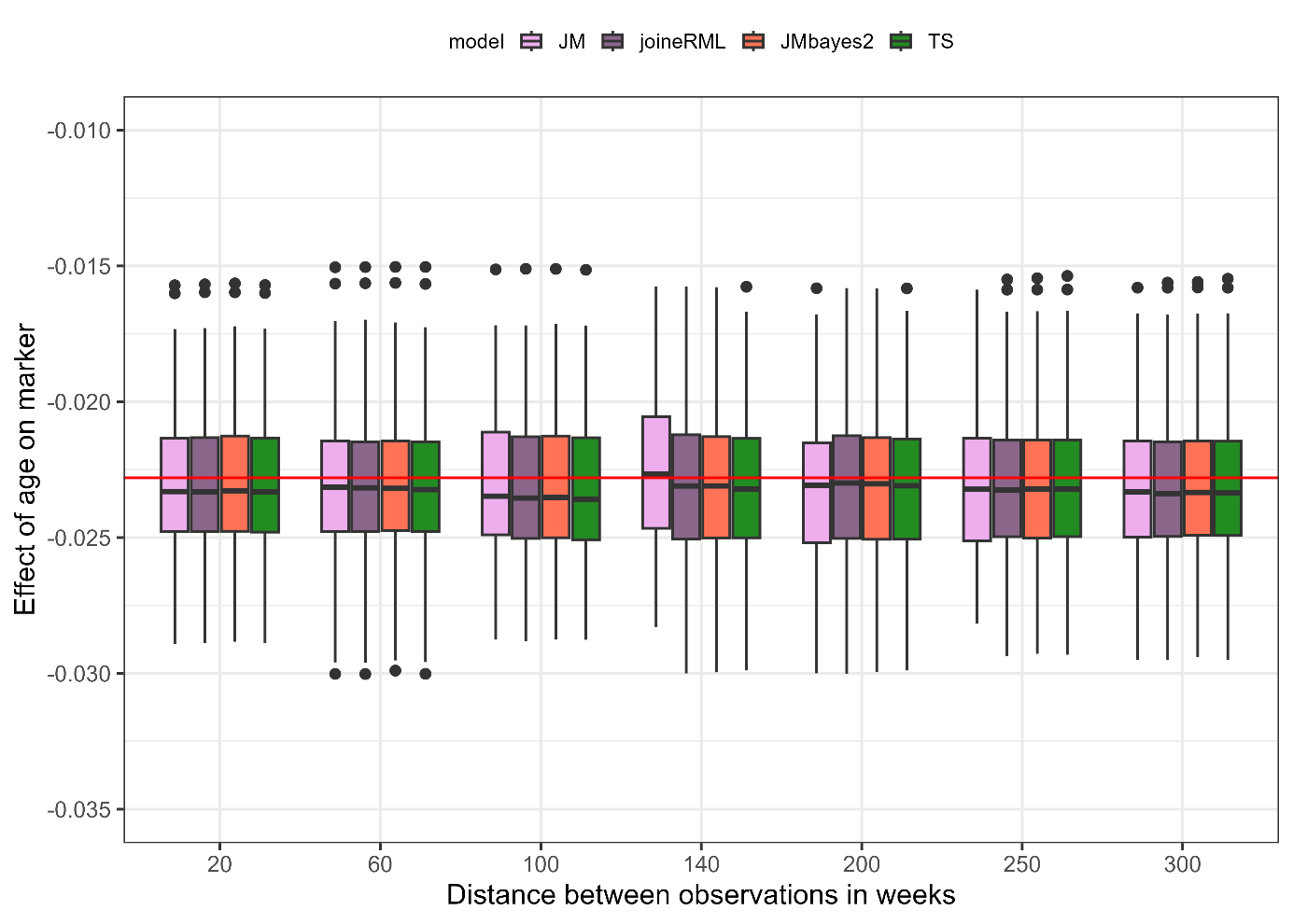


Figure 4 Estimates for the age parameter in longitudinal model (y axis) while varying the distance between longitudinal observations of biomarker and thus their density (x axis). tv cox, time-varying Cox proportional-hazards regression; TS, two-stage approach; JM and joineRML, frequentist joint models; JMbayes2, Bayesian joint modelBased on 200 simulations.

**Setting 2a**


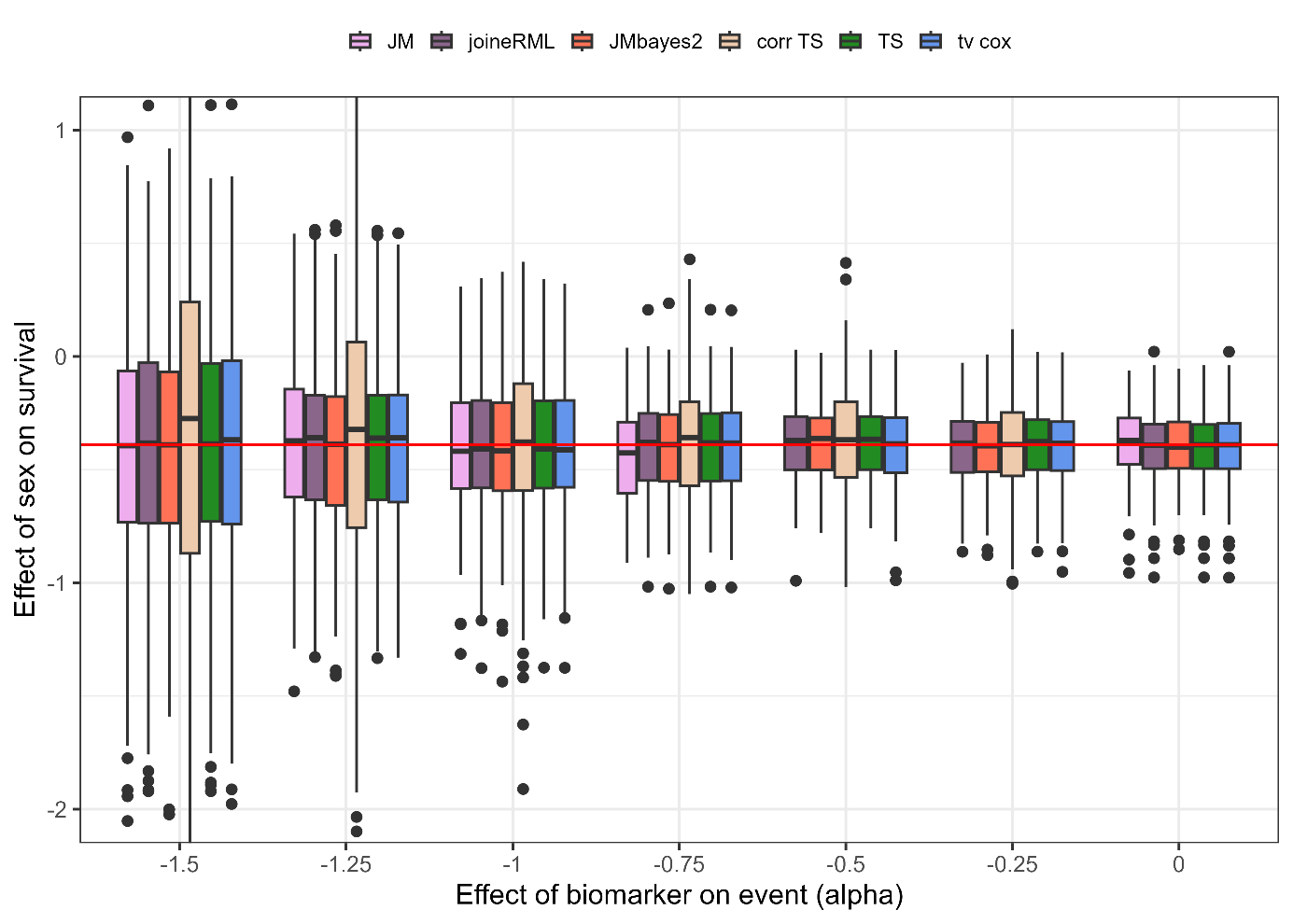


Figure 5 Estimates for the sex parameter in survival model (y axis) while varying the association parameter α (x axis). tv cox, time-varying Cox proportional-hazards regression; TS, two-stage approach; JM and joineRML, frequentist joint models; JMbayes2, Bayesian joint model. Based on 200 simulations.


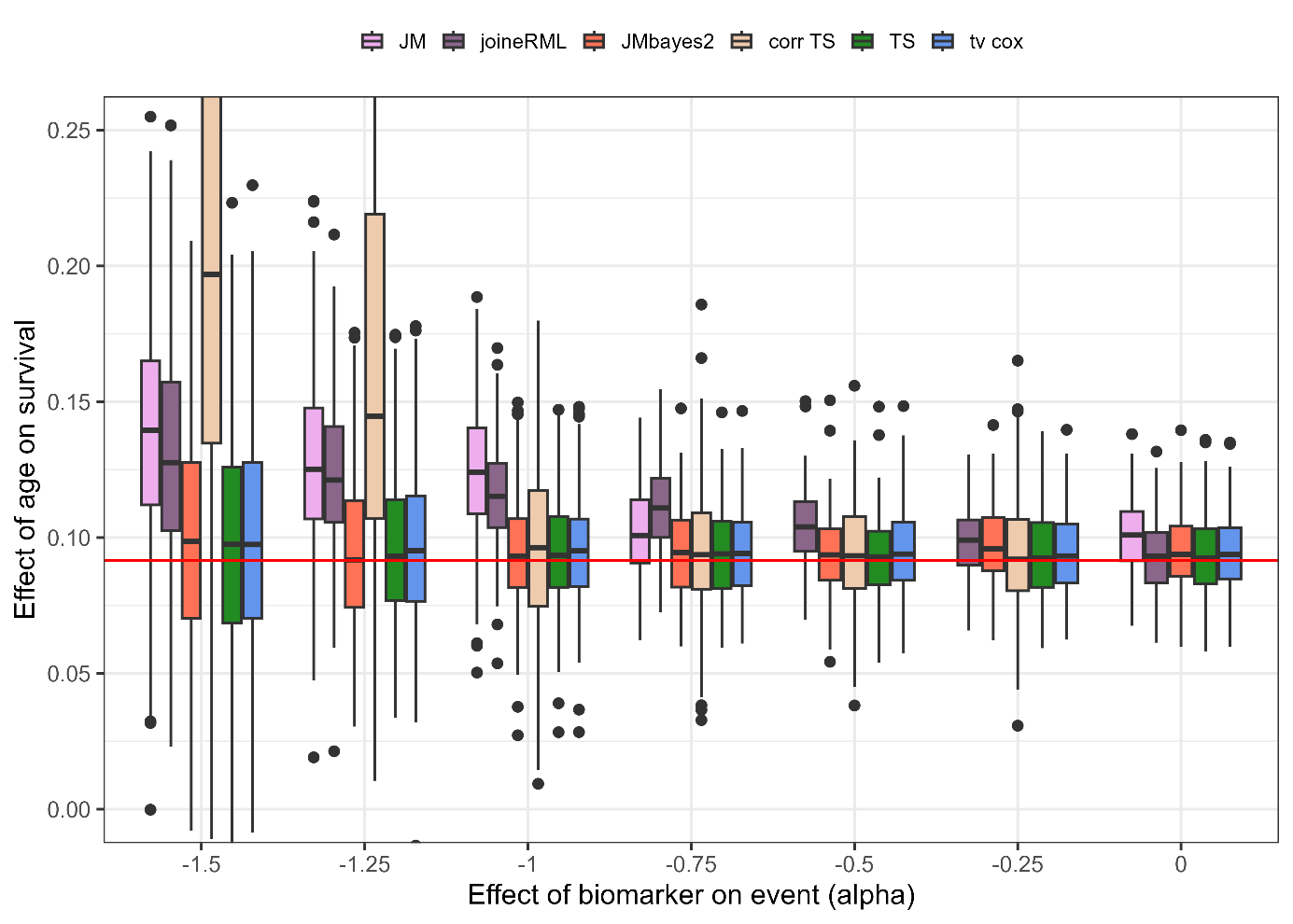


Figure 6 Estimates for the age parameter in survival model (y axis) while varying the association parameter α (x axis). tv cox, time-varying Cox proportional-hazards regression; TS, two-stage approach; JM and joineRML, frequentist joint models; JMbayes2, Bayesian joint model. Based on 200 simulations.


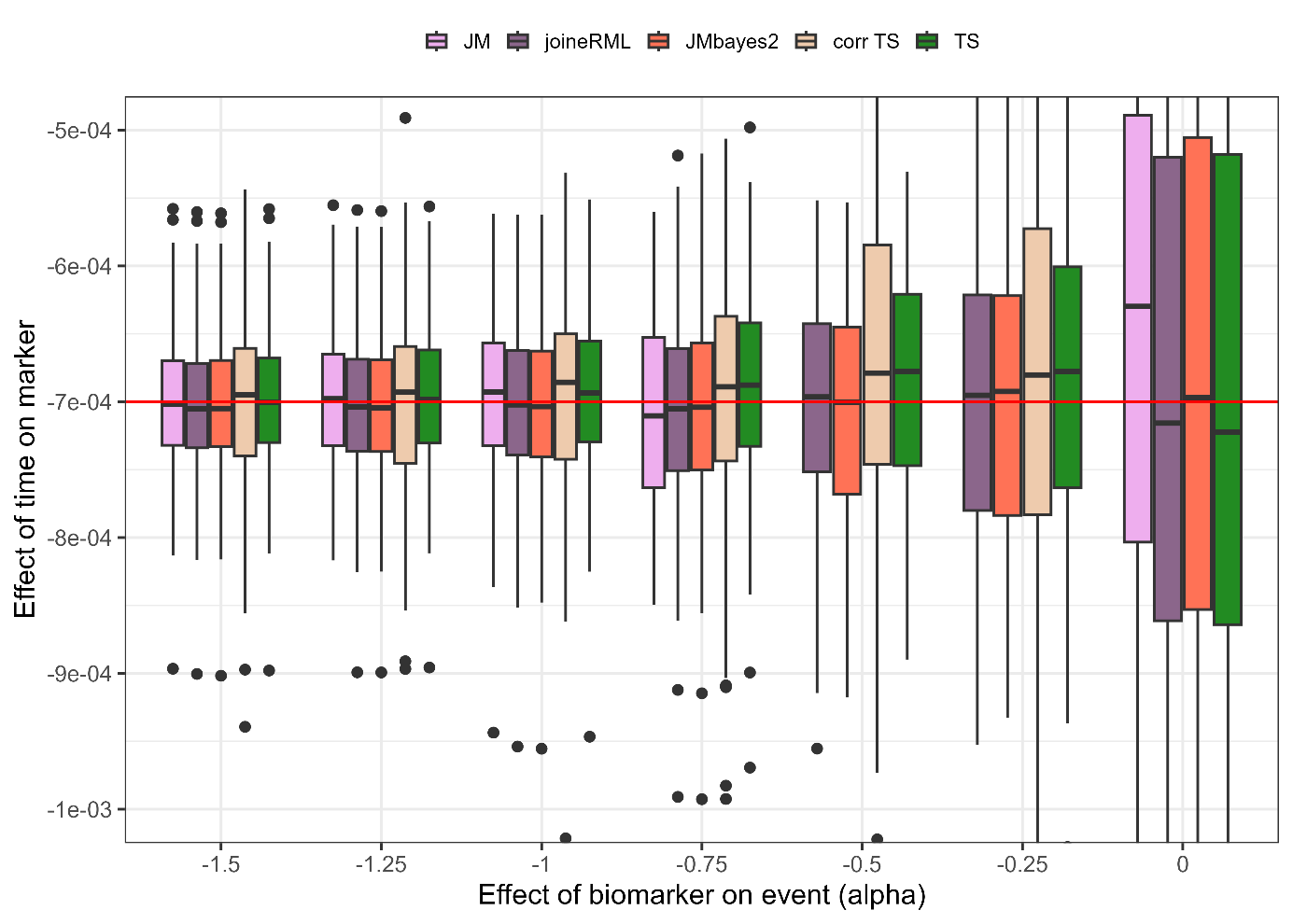


Figure 7 Estimates for the time parameter in longitudinal model (y axis) while varying the association parameter α (x axis). tv cox, time-varying Cox proportional-hazards regression; TS, two-stage approach; JM and joineRML, frequentist joint models; JMbayes2, Bayesian joint model.. Based on 200 simulations.


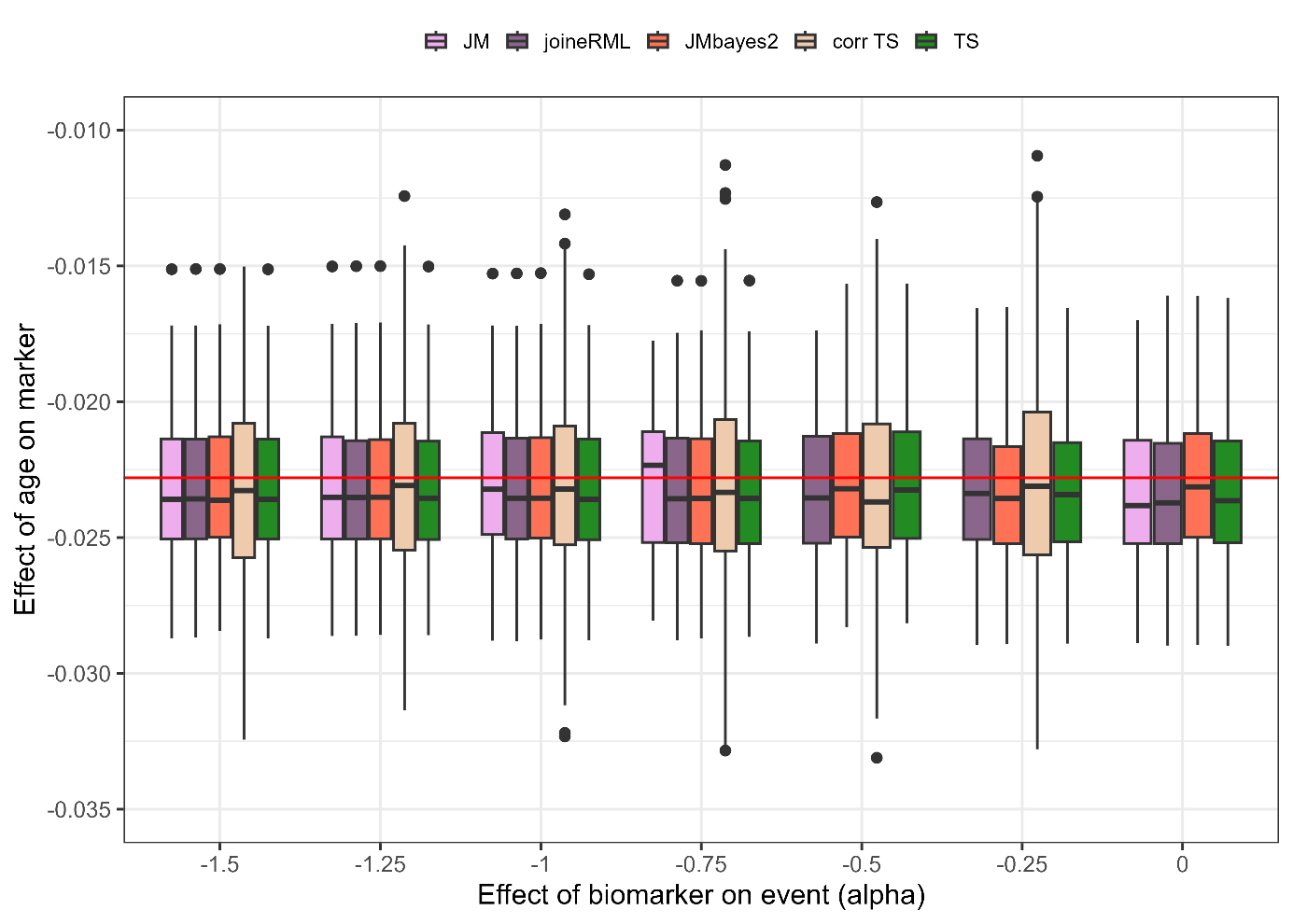


Figure 8 Estimates for the age parameter in longitudinal model (y axis) while varying the association parameter α (x axis). tv cox, time-varying Cox proportional-hazards regression; TS, two-stage approach; JM and joineRML, frequentist joint models; JMbayes2, Bayesian joint model. Based on 200 simulations.

**Setting 3a**


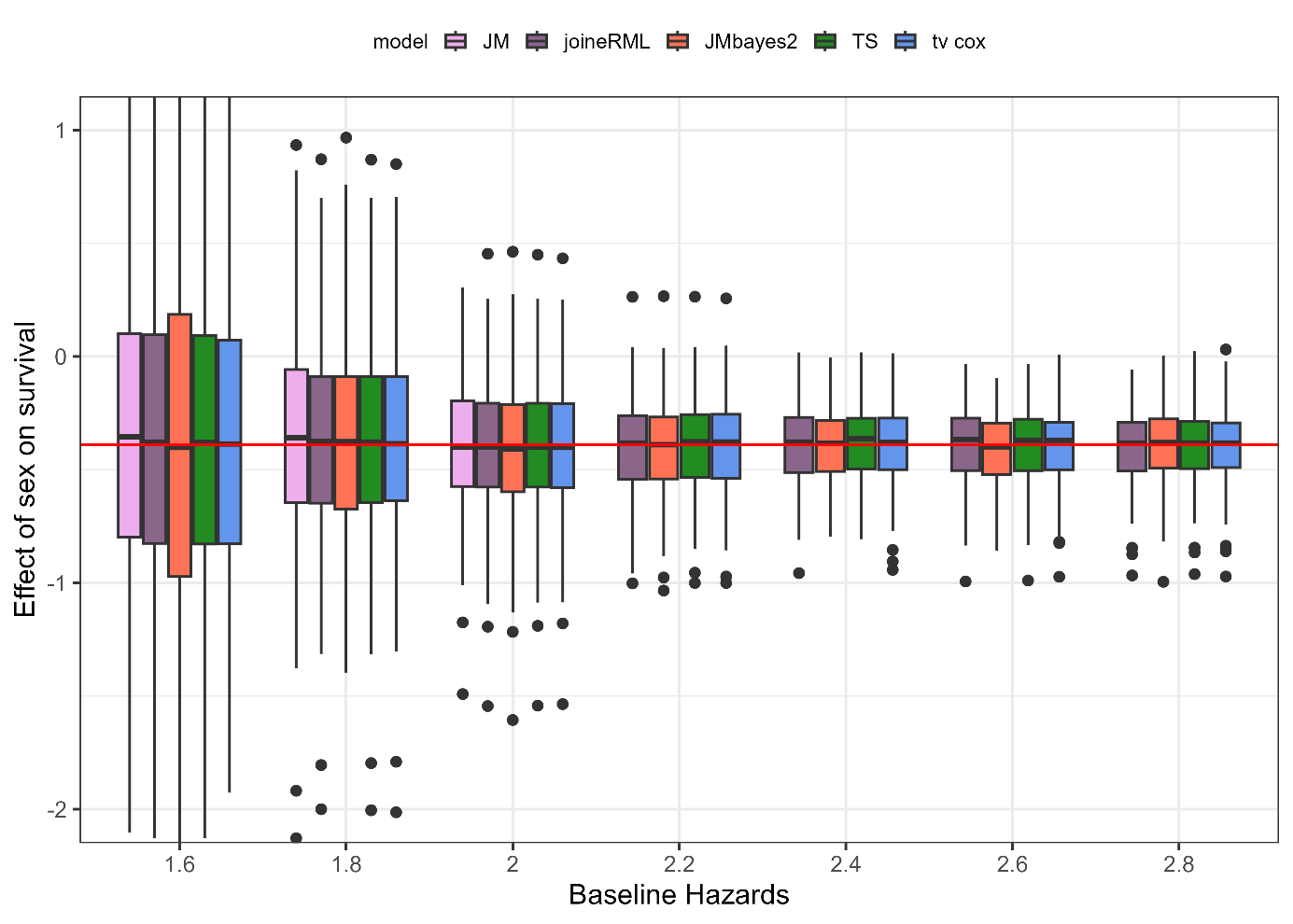


Figure 9 Estimates for the sex parameter in survival model (y axis) while varying the baseline hazard (x axis). tv cox, time-varying Cox proportional-hazards regression; TS, two-stage approach; JM and joineRML, frequentist joint models; JMbayes2, Bayesian joint model. Based on 200 simulations.


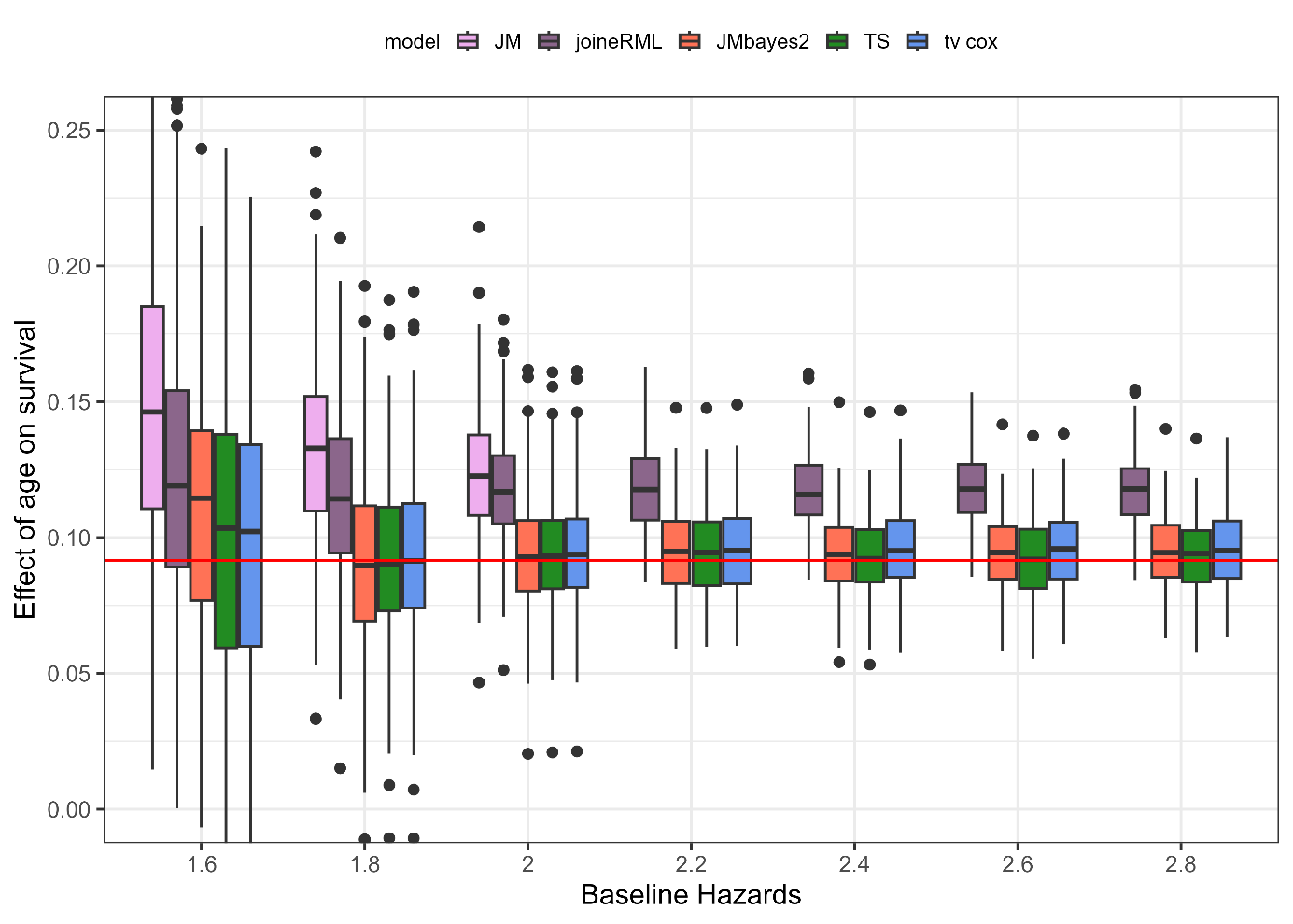


Figure 10 Estimates for the age parameter in survival model (y axis) while varying the baseline hazard (x axis). tv cox, time-varying Cox proportional-hazards regression; TS, two-stage approach; JM and joineRML, frequentist joint models; JMbayes2, Bayesian joint model. Based on 200 simulations.


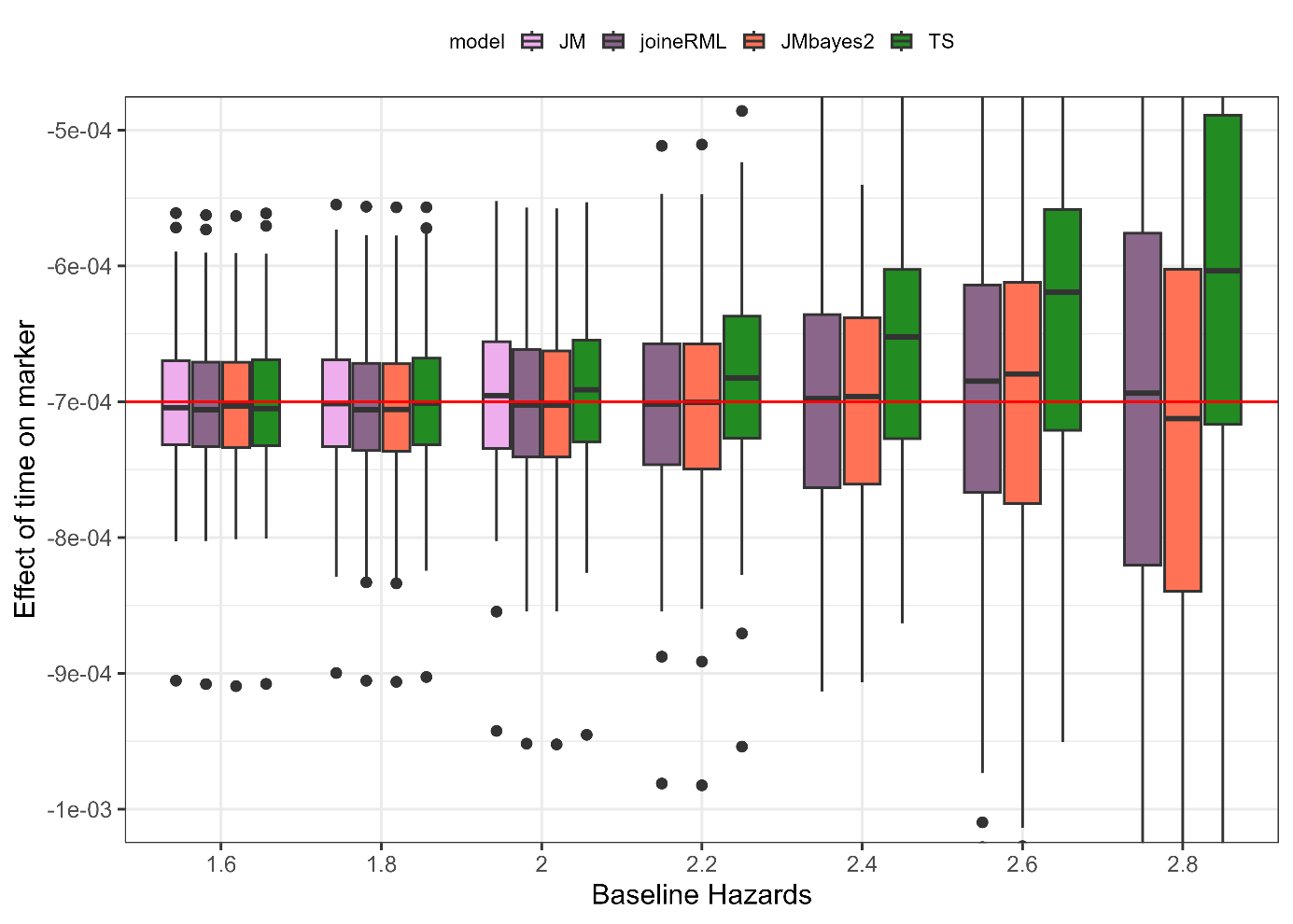


Figure 11 Estimates for the time parameter in longitudinal model (y axis) while varying the baseline hazard (x axis). tv cox, time-varying Cox proportional-hazards regression; TS, two-stage approach; JM and joineRML, frequentist joint models; JMbayes2, Bayesian joint model. Based on 200 simulations.


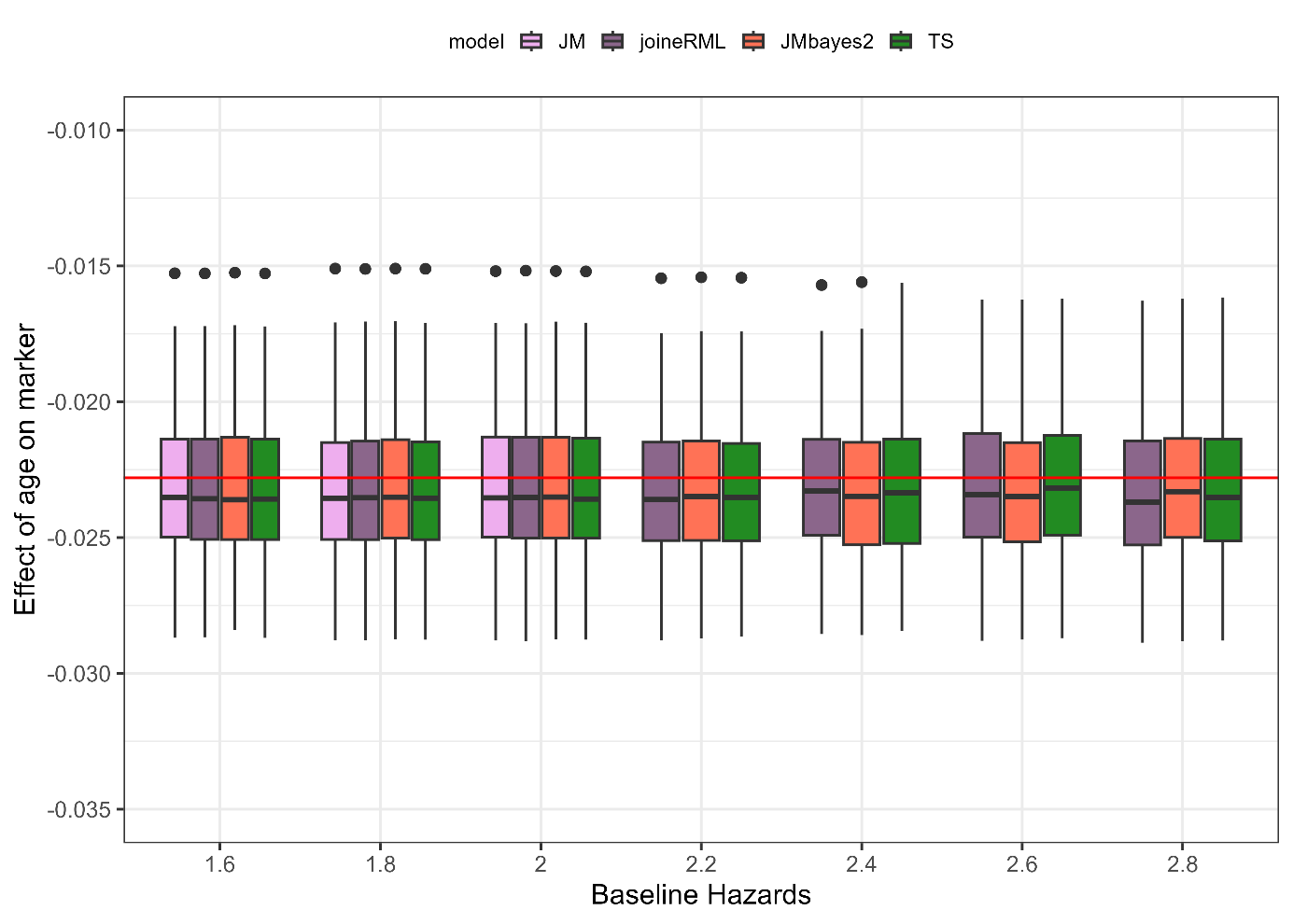


Figure 12 Estimates for the age parameter in longitudinal model (y axis) while varying the baseline hazard (x axis). tv cox, time-varying Cox proportional-hazards regression; TS, two-stage approach; JM and joineRML, frequentist joint models; JMbayes2, Bayesian joint model. Based on 200 simulations.

**Setting 4a**


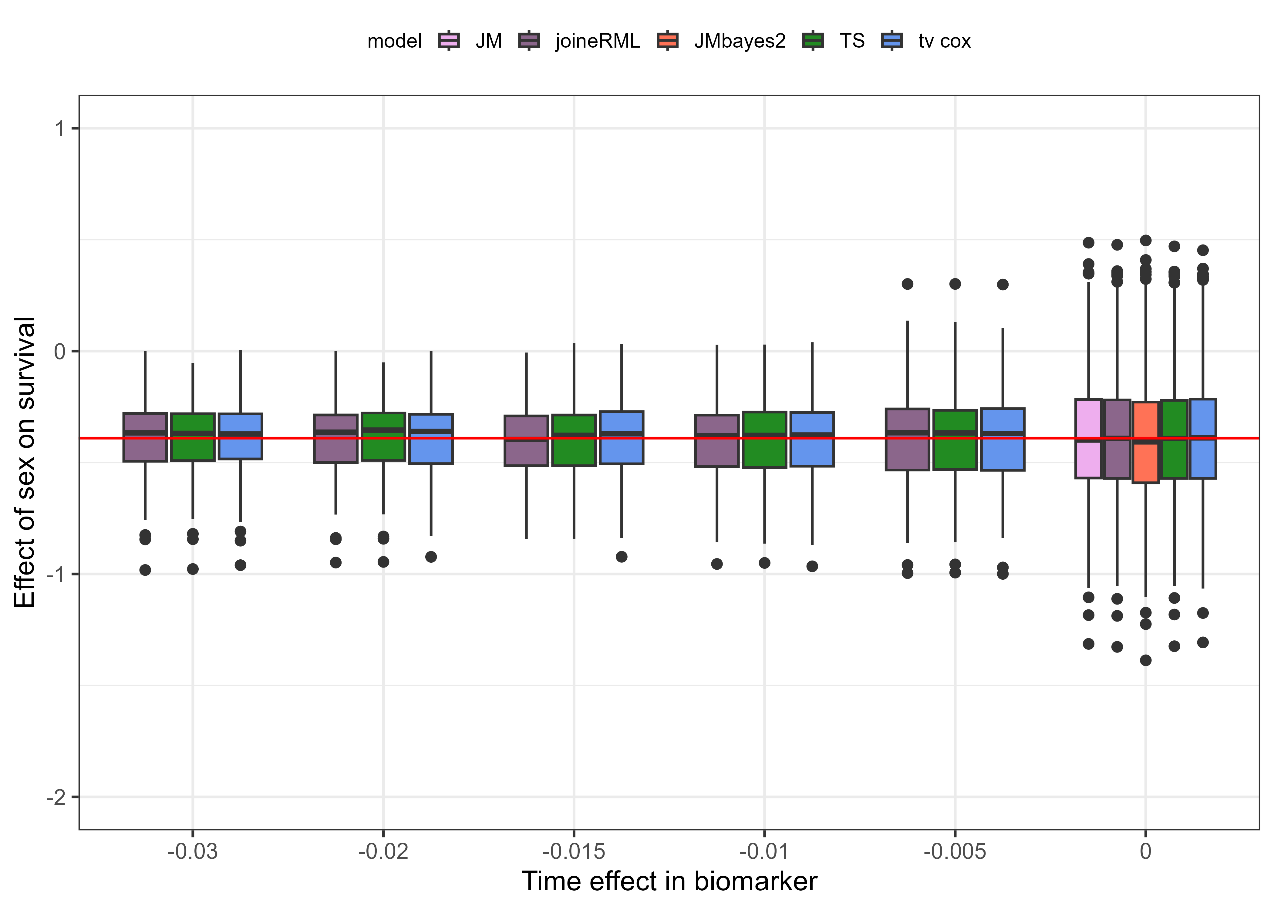


Figure 13 Estimates for the sex parameter in survival model (y axis) while varying the time effect in the longitudinal submodel (x axis). tv cox, time-varying Cox proportional-hazards regression; TS, two-stage approach; JM and joineRML, frequentist joint models; JMbayes2, Bayesian joint model. obs, number of observations; n events, number of events. Based on 200 simulations.


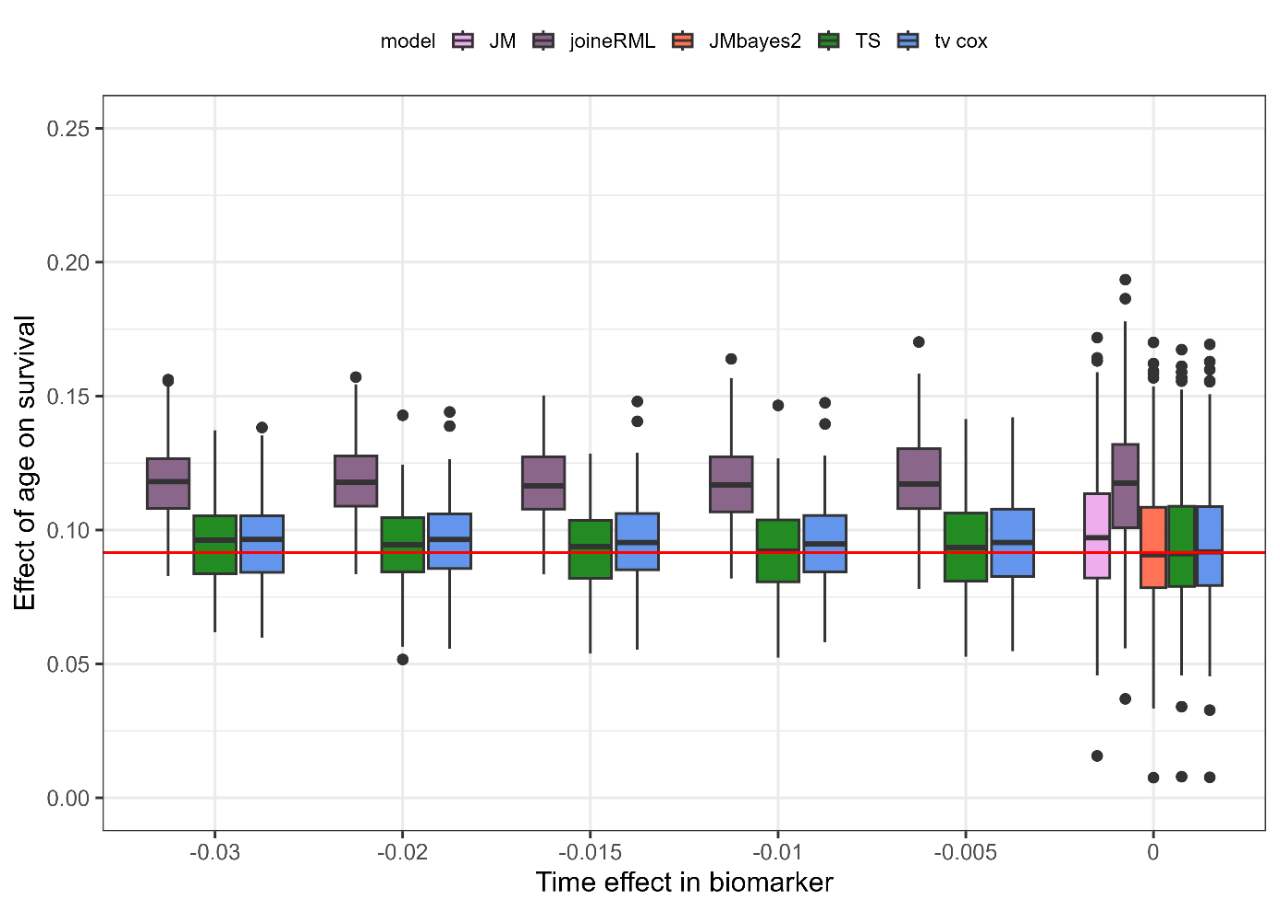


Figure 14 Estimates for the age parameter in survival model (y axis) while varying the time effect in the longitudinal submodel (x axis). tv cox, time-varying Cox proportional-hazards regression; TS, two-stage approach; JM and joineRML, frequentist joint models; JMbayes2, Bayesian joint model. Based on 200 simulations.


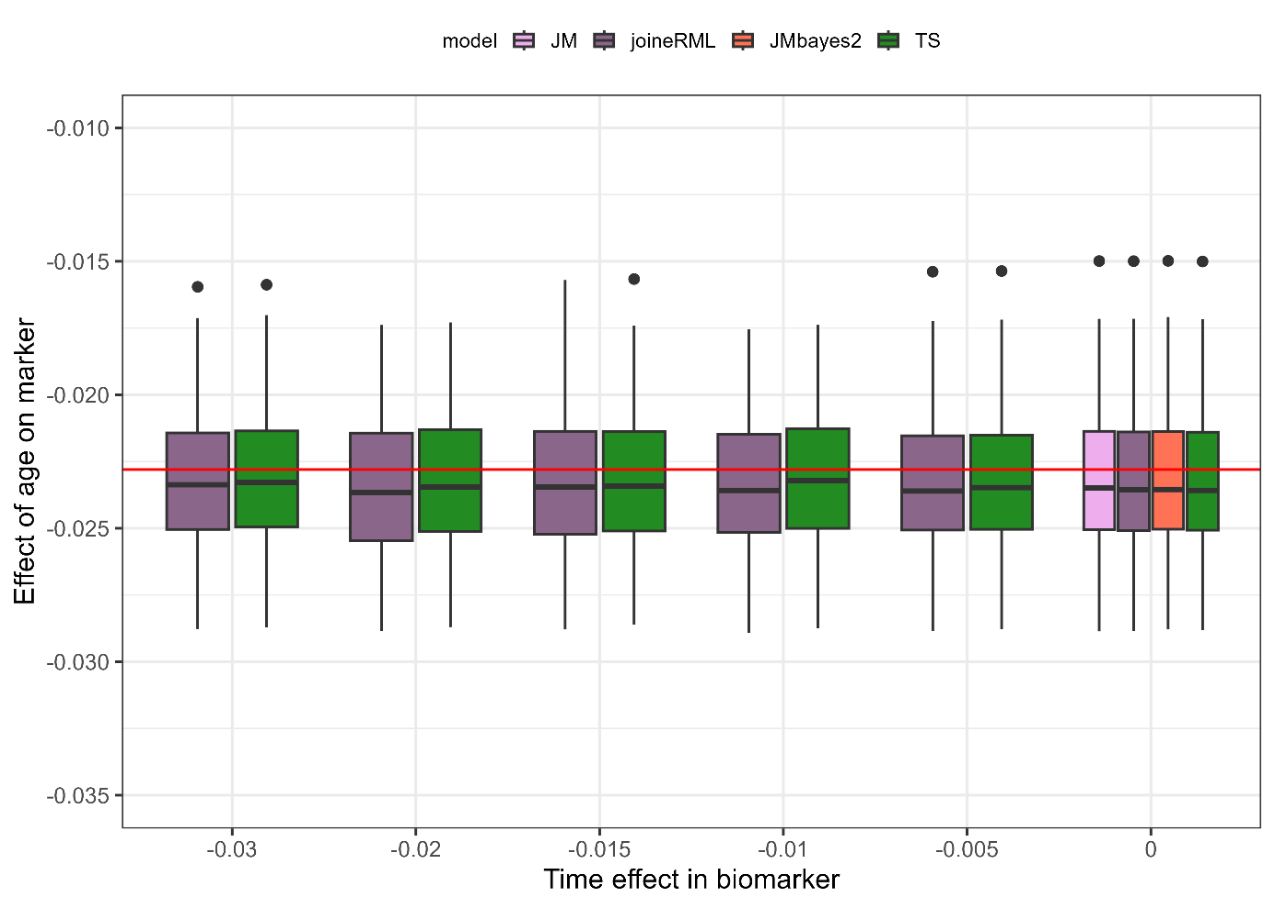


Figure 15 Estimates for the age parameter in longitudinal model (y axis) while varying the time effect in the longitudinal submodel (x axis). tv cox, time-varying Cox proportional-hazards regression; TS, two-stage approach; JM and joineRML, frequentist joint models; JMbayes2, Bayesian joint model. Based on 200 simulations.


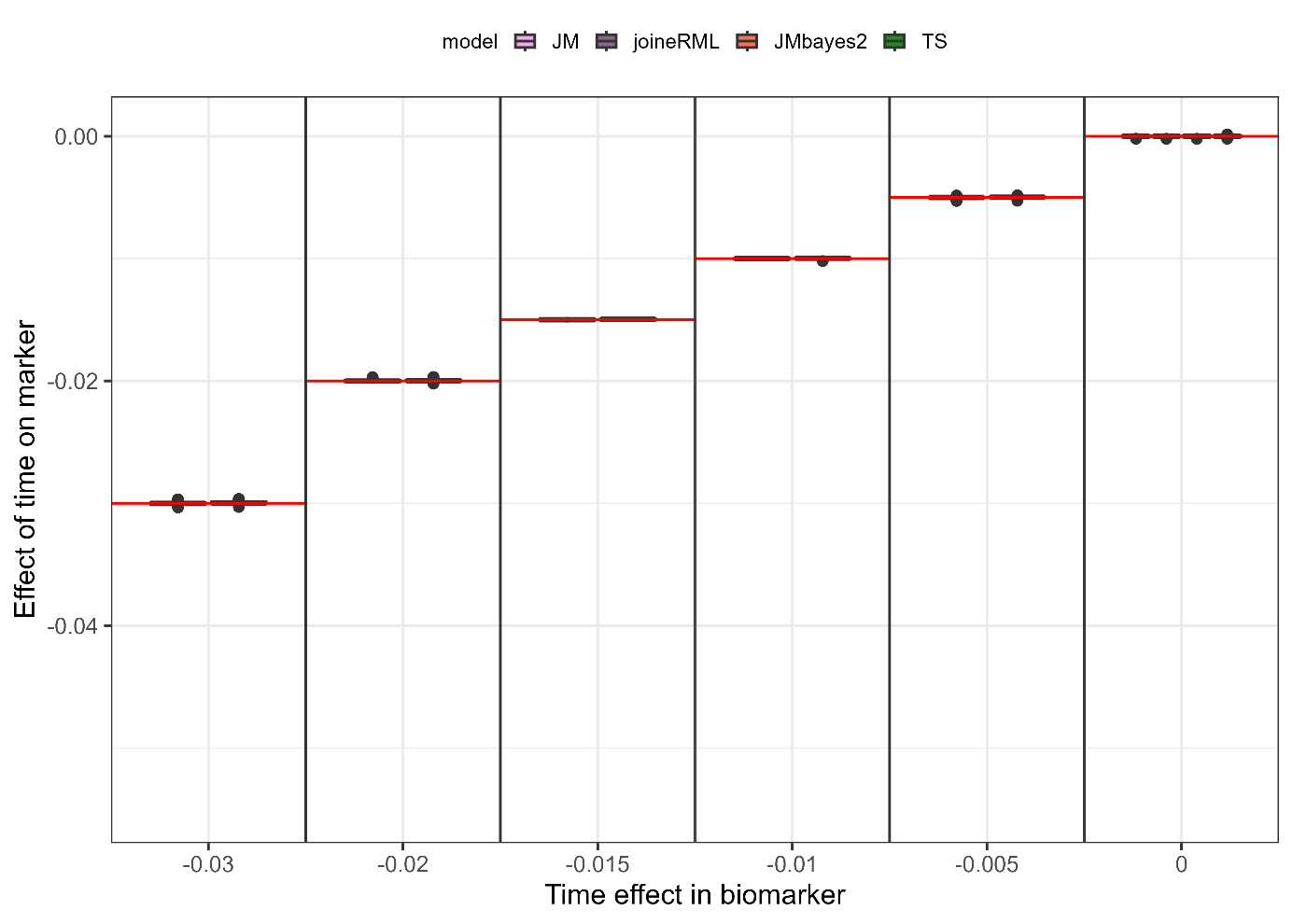


Figure 16 Estimates for the time parameter in longitudinal model (y axis) while varying the time effect in the longitudinal model (x axis). tv cox, time-varying Cox proportional-hazards regression; TS, two-stage approach; JM and joineRML, frequentist joint models; JMbayes2, Bayesian joint model. Based on 200 simulations.

**Setting 5a**


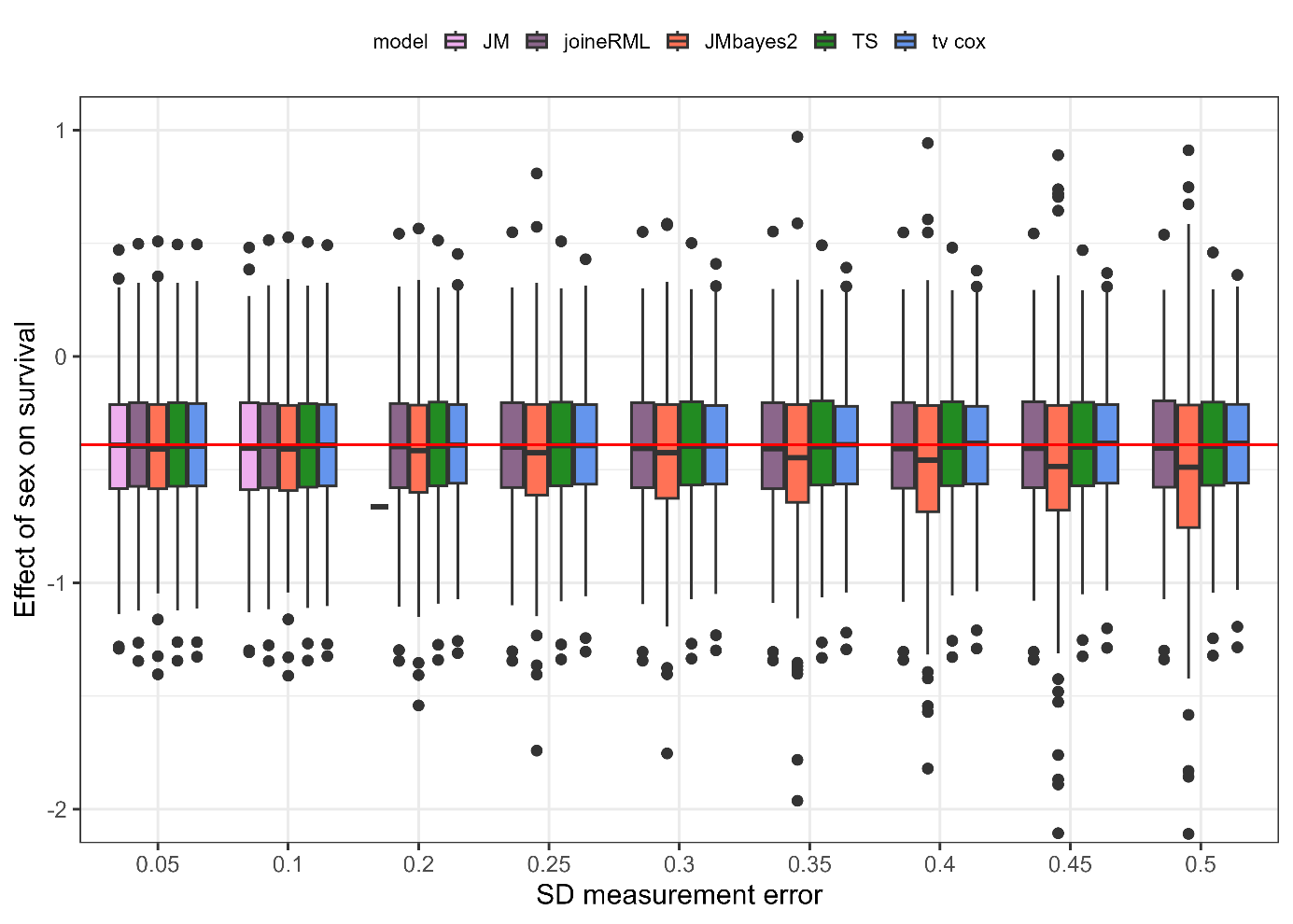


Figure 17 Estimates for the sex parameter in survival model (y axis) while varying the standard deviation of the measurement error (x axis). tv cox, time-varying Cox proportional-hazards regression; TS, two-stage approach; JM and joineRML, frequentist joint models; JMbayes2, Bayesian joint model. Based on 200 simulations


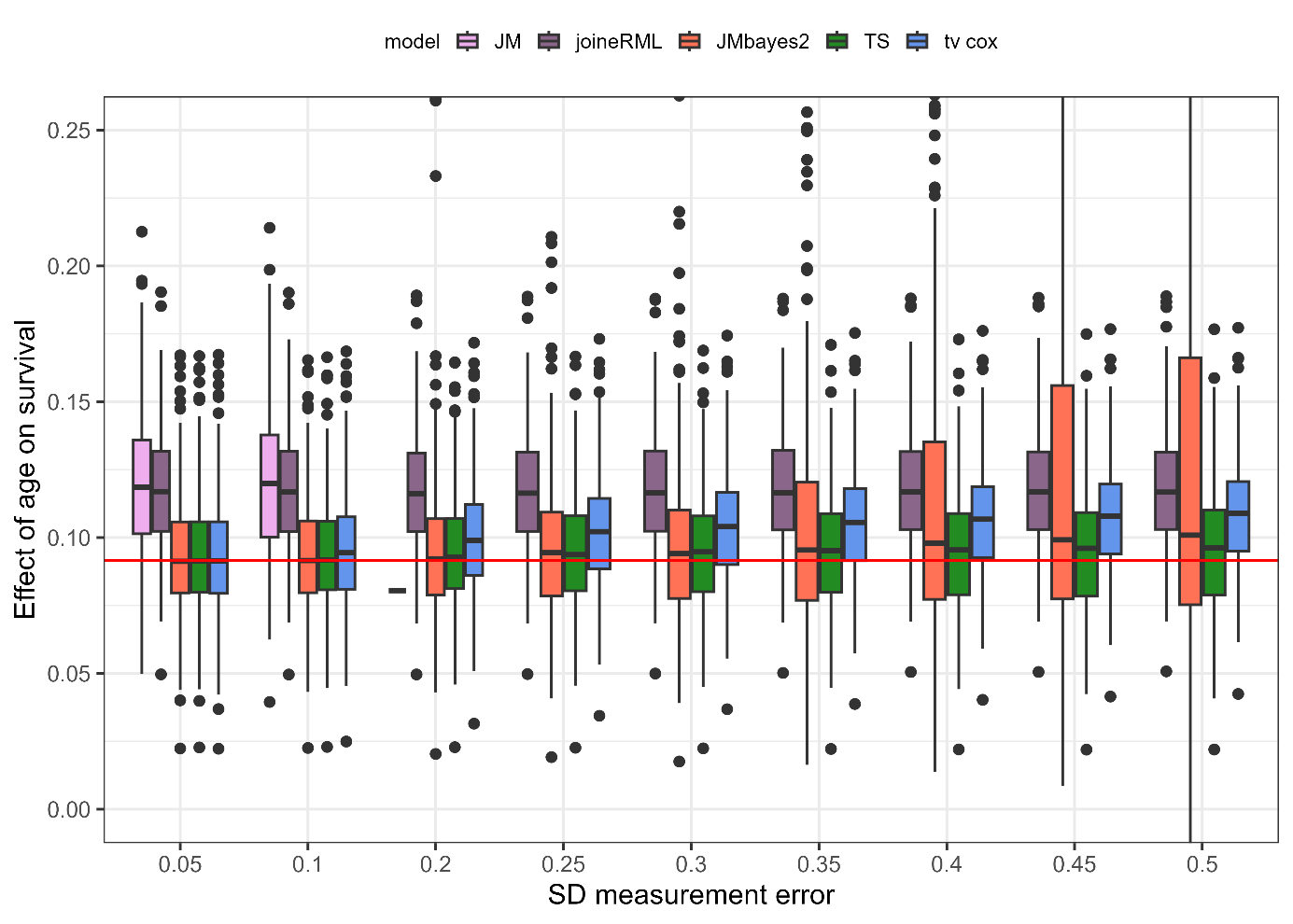


Figure 18 Estimates for the age parameter in survival model (y axis) while varying standard deviation of the measurement error (x axis). tv cox, time-varying Cox proportional-hazards regression; TS, two-stage approach; JM and joineRML, frequentist joint models; JMbayes2, Bayesian joint model. Based on 200 simulations


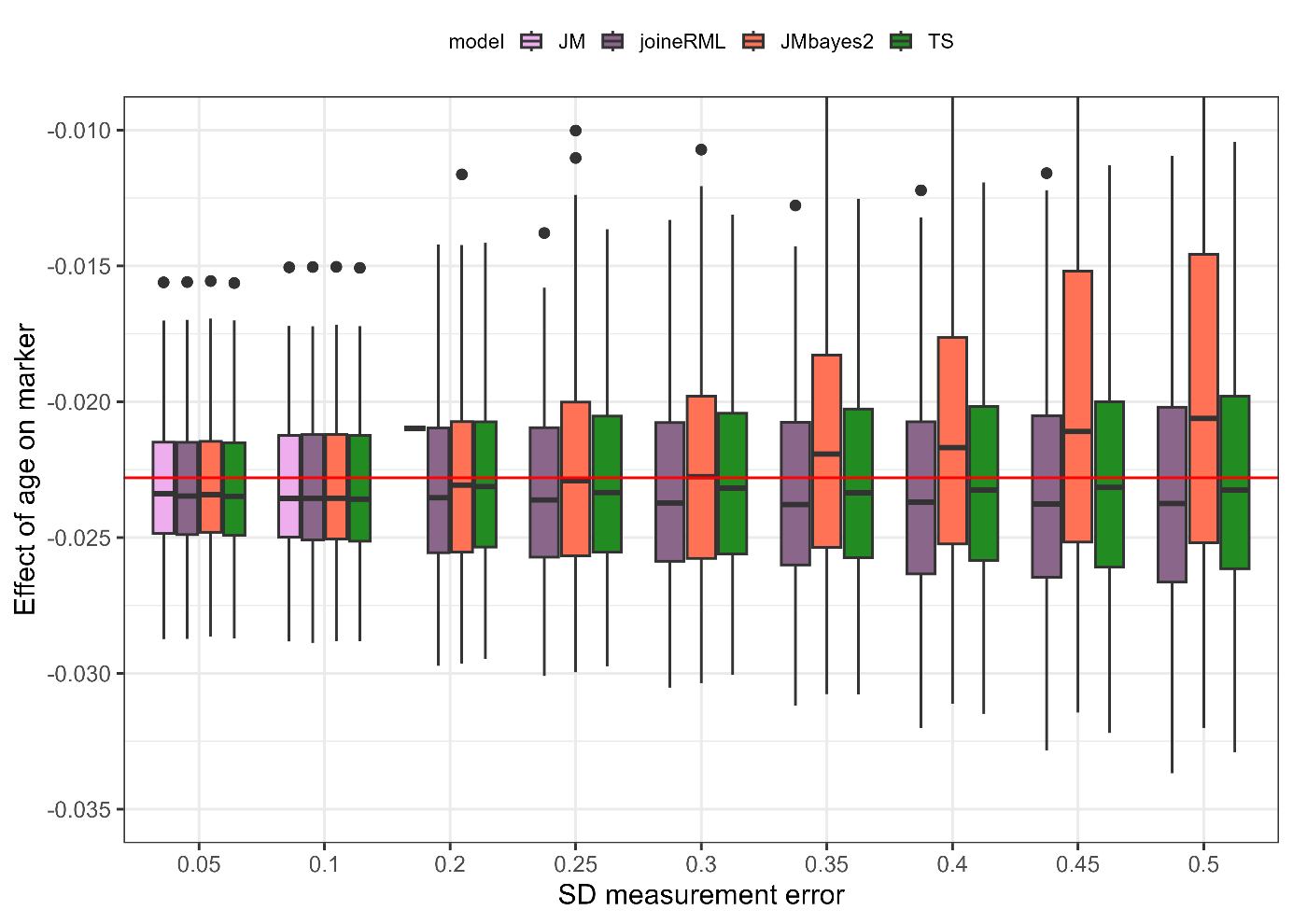


Figure 19 Estimates for the age parameter in longitudinal model (y axis) while varying the standard deviation of the measurement error (x axis). tv cox, time-varying Cox proportional-hazards regression; TS, two-stage approach; JM and joineRML, frequentist joint models; JMbayes2, Bayesian joint model. Based on 200 simulations.


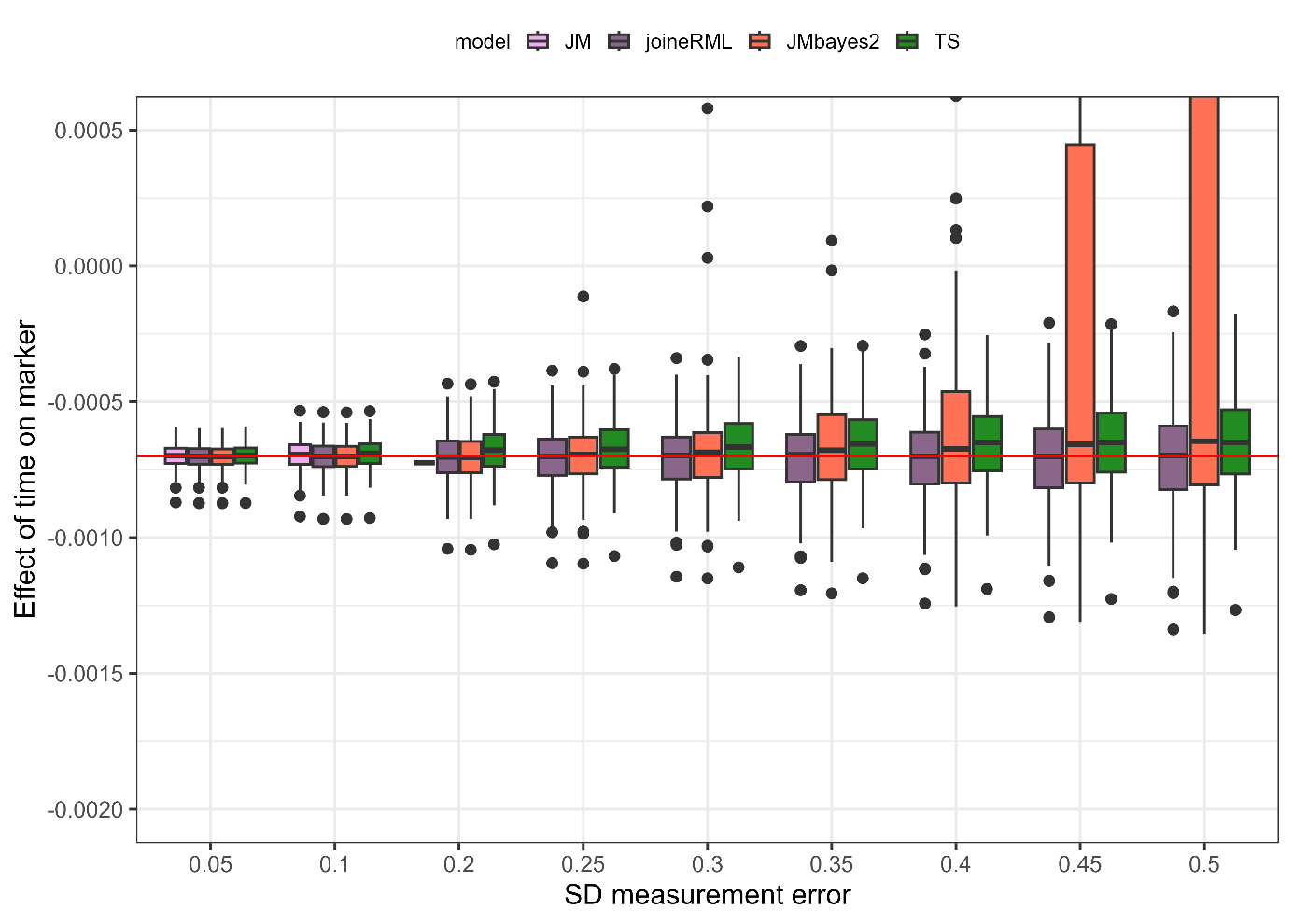


Figure 20 Estimates for the time parameter in longitudinal model (y axis) while varying the standard deviation of the measurement error (x axis). tv cox, time-varying Cox proportional-hazards regression; TS, two-stage approach; JM and joineRML, frequentist joint models; JMbayes2, Bayesian joint model. Based on 200 simulations.

## Nonlinear trajectory (setting b)

**Setting 2b**


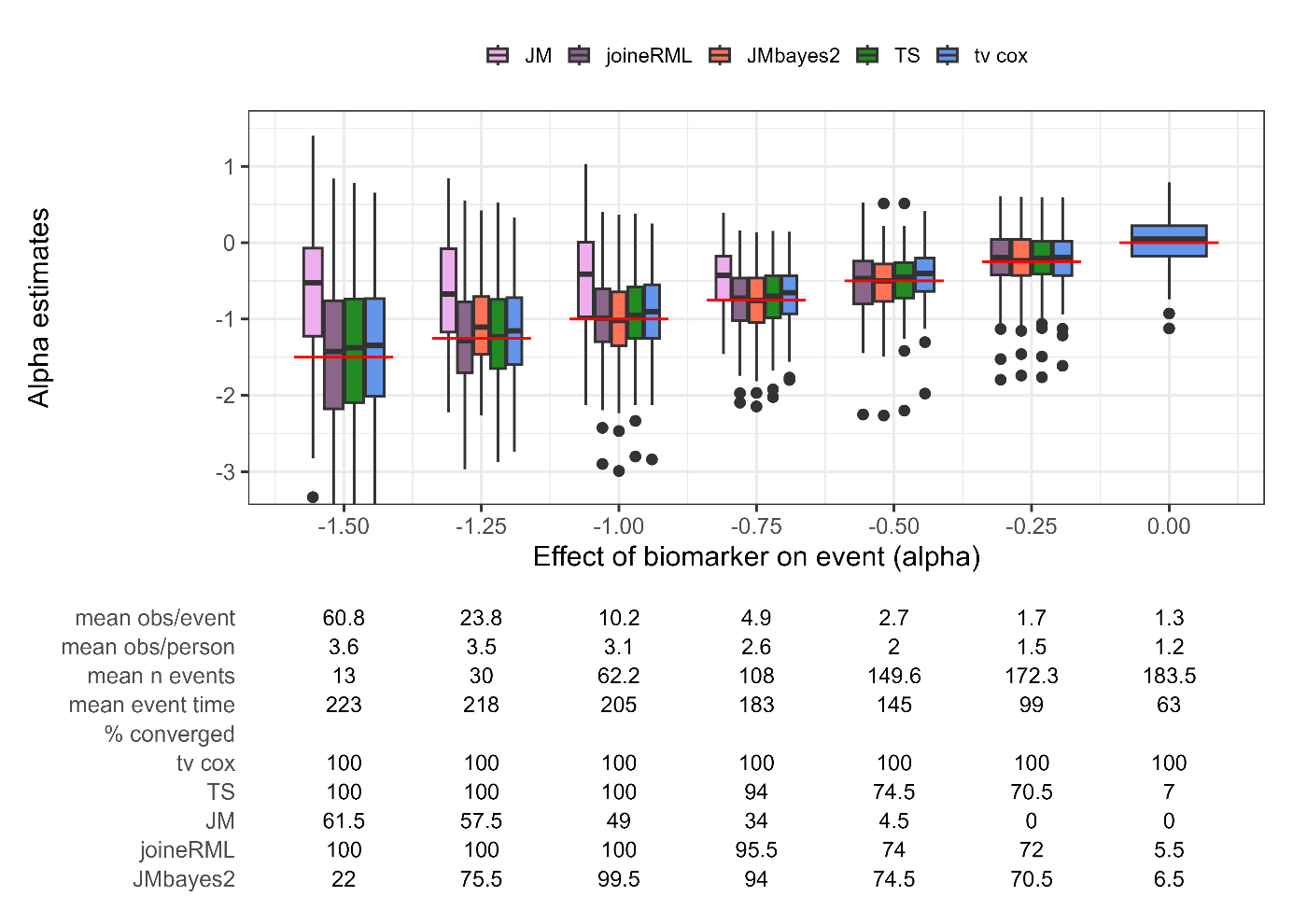


Figure 21. Estimates for the association parameter $\alpha$ (y axis) while varying association parameter itself (x axis). tv cox, time-varying Cox proportional-hazards regression; TS, two-stage approach; JM and joineRML, frequentist joint models; JMbayes2, Bayesian joint model. % converged, percentage of converged models out of 200 simulations; obs, number of observations; n event, number of events. Based on 200 simulations

**Setting 3b**


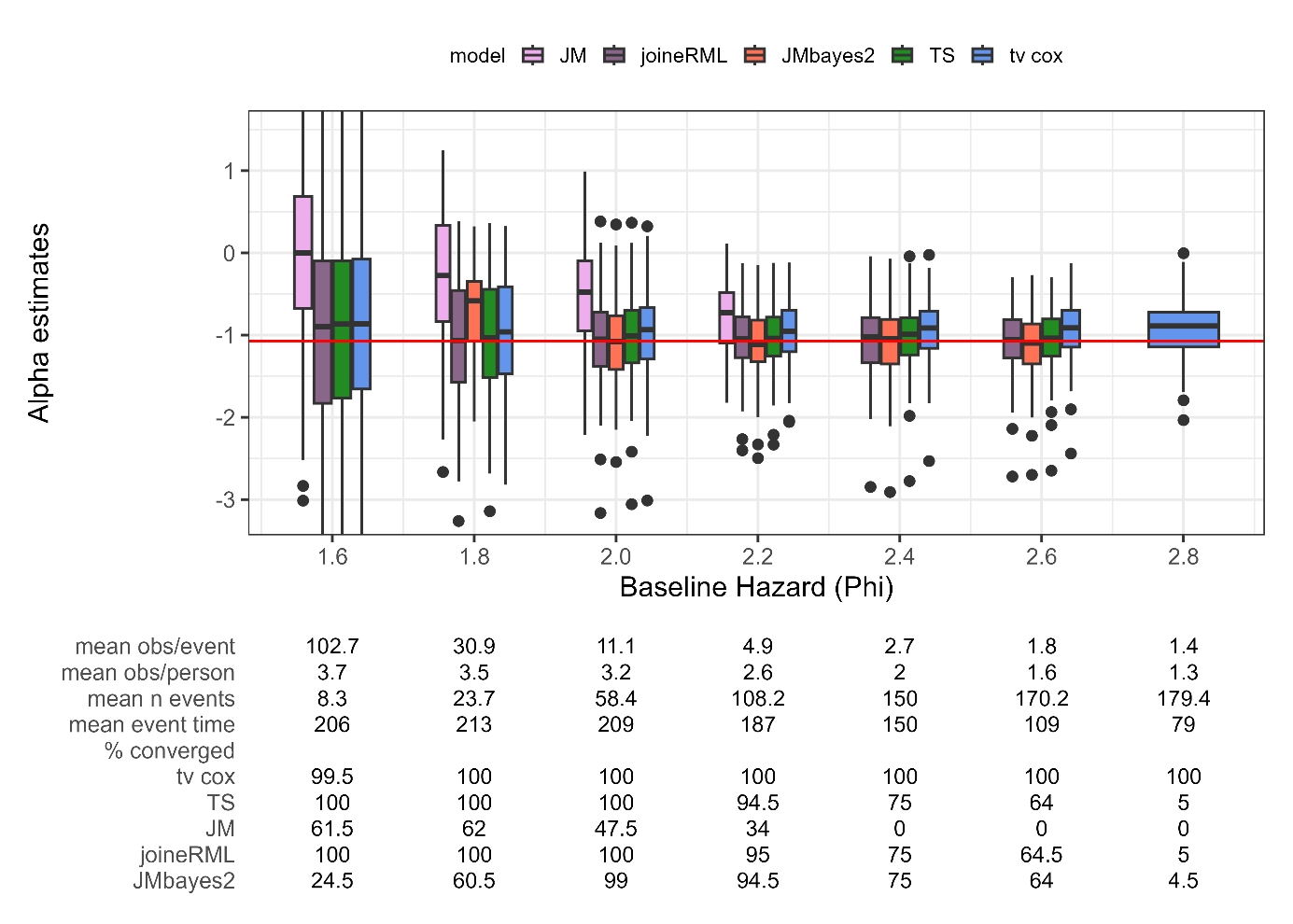


Figure 22. Estimates for the association parameter $\alpha$ (y axis) while varying parameter $\phi$ in the Weibull baseline hazard (x axis). tv cox, time-varying Cox proportional-hazards regression; TS, two-stage approach; JM and joineRML, frequentist joint models; JMbayes2, Bayesian joint model. % converged, percentage of converged models out of 200 simulations; obs, number of observations; n event, number of events. Based on 200 simulations

## Sensitivity JMbayes2

**Setting 2a**


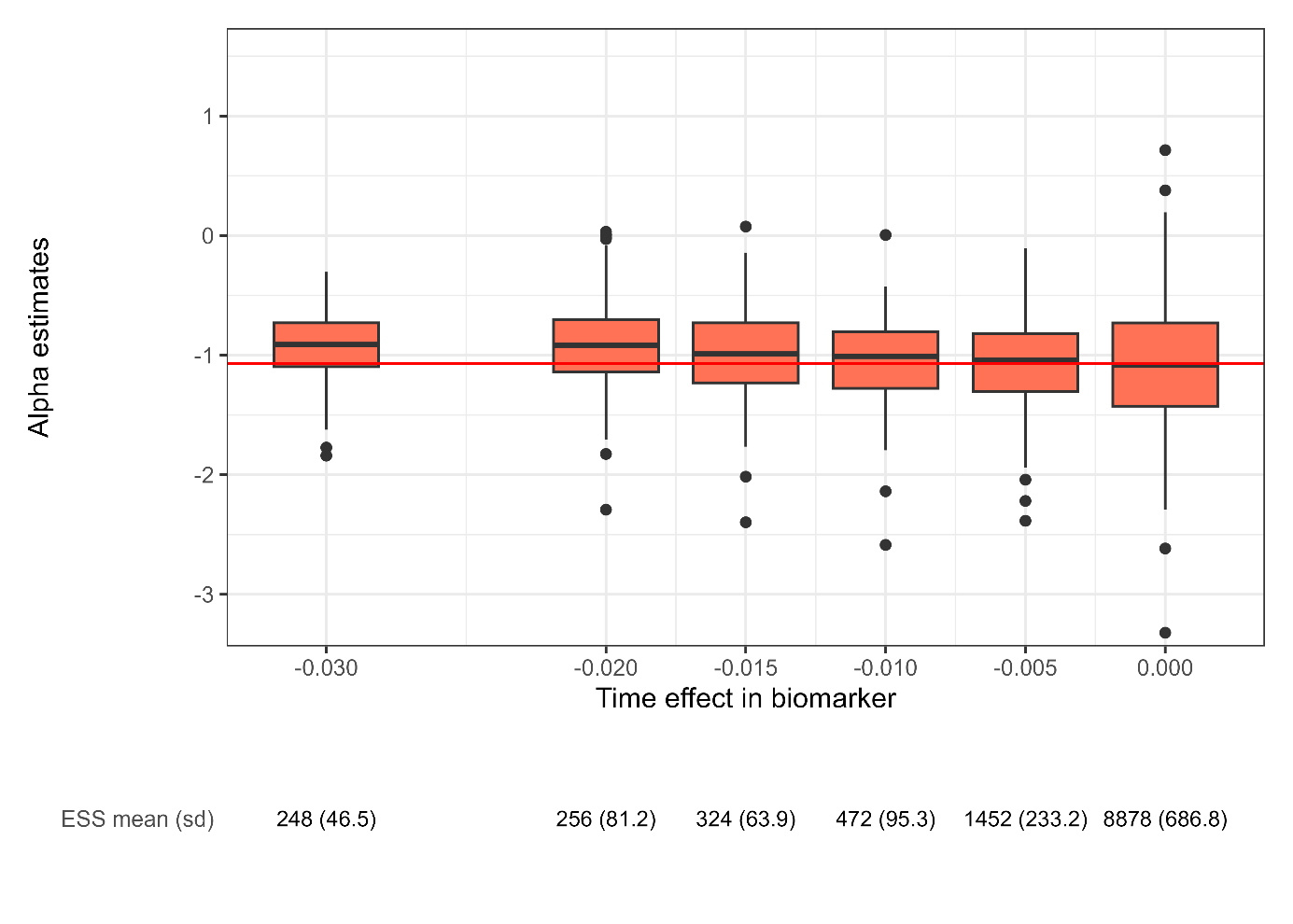


Figure 23. Estimates for the association parameter $\alpha$ (y axis) while varying the time effect in the longitudinal model (x axis) for JMbayes2; using 100,000 MCMC iterations plus burn-in of 5,000 and thinning of 20 on three parallel chains, plus informative prior distribution. Based on 200 simulations

## Sensitivity JM package

**Setting 1a**


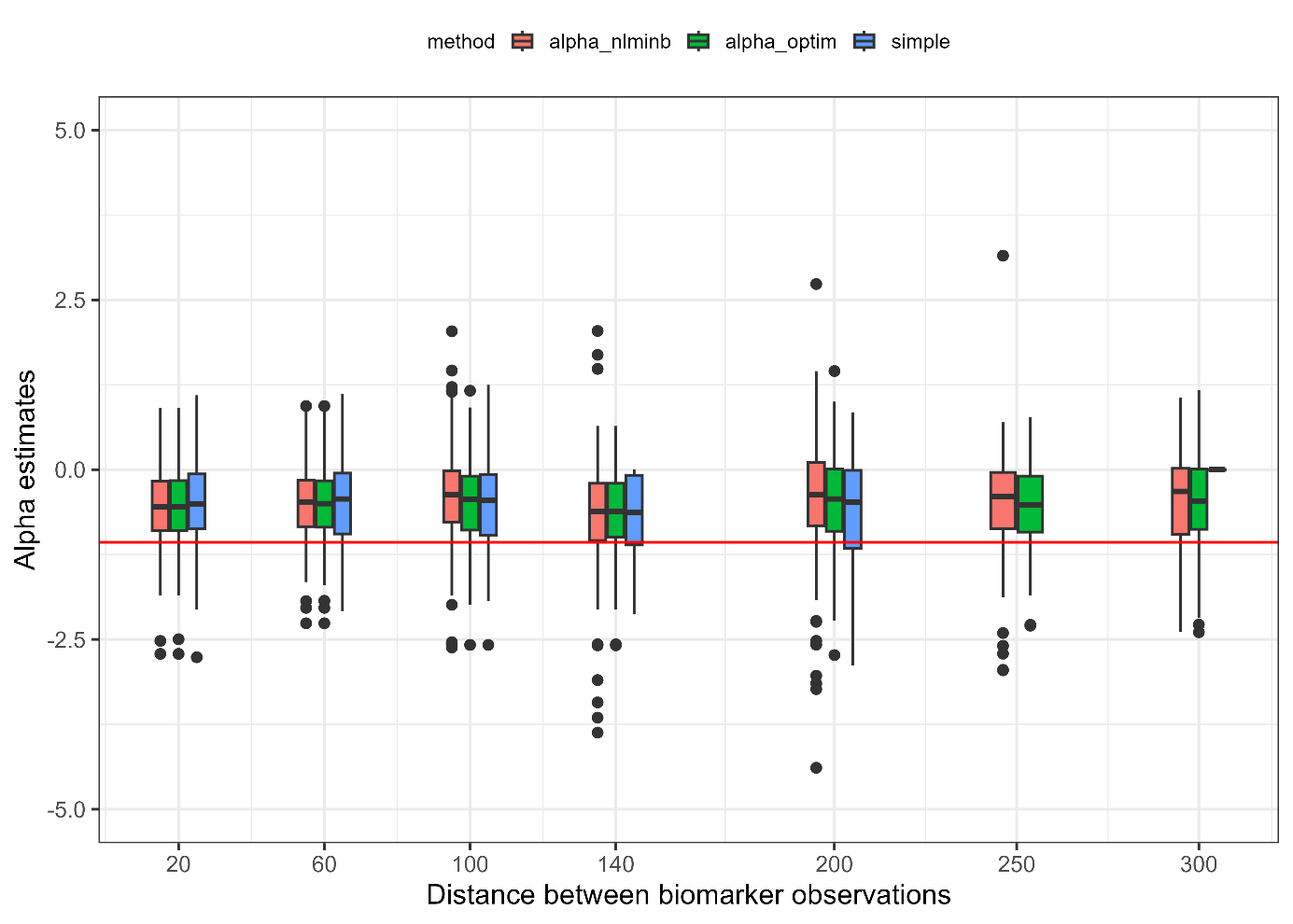


Figure 24. Estimates for the association parameter $\alpha$ (y axis) while varying the distance between longitudinal observation time points (x axis) for JM package. alpha_nlminb uses nlminb optimizer with iter.EM = 200, iter.qN = 500; alpha_optim uses default JM optimizer optim with iter.EM = , iter.gN = 500; simple uses default optimizer optim with a simplified random effects structure modelling only random intercepts (iter.EM = 200, iter.qn = 500). Based on 200 simulations

**Setting 2a**


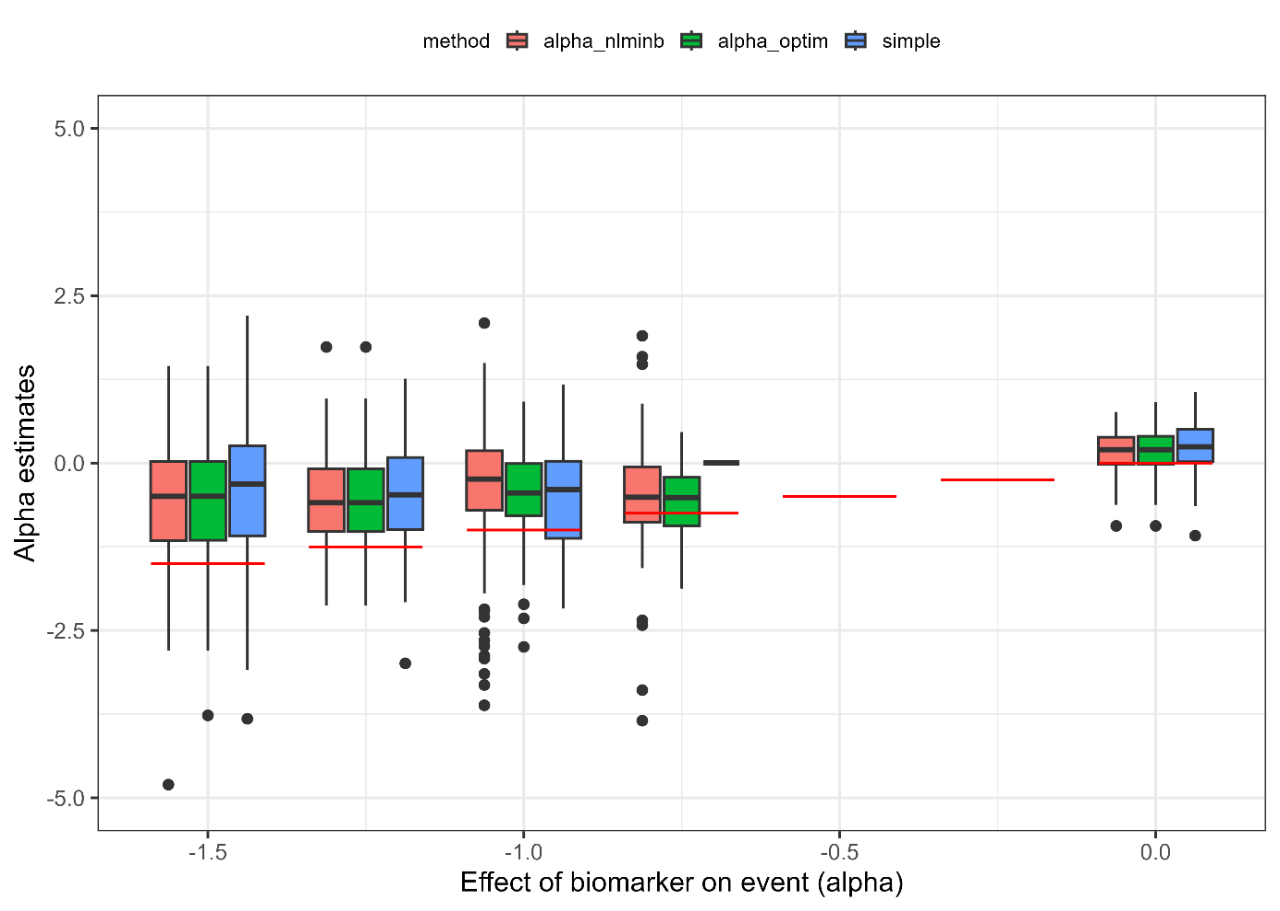


Figure 25. Estimates for the association parameter $\alpha$ (y axis) while varying the association parameter itself (x axis) for JM package. alpha_nlminb uses nlminb optimizer with iter.EM = 200, iter.qN = 500; alpha_optim uses default JM optimizer optim with iter.EM = 200, iter.gN = 500; simple uses default optimizer optim with a simplified random effects structure modelling only random intercepts (iter.EM = 200, iter.qn = 500). Based on 200 simulations

**Setting 3a**


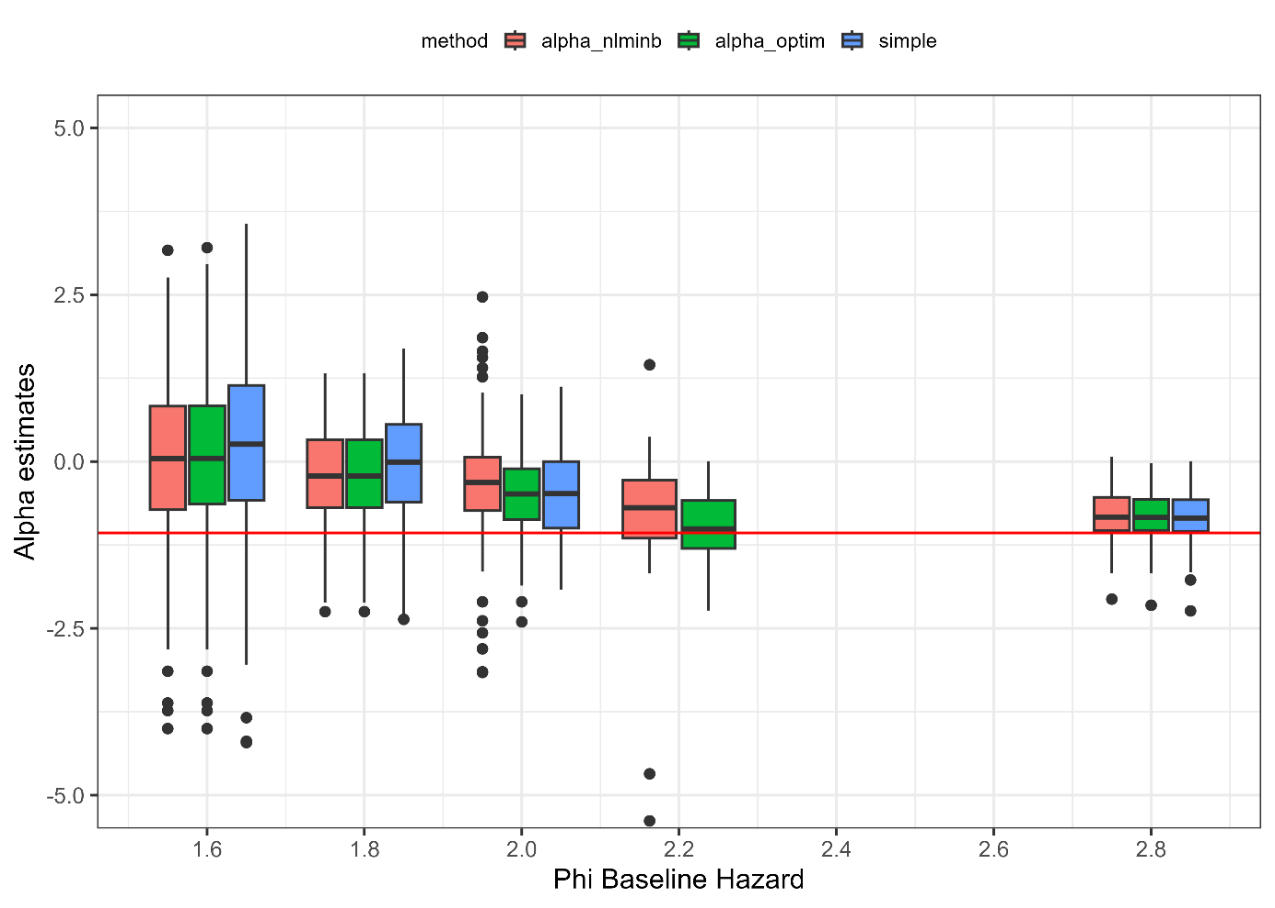


Figure 26. Estimates for the association parameter $\alpha$ (y axis) while varying the $\phi$ in the Weibull baseline hazard (x axis) for JM package. alpha_nlminb uses nlminb optimizer with iter.EM = 200, iter.qN = 500; alpha_optim uses default JM optimizer optim with iter.EM = 200, iter.gN = 500; simple uses default optimizer optim with a simplified random effects structure modelling only random intercepts (iter.EM = 200, iter.qn = 500). Based on 200 simulations.

## Unconditional results

**Setting 1a**


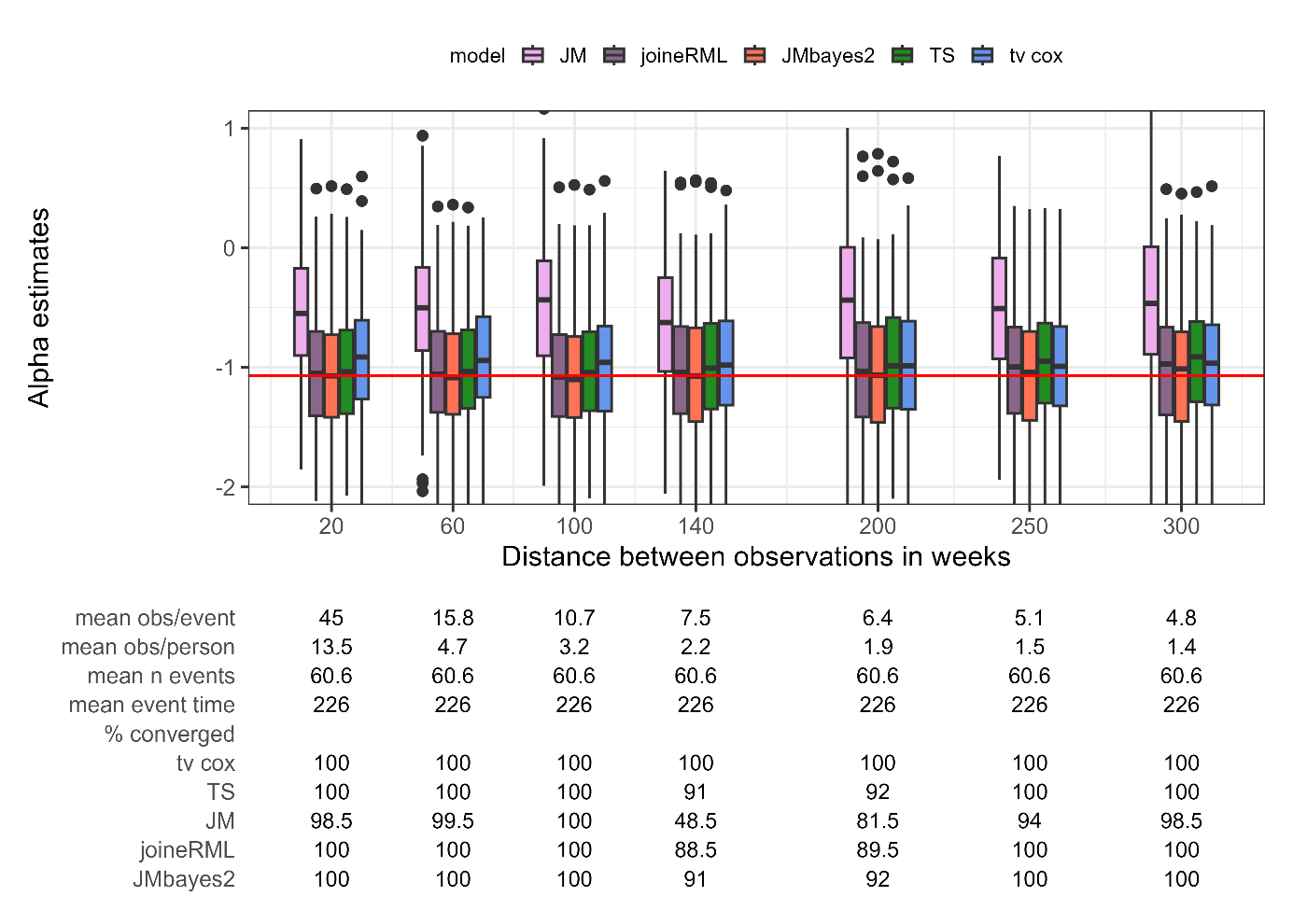


Figure 27. Converged and unconverged stimates for the association parameter α (y axis) while varying the distance between longitudinal observations of the biomarker and thus their density (x axis). tv cox, time-varying Cox proportional-hazards regression; TS, two-stage approach; JM and joineRML, frequentist joint models; JMbayes2, Bayesian joint model. % converged, percentage of converged models out of 200 simulations; obs, number of observations; n event, number of events. Based on 200 simulations.

**Setting 3a**


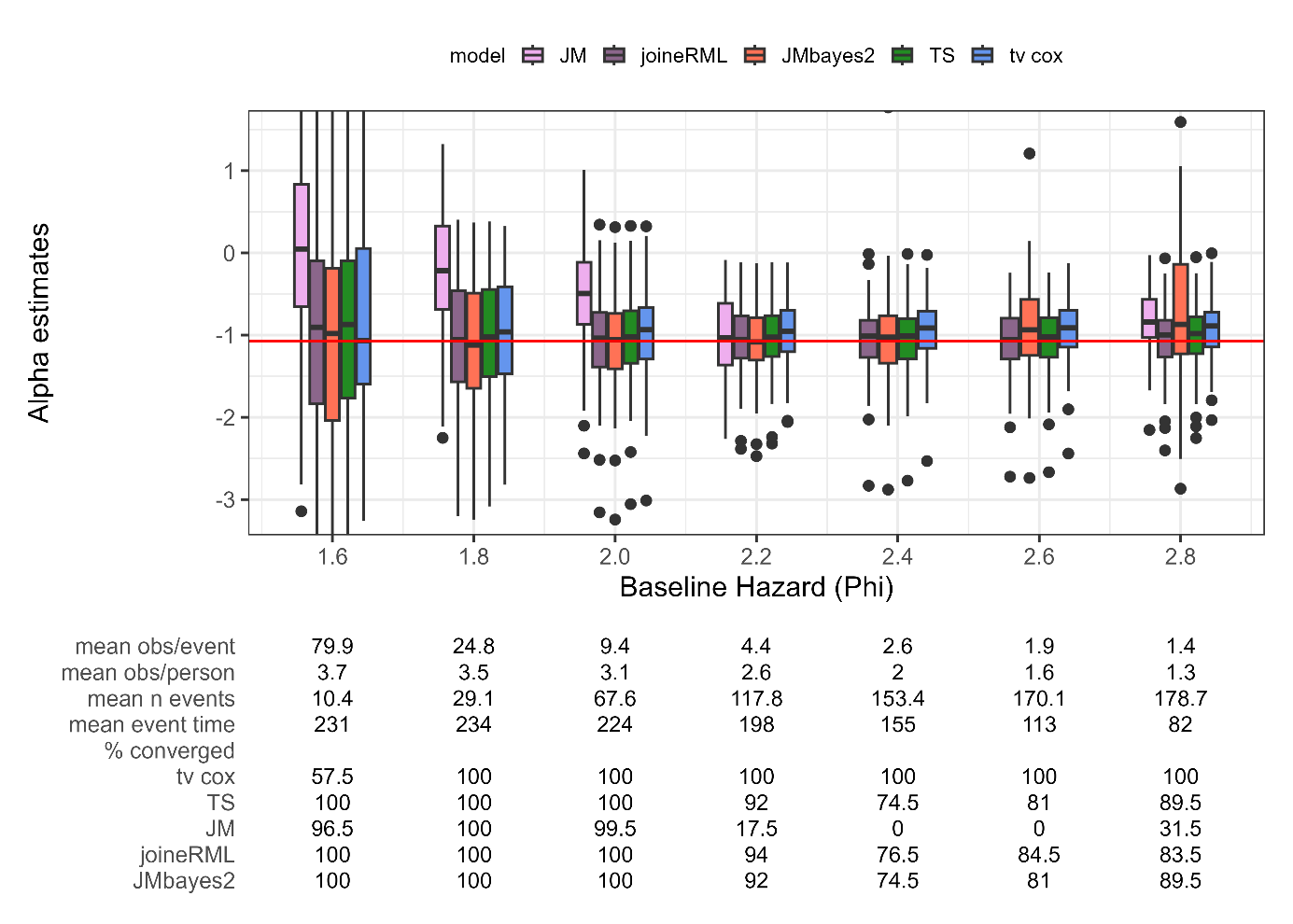


Figure 28. Converged and unconverged estimates for the association parameter α (y axis) while varying the baseline hazard (x axis). Red horizontal line indicates true parameter value. tv cox, time-varying Cox proportional-hazards regression; TS, two-stage approach; JM and joineRML, frequentist joint models; JMbayes2, Bayesian joint model. % converged, percentage of converged models out of 200 simulations; obs, number of observations; n event, number of events. Based on 200 simulations

**Setting 4a**


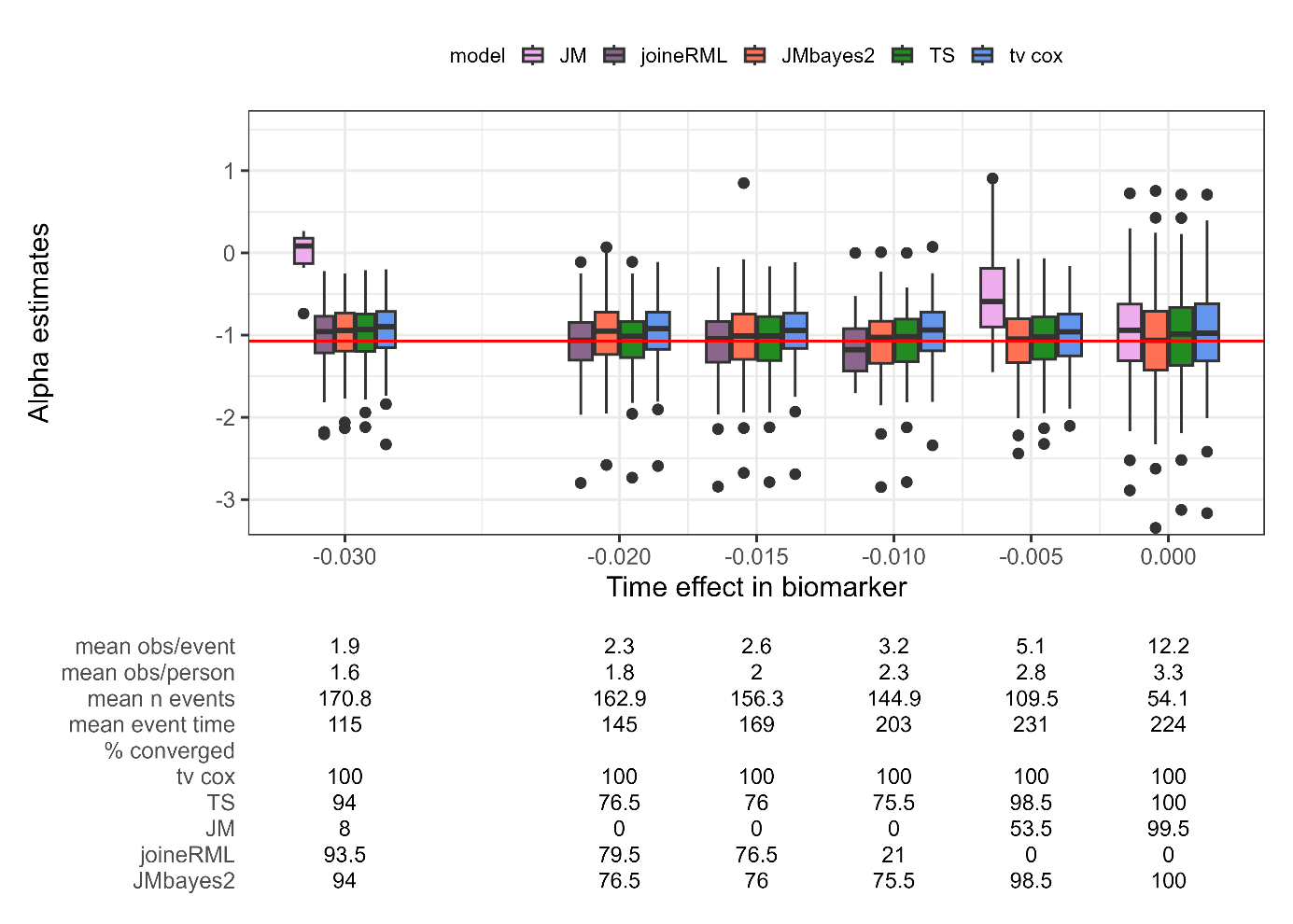


Figure29. Converged and unconverged estimates for the association parameter α (y axis) while varying the time effect in the longitudinal submodel (x axis). Red horizontal line indicates true parameter value. tv cox, time-varying Cox proportional-hazards regression; TS, two-stage approach; JM and joineRML, frequentist joint models; JMbayes2, Bayesian joint model. % converged, percentage of converged models out of 200 simulations; obs, number of observations; n event, number of events. Based on 200 simulations.

**Setting 5a**


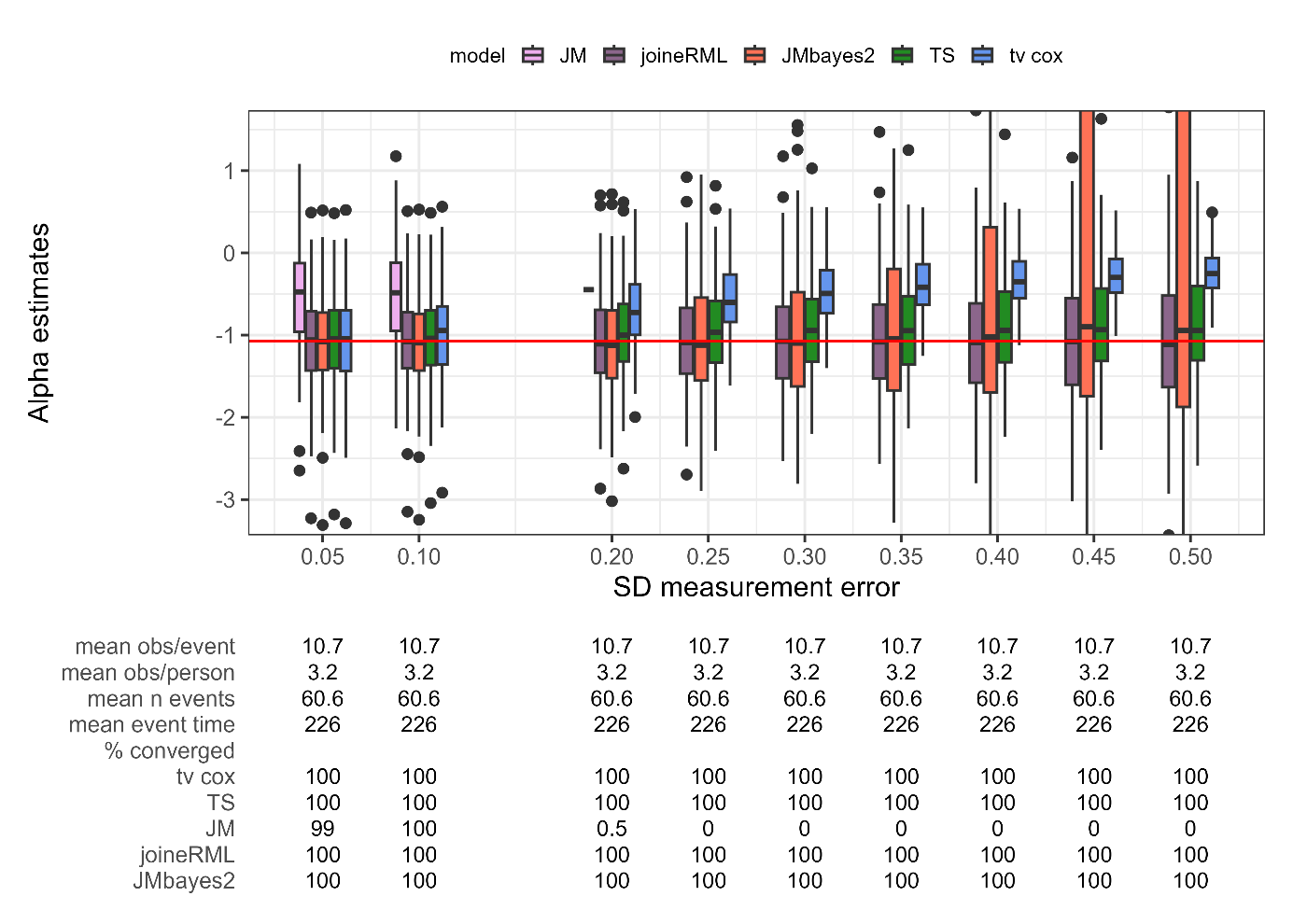


Figure 30. Converged and unconverged Estimates for the association parameter α (y axis) while varying the standard deviation of the measurement error (x axis). Red horizontal line indicates true parameter value. tv cox, time-varying Cox proportional-hazards regression; TS, two-stage approach; JM and joineRML, frequentist joint models; JMbayes2, Bayesian joint model. % converged, percentage of converged models out of 200 simulations; obs, number of observations; n event, number of events. Based on 200 simulations.

## Only converged settings

**Setting 1a**


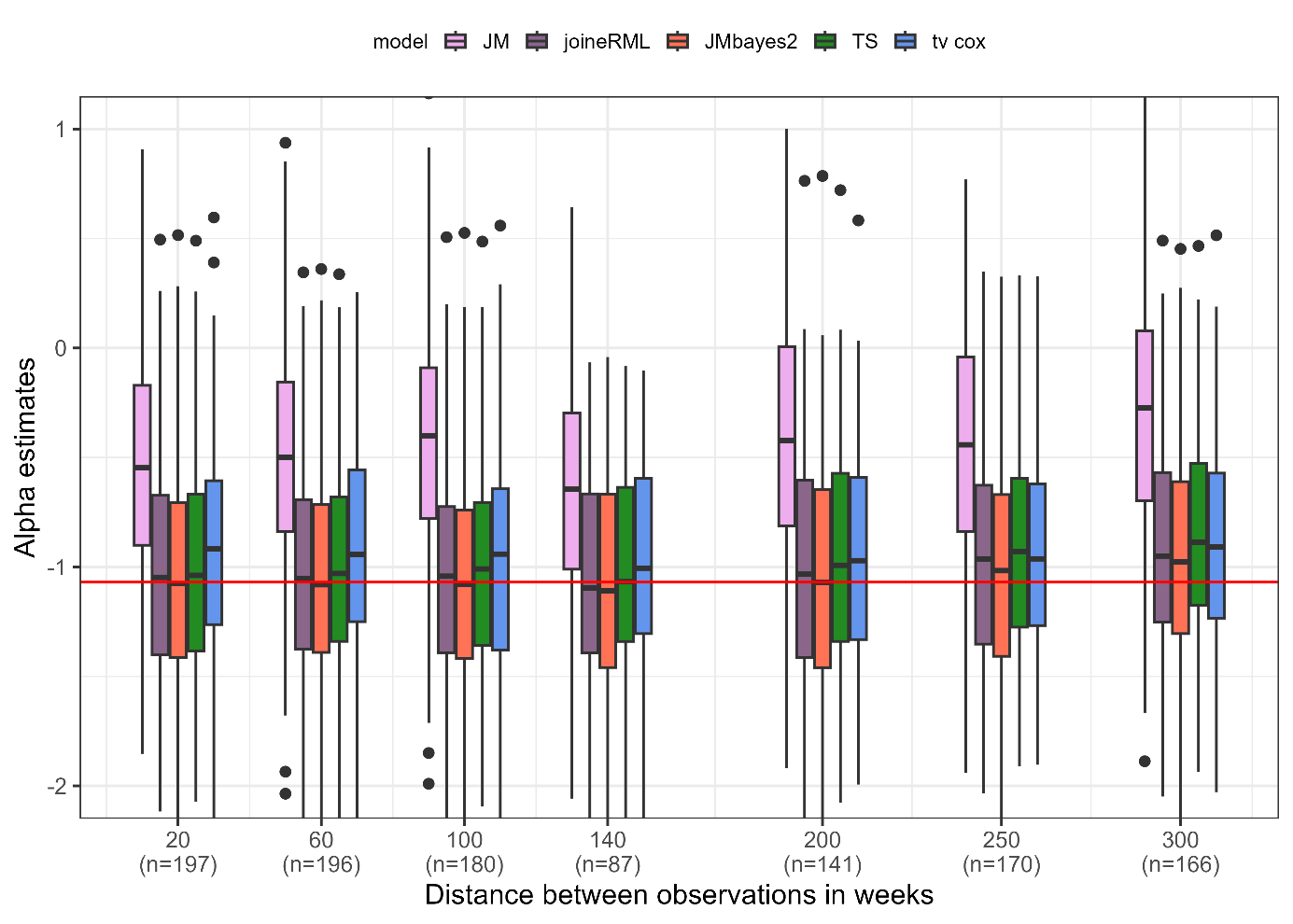


Figure 31. Settings where all models converged. Estimates for the association parameter α (y axis) while varying the distance between longitudinal observations of the biomarker and thus their density (x axis). tv cox, time-varying Cox proportional-hazards regression; TS, two-stage approach; JM and joineRML, frequentist joint models; JMbayes2, Bayesian joint model. Based on 200 simulations.

**Setting 2a**


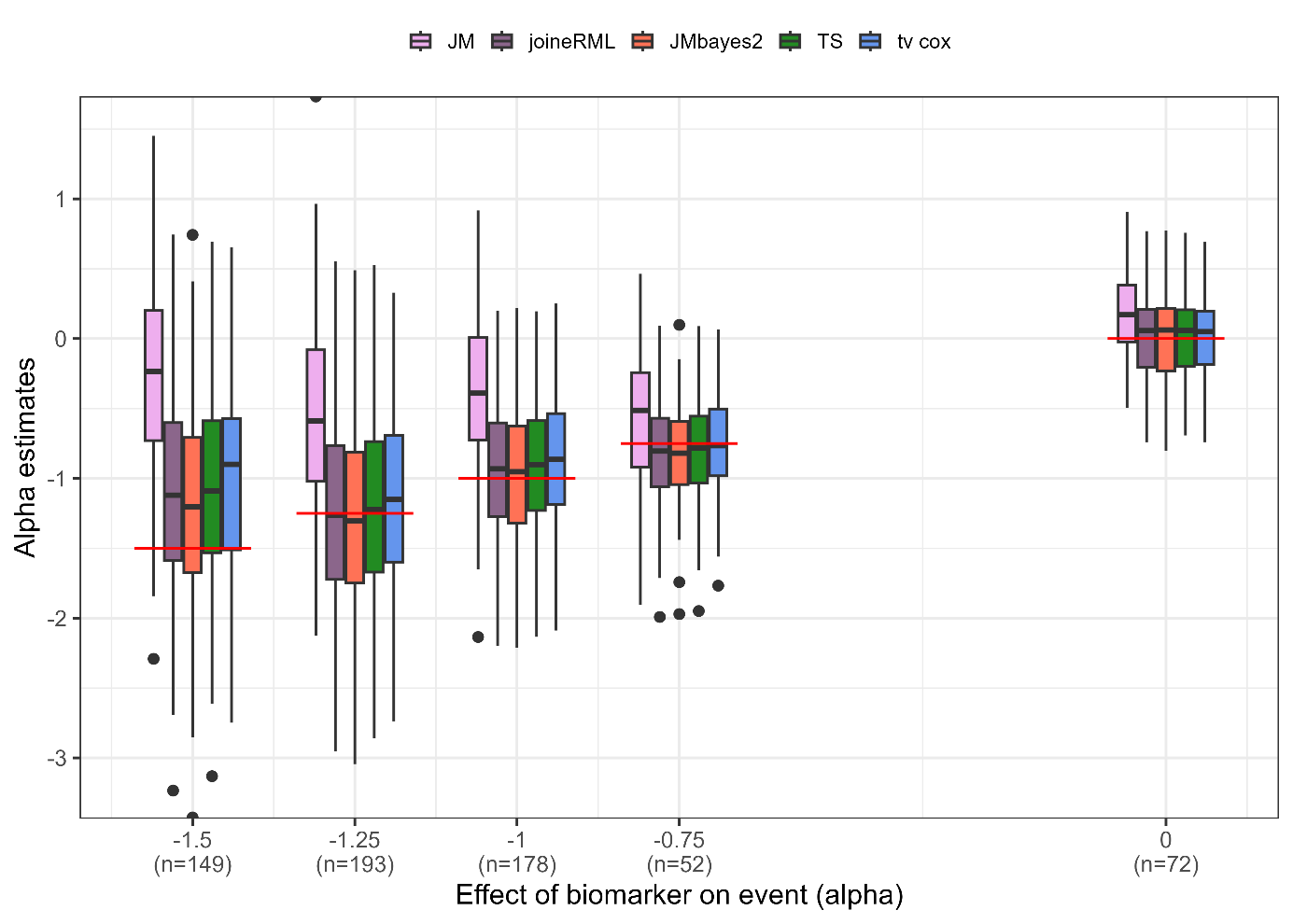


Figure 32. Settings where all models converged. Estimates for the association parameter α (y axis) while varying the association parameter itself (x axis). tv cox, time-varying Cox proportional-hazards regression; TS, two-stage approach; JM and joineRML, frequentist joint models; JMbayes2, Bayesian joint model. Based on 200 simulations.

**Setting 3a**


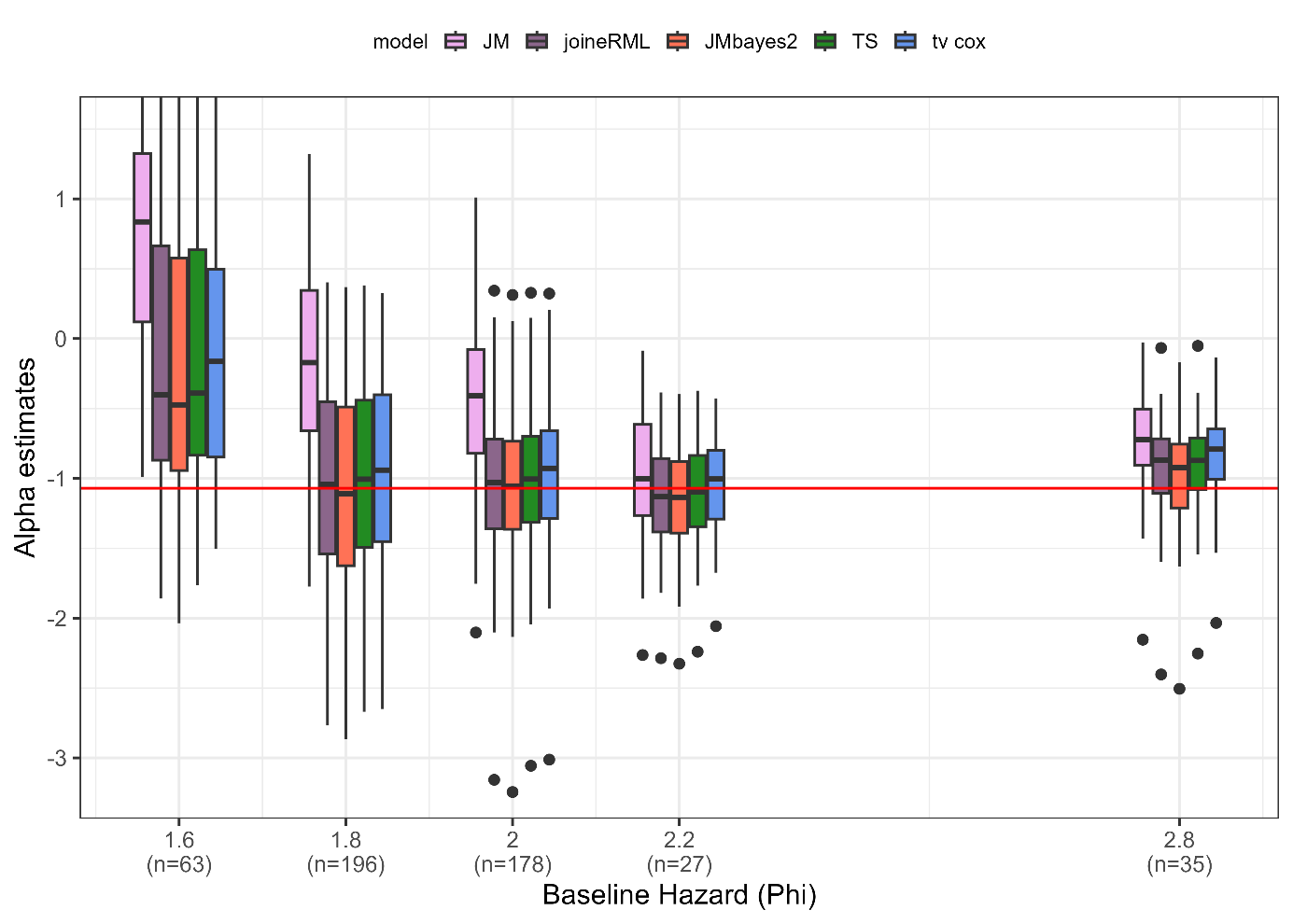


Figure 33. Settings where all models converged. Estimates for the association parameter α (y axis) while varying $\phi$ in the Weibull baseline hazard (x axis). tv cox, time-varying Cox proportional-hazards regression; TS, two-stage approach; JM and joineRML, frequentist joint models; JMbayes2, Bayesian joint model. Based on 200 simulations.

**Setting 4a**


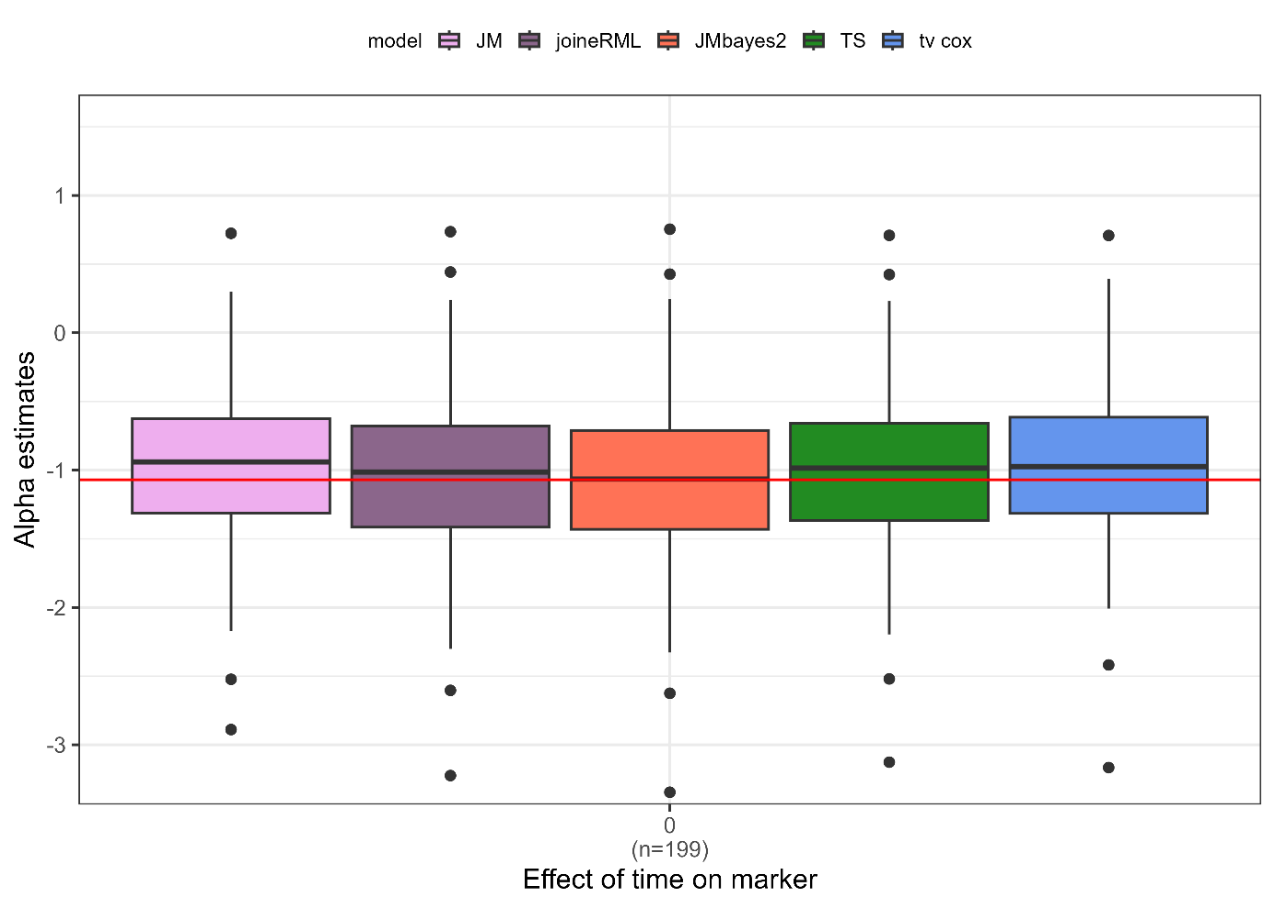


Figure 34. Settings where all models converged. Estimates for the association parameter α (y axis) while varying the time effect in the longitudinal submodel (x axis). tv cox, time-varying Cox proportional-hazards regression; TS, two-stage approach; JM and joineRML, frequentist joint models; JMbayes2, Bayesian joint model. Based on 200 simulations.

**Setting 5a**


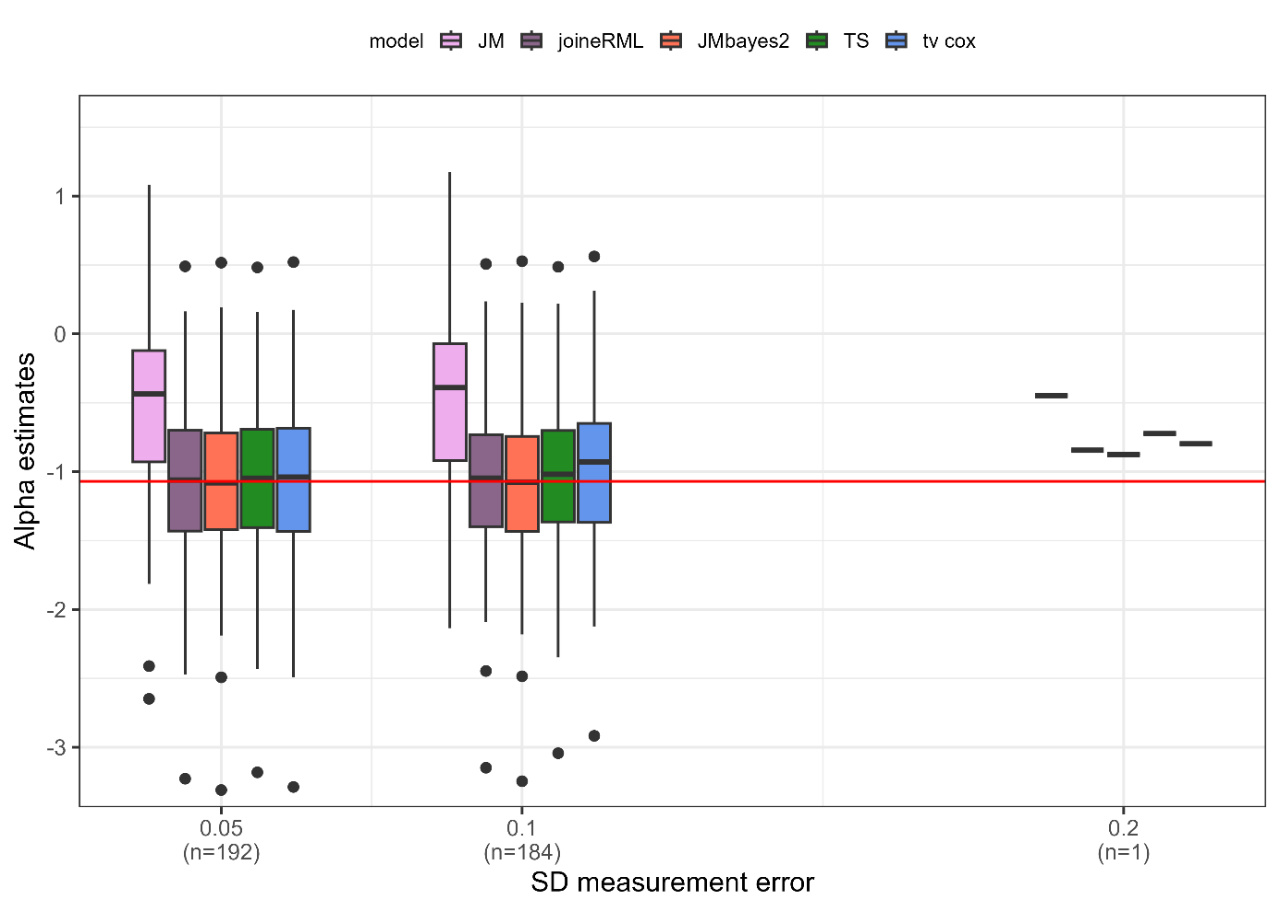


Figure 35. Settings where all models converged. Estimates for the association parameter α (y axis) while varying the standard deviation in the measurement error (x axis). tv cox, time-varying Cox proportional-hazards regression; TS, two-stage approach; JM and joineRML, frequentist joint models; JMbayes2, Bayesian joint model. Based on 200 simulations.

# Computation times

| **Setting 1a** |  |  |  |  |  |  |  |
| --- | --- | --- | --- | --- | --- | --- | --- |
| distance observations | *20* | *60* | *100* | *140* | *200* | *250* | *300* |
| JM | 2.9 | 4.6 | 4.9 | 10.0 | 8.5 | 11.5 | 10.7 |
|  | 18.5 | 17.8 | 19.6 | 16.2 | 16.5 | 17.8 | 17.9 |
| JMbayes2 | 729.3 | 690.9 | 649.6 | 591.6 | 590 | 639 | 638.6 |
| TS | 0.2 | 0.2 | 0.2 | 0.5 | 0.4 | 0.2 | 0.2 |
| tv cox | 0.1 | 0.1 | 0.1 | 0.1 | 0.1 | 0.1 | 0.1 |
|  |  |  |  |  |  |  |  |
| **Setting 2a** |  |  |  |  |  |  |  |
| alpha | *-1.5* | *-1.25* | *-1* | *-0.75* | *-0.5* | *-0.25* | *0* |
| JM | 4.4 | 4.5 | 6.4 | 18.1 |  |  | 4.7 |
| joineRML | 10.5 | 14.5 | 19.5 | 25.8 | 24.7 | 25.4 | 29.5 |
| JMbayes2 | 661 | 655 | 653 | 625 | 478 | 511 | 572 |
| TS | 0.1 | 0.1 | 0.2 | 0.3 | 0.4 | 0.4 | 0.4 |
| tv cox | 0.1 | 0.1 | 0.1 | 0.1 | 0.1 | 0.1 | 0.1 |
|  |  |  |  |  |  |  |  |
| **Setting 3a** |  |  |  |  |  |  |  |
| baseline hazard | *1.6* | *1.8* | *2* | *2.2* | *2.4* | *2.6* | *2.8* |
| JM | 4.2 | 2.7 | 4.4 |  |  |  |  |
| joineRML | 9.4 | 13.1 | 18.9 | 23.7 | 23.3 | 27.2 | 28 |
| JMbayes2 | 507 | 501 | 506 | 470 | 380 | 404 | 440 |
| TS | 0.1 | 0.1 | 0.2 | 0.4 | 0.5 | 0.5 | 0.5 |
| tv cox | 0.1 | 0.1 | 0.1 | 0.1 | 0.1 | 0.1 | 0.1 |
|  |  |  |  |  |  |  |  |
| **Setting 4a** |  |  |  |  |  |  |  |
| longitudinal time effect | *-0.03* | *-0.02* | *-0.015* | *-0.01* | *-0.005* | *0* |  |
| JM |  |  |  |  |  | 2.3 |  |
| joineRML | 28.4 | 25.3 | 23.7 | 21.3 | 22.8 | 17.3 |  |
| JMbayes2 |  |  |  |  |  | 660 |  |
| TS | 0.4 | 0.5 | 0.6 | 0.5 | 0.4 | 0.2 |  |
| tv cox | 0.1 | 0.1 | 0.1 | 0.1 | 0.1 | 0.1 |  |
|  |  |  |  |  |  |  |  |
| **Setting 5a** |  |  |  |  |  |  |  |
| SD error | *0.05* | *0.1* | *0.2* | *0.3* | *0.4* | *0.45* | *0.5* |
| JM | 6.5 | 6.5 |  |  |  |  |  |
| joineRML | 19.8 | 18.5 | 19.5 | 19.7 | 22.2 | 22.5 | 23.8 |
| JMbayes2 | 654.4 | 651.8 | 661.5 | 651.7 | 654.7 | 665.5 |  |
| TS | 0.2 | 0.2 | 0.3 | 0.3 | 0.3 | 0.3 | 0.3 |
| Tv cox | 0.1 | 0.1 | 0.1 | 0.1 | 0.1 | 0.1 | 0.1 |

Table 1. Mean computation times for linear simulation scenarios measured in seconds.tv cox, time-varying Cox proportional-hazards regression; TS, two-stage approach; JM and joineRML, frequentist joint models; JMbayes2, Bayesian joint model. Based on 200 simulations.

# Coverage probabilities for alpha

| **Setting 1** |  |  |  |  |  |  |  |
| --- | --- | --- | --- | --- | --- | --- | --- |
| *distance observations* | *20* | *60* | *100* | *140* | *200* | *250* | *300* |
| JM | 75.6 | 78.6 | 73.3 | 77.2 | 67.3 | 71.4 | 61.6 |
| joineRML | 96.5 | 96.5 | 96.5 | 97,2 | 96.6 | 95.5 | 95.0 |
| JMbayes2 | 96.0 | 95.5 | 96.0 | 96.1 | 96.2 | 95.9 | 94.5 |
| TS | 94.0 | 94.5 | 94.5 | 93.4 | 94.6 | 92.5 | 92.5 |
| tv cox | 90.0 | 94.5 | 92.0 | 92.5 | 94.0 | 93.5 | 93.0 |
|  |  |  |  |  |  |  |  |
| **Setting 2** |  |  |  |  |  |  |  |
| *alpha* | *-1.5* | *-1.25* | *-1* | *-0.75* | *-0.5* | *-0.25* | *0* |
| JM | 84.9 | 77.2 | 68.5 | 82.7 |  |  | 92.6 |
| joineRML | 95.0 | 95.5 | 94.5 | 95.7 | 96.5 | 92.9 | 94.4 |
| JMbayes2 | 98.2 | 98.6 | 95.5 | 95.8 | 95.8 | 95.8 | 93.7 |
| TS | 94.5 | 95.5 | 93.0 | 94.8 | 93.8 | 91.7 | 91.1 |
| tv cox | 91.0 | 94.5 | 91.0 | 94.0 | 93.0 | 92.0 | 91.0 |
|  |  |  |  |  |  |  |  |
| **Setting 3** |  |  |  |  |  |  |  |
| *baseline hazard* | *1.6* | *1.8* | *2* | *2.2* | *2.4* | *2.6* | *2.8* |
| JM | 87.2 | 76.9 | 70.8 |  |  |  |  |
| joineRML | 95.0 | 97.5 | 95.0 | 96.8 | 95.4 | 95.3 | 94.0 |
| JMbayes2 | 95.0 | 98.0 | 95.0 | 95.6 | 93.1 | 96.8 | 93.7 |
| TS | 89.0 | 96.0 | 93.0 | 96.2 | 92.6 | 92.0 | 91.6 |
| tv cox | 90.4 | 95.0 | 91.5 | 93.5 | 89.5 | 89.5 | 88.5 |
|  |  |  |  |  |  |  |  |
| **Setting 4** |  |  |  |  |  |  |  |
| *longitudinal time effect* | *-0.03* | *-0.02* | *-0.015* | *-0.01* | *-0.005* | *0* |  |
| JM |  |  |  |  |  | 95.5 |  |
| joineRML | 94.7 | 95.6 | 94.8 | 96.7 | 97.0 | 96.0 |  |
| JMbayes2 |  |  |  |  |  | 96.5 |  |
| TS | 92.6 | 94.1 | 92.8 | 95.4 | 94.4 | 94.0 |  |
| tv cox | 89.0 | 90.0 | 90.5 | 90.5 | 91.5 | 94.0 |  |
|  |  |  |  |  |  |  |  |
| **Setting 5** |  |  |  |  |  |  |  |
| SD measurement error | *0.05* | *0.1* | *0.2* | *0.3* | *0.4* | *0.45* | *0.5* |
| JM | 73.4 | 73.9 |  |  |  |  |  |
| joineRML | 96.5 | 96.5 | 96.5 | 96.0 | 97.0 | 97.5 | 96.0 |
| JMbayes2 | 95.5 | 96.5 | 92.5 | 96.4 | 97.7 | 94.4 |  |
| TS | 93.5 | 94.5 | 92.0 | 92.0 | 89.9 | 91.0 | 91.0 |
| tv Cox | 94.5 | 91.5 | 81.5 | 55.0 | 26.5 | 13.5 | 6.5 |

Table 2. Coverage probabilities for alpha estimate in simulation settings.tv cox, time-varying Cox proportional-hazards regression; TS, two-stage approach; JM and joineRML, frequentist joint models; JMbayes2, Bayesian joint model. Based on 200 simulations.

# Effective Sample Sizes JMbayes2

| **Setting 1a** |  |  |  |  |  | |  | |  |
| --- | --- | --- | --- | --- | --- | --- | --- | --- | --- |
| obs | 20 | 60 | 100 | 140 | 200 | 250 | | 300 | |
| ESS mean (SD) | 3454 (547.6) | 3356 (555.8) | 3294 (581.2) | 3181 (643) | 3027 (583.9) | 2732 (764.7) | | 2789 (647.1) | |
|  |  |  |  |  |  |  | |  | |
| **Setting 2a** |  |  |  |  |  |  | |  | |
| alpha | -1,5 | -1,25 | -1 | -0,75 | -0,5 | -0,25 | | 0 | |
| ESS mean (SD) | 1645 (868.7) | 2532 (695.5) | 3515 (480.5) | 4103 (513.5) | 4100 (704.9) | 2809 (1695.4) | | 2656 (1794.4) | |
|  |  |  |  |  |  |  | |  | |
| **Setting 3a** |  |  |  |  |  |  | |  | |
| baseline hazard | 1,6 | 1,8 | 2 | 2,2 | 2,4 | 2,6 | | 2,8 | |
| ESS mean (SD) | 1614 (1093.6) | 2431 (784.4) | 3412 (544.3) | 4059 (589.9) | 4107 (876.6) | 3131 (1770.3) | | 2659 (1797.9) | |
|  |  |  |  |  |  |  | |  | |
| **Setting 4a** |  |  |  |  |  |  | |  | |
| longit. time | -0,03 | -0,02 | -0,015 | -0,01 | -0,005 | 0 | |  | |
| ESS mean (SD) | 75 (19.2) | 78 (26.9) | 98 (22.7) | 139 (31.2) | 423 (73.3) | 4229 (555.3) | |  | |
|  |  |  |  |  |  |  | |  | |
| **Setting 5a** |  |  |  |  |  |  | |  | |
| SD error | 0,05 | 0,2 | 0,25 | 0,35 | 0,4 | 0,45 | | 0,5 | |
| ESS mean (SD) | 3420 (519.6) | 2522 (658.2) | 2045 (797.1) | 1210 (789.7) | 886 (693.2) | 613 (611.1) | | 443 (503) | |

Table 3. Effective Sample Sizes (ESS) for association parameter alpha in JMbayes2 based on 40,000 MCMC iterations plus burn-in of 5,000 and thinning of 20 for 3 parallel chains.

# Comparing JMbayes2 and two stage parameter estimates


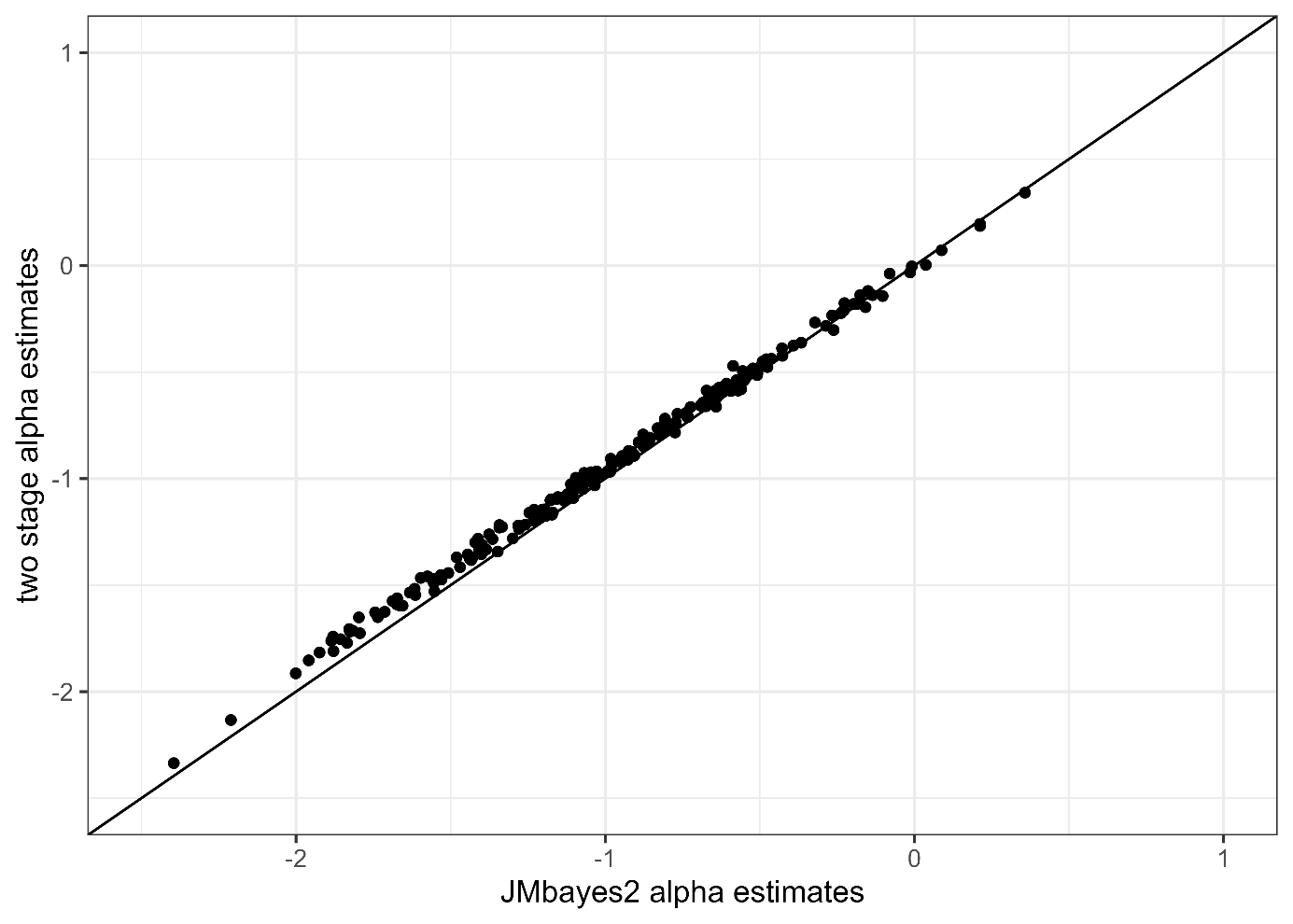


Figure 36 Comparing alpha estimates from two-stage approach (y axis) and JMbayes2 (x axis). Line is angle bisector. JMbayes2, Bayesian joint model. Based on 200 simulations.
